# Models to estimate genetic gain of soybean seed yield from annual multi-environment field trials

**DOI:** 10.1007/s00122-023-04470-3

**Published:** 2023-11-21

**Authors:** Matheus D. Krause, Hans-Peter Piepho, Kaio O. G. Dias, Asheesh K. Singh, William D. Beavis

**Affiliations:** 1 Department of Agronomy, Iowa State University, Ames, IA USA; 2 Biostatistics Unit, University of Hohenheim, Stuttgart, Germany; 3 Department of General Biology, Federal University of Viçosa, Viçosa, Brazil

## Abstract

**Key message:**

Simulations demonstrated that estimates of realized genetic gain from linear mixed models using regional trials are biased to some degree. Thus, we recommend multiple selected models to obtain a range of reasonable estimates.

**Abstract:**

Genetic improvements of discrete characteristics are obvious and easy to demonstrate, while quantitative traits require reliable and accurate methods to disentangle the confounding genetic and non-genetic components. Stochastic simulations of soybean [*Glycine max* (L.) Merr.] breeding programs were performed to evaluate linear mixed models to estimate the realized genetic gain (RGG) from annual multi-environment trials (MET). True breeding values were simulated under an infinitesimal model to represent the genetic contributions to soybean seed yield under various MET conditions. Estimators were evaluated using objective criteria of bias and linearity. Covariance modeling and direct versus indirect estimation-based models resulted in a substantial range of estimated values, all of which were biased to some degree. Although no models produced unbiased estimates, the three best-performing models resulted in an average bias of $\pm\, 7.41$ kg/ha$^{-1}$/yr$^{-1}$ ($\pm\, 0.11$ bu/ac$^{-1}$/yr$^{-1}$). Rather than relying on a single model to estimate RGG, we recommend the application of several models with minimal and directional bias. Further, based on the parameters used in the simulations, we do not think it is appropriate to use any single model to compare breeding programs or quantify the efficiency of proposed new breeding strategies. Lastly, for public soybean programs breeding for maturity groups II and III in North America, the estimated RGG values ranged from 18.16 to 39.68 kg/ha$^{-1}$/yr$^{-1}$ (0.27–0.59 bu/ac$^{-1}$/yr$^{-1}$) from 1989 to 2019. These results provide strong evidence that public breeders have significantly improved soybean germplasm for seed yield in the primary production areas of North America.

**Supplementary Information:**

The online version contains supplementary material available at 10.1007/s00122-023-04470-3.

## Introduction

The purpose of plant breeding is to improve genetic contributions to plant characteristics. For many discrete characteristics such as fruit and seed color, pubescence, herbicide and disease resistance, etc., the genetic improvements are obvious and easy to demonstrate. However, for continuous characteristics, such as seed or grain yield, the genetic contributions are incremental and difficult to separate from management practices or variable and changing environments. Plant breeders have used various experimental and statistical methods to estimate incremental genetic improvements of quantitative traits (e.g., Rincker et al. 2014; Byrum et al. 2017). However, with the exception of Rutkoski (2019b), the various proposed methods have not been compared using objective criteria. Herein we utilize a simulation approach to propose and investigate linear mixed models to obtain estimates of genetic improvements for quantitative traits.

Phenotypes (*P*) of traits evaluated on continuous scales are composed of discretely inherited polygenic effects (*G*), continuous environmental effects (*E*), and *GE* interaction effects (GEI) (Fisher 1918; Mayr 1942; Sprague and Federer 1951; Lynch and Walsh 1998). Further, the simple linear model $P = G + E + GE$ has been successfully used to investigate responses to selection (Johannsen 1911; Tabery 2008). The genetic component *G* is modeled as the sum of additive effects of alleles at multiple loci and non-additive genotypic effects (dominance and epistatic). For diploid species, additive effects refer to those associated with discrete alleles that can be inherited across generations, while non-additive genetic effects refer to genotypes that are not transmitted through inheritance. Thus, cycles of selection and reproduction affect genetic variability only through inherited alleles, also known as breeding values (Kempthorne 1957; Falconer 1960). In other words, realized genetic gain (RGG) is the improvement in average genetic (breeding) values due to the accumulation of favorable alleles through recurrent cycles of selection (Hazel and Lush 1942; Walsh and Lynch 2018; Rutkoski 2019a).

Applications of this theory were originally demonstrated using population improvement methods (Jenkins 1940). An original sample drawn from an unselected population is known as the founder population and is referred to as cycle zero (C0). Based on evaluated phenotypic values, a subset from C0 is selected to be randomly inter-crossed. The progeny generated by inter-crossing among the selected group represents a Mendelian sample of all possible progeny from the selected alleles, and is referred to as the C1 population. The process used to create C1 is reiterated with a sample from the C1 population to create C2, which is used to create C3, etc. The process is continuous and the expected outcomes are populations of genetically improved individuals.

Within a cycle of population improvement, the difference between the average phenotypic value of the population and the average of a selected sub-group is known as the selection differential (*S*). The difference between the averaged phenotypic values representing two consecutive cycles of recurrent selection is known as the realized response to selection (*R*). Thus, under an additive (linear) model (Fisher 1918), *R* equals the mean breeding value of members that are selected for inter-crossing (Walsh and Lynch 2018), and the relationship between *S* and *R* is given by the breeder’s equation: $R = h^2S$, where $h^2$ is the narrow-sense heritability (Lush 1937). Because only breeding values, i.e., additive allelic effects, are transmitted from one cycle to the next (for diploid species), the ratio *R*/*S* assumes values between zero and one and is known as the realized heritability ($h^2_R$) (Hallauer and Miranda 1988; Bernardo 2020). The $h^2_R$ associated with *R* and *S* can be used in the breeder’s equation to obtain an estimate of RGG. Alternatively, it has been suggested that RGG can be estimated from the slope of the regression line of average breeding values on breeding cycles across time (Eberhart 1964; Falconer and Mackay 1996; Rutkoski 2019a; Fritsche-Neto et al. 2023).

While RGG has been used to compare various population improvement methods in maize (Jenkins 1940; Hallauer and Miranda 1988; Dudley and Lambert 2004), since about 1980, population improvement breeding methods have not been used routinely to develop competitive hybrids. Rather, various types of cultivar development methods have been developed for maize and most commodity crops (Singh et al. 2021). Nonetheless, Duvick and co-workers demonstrated that previously sold maize hybrids could be used to assess progress in proprietary hybrid maize breeding programs (e.g., Duvick 1977, 1984, 2005). Their approach consisted of selecting a few widely grown maize hybrids to represent time periods (eras), and evaluating these in replicated field trials conducted in a common set of environments (location-year combinations). The estimated genotypic values for hybrids were regressed across the years when the hybrids were released to obtain an estimated trend (slope). As with maize, RGG in soybean has been estimated using “era trials” (Wilcox 2001; Ustun et al. 2001; Fox et al. 2013; Rincker et al. 2014; Rogers et al. 2015; Felipe et al. 2016; Bruce et al. 2019; Milioli et al. 2022).

Because era trials use designed experiments in which widely grown cultivars represent a selected “treatment,” replicated across the same set of environments, treatments and environments are orthogonal. However, inferences from era trials are limited because there are only a select few cultivars representing each era and they are evaluated only in a few environments. These do not represent a random sample of genotypes used to create the breeding populations. Thus, changes in the genotypic component across time represent an estimated commercial gain, which is not directly interpreted as an estimate of RGG (see Discussion). Further, the years in which the trials are conducted will favor newer cultivars that are more likely to be adapted to the environments in which the era trial is conducted (Piepho et al. 2014; Rizzo et al. 2022). Therefore, there is likely an environmental bias that favors and is confounded with recently released cultivars.

Alternatively, historical field data from annual multi-environment trials (MET) used to evaluate genotypes during their development have been used to assess changes across time for common bean (de Faria et al. 2018), potato (Ortiz et al. 2022), rice (Breseghello et al. 2011; Streck et al. 2018), rye (Laidig et al. 2017), sugarcane (Ellis et al. 2004), sunflower (de la Vega et al. 2007), wheat (Crespo-Herrera et al. 2018; Gerard et al. 2020), among other commercial crops and forage grasses (Piepho et al. 2014; Laidig et al. 2014). The advantages of using historical MET records are that the data (i) are usually available in stored repositories and (ii) represent larger samples of both genotypes and environments. However, historical MET usually have low connectivity among genotypes across environments because most experimental genotypes are culled annually. Thus, the disadvantages are that (i) environmental effects are mostly estimated from a relatively small set of selected experimental genotypes and a set of check cultivars, and (ii) new breeding programs usually do not have large historical datasets (Covarrubias-Pazaran 2020).

The most widely used method for partitioning genetic and non-genetic effects is the linear mixed model (LMM, Henderson 1949, 1950; Henderson et al. 1959). For a LMM, Best Linear Unbiased Estimators (BLUE) and Best Linear Unbiased Predictors (BLUP) are used to obtain estimates of fixed effects and predictions of random effects, respectively. In a Frequentist framework, both estimators usually utilize Residual Maximum Likelihood (REML, Patterson and Thompson 1971) to estimate variance components, yielding estimated/predicted empirical values (i.e., eBLUE and eBLUP values). In general, genetic trends are computed from the regression of the eBLUE or eBLUP values of genotypes on the first year of testing in MET, or the year of cultivar release for era studies. Alternatively to the application of LMM, algorithmic modeling (Byrum et al. 2017) or a combination of algorithmic and linear modeling have been proposed to remove non-genetic effects in order to estimate RGG (Brisson et al. 2010; Oury et al. 2012; Bornhofen et al. 2018).

A simulation approach designed to evaluate the accuracy and precision of estimators of RGG using historical MET data was conducted by Rutkoski (2019b), where simulation parameters were obtained from low-budget cultivar development programs conducted by the International Rice Research Institute. In total, the author simulated 80 *indica*-type rice breeding programs assuming two levels of heritability (low and high), the inclusion of either a positive or negative non-genetic trend linked to the calendar year (i.e., years of breeding operation), and two breeding schemes. The RGG was then estimated for a quantitative trait composed of additive effects at 1000 loci from simulated era and yield trials with different modeling strategies based on LMM. The study concluded that (i) the evaluated estimators were inaccurate, (ii) the error associated with the estimates was dependent on the breeding scheme, non-genetic trend, and heritability, and (iii) if the goal is restricted to determine if RGG is greater than zero, some indicators like the expected rate of genetic gain and the equivalent complete generations are useful (Boichard et al. 1997).

Herein, we use a similar simulation approach as Rutkoski (2019b) to evaluate LMM estimators of RGG in simulated public soybean breeding programs adapted to Maturity Groups (MG) II and III in North America. It is evident the genotypic sampling space in a breeding program is complex due to multiple parental genotypes, families in early trials, check cultivars, experimental genotypes, and introductions of germplasm from external sources. In this context, the first step towards estimating RGG in cultivar development programs is to define the inference space. We define the breeding population as consisting of mostly homozygous genotypes (i.e., purelines) that are used as parents in crossing blocks. Estimates of genetic gains across time based on MET data are associated with lines that were or could be inter-crossed to create new families consisting of segregating self-pollinated genotypes for evaluation. As such, the breeding population represents a set of experimental lines from the tail of a distribution that has about twice the additive genetic variance expressed in the F$_2$ generation (Lynch and Walsh 1998; Bernardo 2020), plus lines from external sources that likewise have genotypic values in the tail of the distribution. Assuming that these best-performing lines have accumulated favorable alleles, i.e., have higher average breeding value compared to their parents, the genetic trends across years of MET are a function of the breeding values and therefore can be interpreted as an estimate of RGG for germplasm that is adapted to the conditions of the MET.

Simulations were based on knowledge of the organization of public soybean breeding programs for MG II and III in North America, as well as previously analyzed MET data (Krause et al. 2023). These data consist of 4257 genotypes evaluated in replicated field trials consisting of $\sim$ 20–34 experimental lines per trial. Trials within environments were conducted at 63 locations for 31 years, resulting in 591 observed environments (location-year combinations) from 1989 to 2019. Simulated values for RGG were computed according to the accumulation of favorable alleles in experimental lines evaluated in advanced MET. We also implemented in the simulator an alternative way to simulate non-genetic contributions to gain based on empirical frequencies of locations used in the historical records of MET. Thus, the simulator was built to (i) evaluate bias from LMM estimators of RGG based on simulations that emulated empirical data routinely collected by public soybean breeders, and (ii) investigate if these models can determine if there is any RGG regardless of estimated bias. Lastly, we report estimates of RGG for the soybean empirical dataset from Krause et al. (2023) using the best-performing models.

## Material and methods

### Soybean stochastic simulations

Simulations were conducted using AlphaSimR (Gaynor et al. 2021) and functions developed in the R programming environment (R Core Team 2021). The simulation code was run in parallel (3 cores per replicate) in a bash shell under Linux. Each simulation run represents an independent breeding program consisting of 46 years. On average, each simulation run took four hours to be completed in computer nodes with 30 GB of RAM and Intel processors with speeds ranging from 3.20 to 3.50 GHz. Statistical analyses were performed using Asreml-R version 4.1 (Butler et al. 2017) and R base functions. Values for the simulation parameters (e.g., initial trait mean, variance components, number of crosses and genotypes, etc.) were obtained from analyses of historical MET soybean data from Krause et al. (2023).

#### Founder population

A founder population was created from a set of 499 pureline soybean genotypes developed by public soybean breeders for maturity groups II and III. These lines were genotyped using the SoySNP6K BeadChip (Song et al. 2020). Single nucleotide polymorphism markers (SNP) were removed if missing scores were $>20$%, or if the minor allele frequency was $< 5\%$. Lines were removed if heterozygosity $>6.25\%$ and/or $>20$% of the markers were missing data. The final number of SNP markers was 5279, ranging from 204 to 349 markers per chromosome. A principal components analysis of the additive genomic relationship matrix ${\textbf {G}}_{{\textbf {M}}}$ (Endelman and Jannink 2012) does not show evidence of grouping among this set of possible founders (Supplementary Material Figure A1).

#### Breeding values

Simulated breeding values were obtained assuming Fisher’s infinitesimal model to approximate polygenic effects on soybean seed yield. Eligible additive quantitative trait loci (QTL) were randomly assigned to 1000 loci distributed across 20 chromosomes, according to a genetic linkage map (2145.5 cM) estimated from the Soybean Nested Association Mapping population (Ramasubramanian and Beavis 2020). Further details are given in Supplementary Material.

#### Crossing nurseries and development of experimental lines

Every year 50–80 biparental crosses between 20–30 selected genotypes (lines) were simulated to produce a population of F$_1$ individuals. Each F$_1$ individual was self-pollinated to create 50–80 simulated families of F$_2$ seeds (S$_{0}$ generation), each representing segregating progeny descended from a biparental cross. Note, for hybrid crop species we also utilize the ‘S’ symbol to represent selfing generation in line development projects within heterotic groups. From each F$_2$ individual plant, 2–3 F$_3$ seeds were obtained from simulated self-pollinations to represent the S$_{1}$ generation. Each F$_3$ individual was self-pollinated to produce F$_4$ seeds representing the S$_{2}$ generation. All of the self-pollinated seed from each F$_4$ individual was combined to represent an F$_{4:5}$ experimental line. Selections were not simulated during the development of the F$_{4:5}$ experimental lines.

#### Selection across stages and field trials

**Fig. 1:**
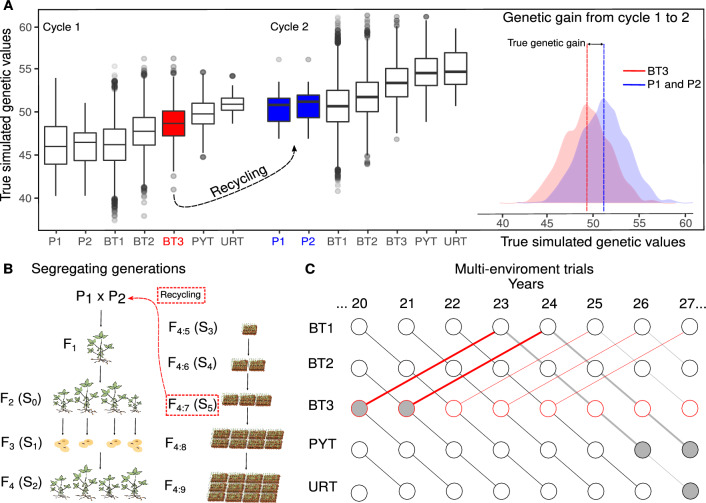
Graphic depiction of the simulated RGG between cycles 1 and 2 of line development. **A** Experimental lines selected from BT3 were crossed in a nursery to create a new population that was used to develop experimental lines for evaluation in the subsequent cycle of evaluation. **B** Experimental lines evaluated in PYT and URT include experimental lines selected from BT3 for crossing. **C** For example, breeding lines selected in BT3 of year 20 are evaluated *per se* in PYT in year 21, and will have progeny developed as experimental lines evaluated in the PYT of year 26

Phenotypic values of F$_{4:5}$ experimental lines were simulated for evaluation in an unreplicated field trial at a single location, designated as breeders trial 1 (BT1). Self-pollinated seeds (F$_{4:6}$) from selected F$_{4:5}$ experimental lines were subsequently evaluated at two locations (BT2). Self-pollinated seeds (F$_{4:7}$) from the selected F$_{4:6}$ experimental lines were then evaluated at three locations (BT3). The best F$_{4:7}$ experimental lines selected from the BT3 were then evaluated in regional trials. The first year of regional trials consisted of F$_{4:8}$ experimental lines evaluated at eight locations and was referred to as the preliminary yield test (PYT). If selected, F$_{4:8}$ experimental lines were evaluated in the uniform regional test (URT) at 12 locations. Experimental lines in PYT and URT (i.e., advanced MET) can be thought of as candidate varieties (Fig. 1B, C).

Ten percent of the BT1 experimental lines were selected for evaluation in BT2. For the remaining stages, a 20% selection intensity was applied. Yearly selections were carried out by ranking the predicted eBLUP values of experimental line means obtained only from the data of the current year. Experimental lines in the BT1 phase of development were genotyped with 2400 SNP markers, although the marker genotypes were not used for genomic selection. Rather, SNP information was used by some of the LMM to estimate RGG by providing information about covariance across cycles of development.

**Fig. 2:**
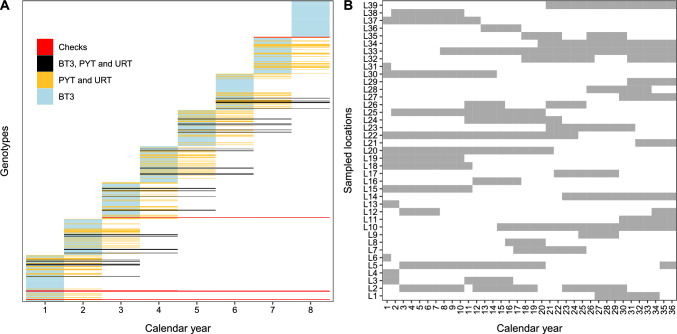
**A** Representation of the simulated selection process in MET and **B** the rate of locations replacement across years

Field trials within evaluation stages (BTs, PYT and URT) included three to six check cultivars, where the actual number of checks was randomly determined for each simulation (Fig. 2A and Supplementary Material Figure A2). Historical records of MET represent summaries of trials that were conducted using a randomized complete block design with two or three blocks per trial, but the summary does not include individual plot and block information. Rather, the summaries report eBLUE values for each genotype (Krause et al. 2023). In other words, reported genotypic values and estimates of residual variance for each trial are adjusted for block effects. Thus, in order to simulate using similar values for residual variance, the field plot design for each trial was completely randomized with a single replicate for BT1, two replicates for BT2, and three replicates per location for the remaining trials. A random percentage from zero to 12% of individual field plots were considered to be missing data within each trial. The number of locations for each trial was fixed, although actual simulated locations were replaced across years to mimic the practice of occasionally changing locations in a region from year to year (Figs. 2B and Supplementary Material Figure A3). The models used for the selection of experimental lines are provided in Supplementary Material.

#### Selection of breeding lines and cycle of line development

BLUP selection of breeding (parental) lines was performed in BT3 with combined phenotypic data from BT1, BT2, and BT3 (Model C1, Supplementary Material). Assuming that the creation of the experimental lines prior to evaluation in BT1 could be conducted at off-site continuous nurseries with 3 seasons per year, the time per cycle of line development would be five years. Explicitly, it will require two years to begin with the initial crosses, create F$_{1}$ seed, and subsequently develop the F$_{4:5}$ experimental lines for evaluation on-site in BT1. Subsequently, we assumed that there is only one growing season for evaluating the crop in the breeder’s trials (BT’s) (Fig. 1B). Breeding lines were not selected in the first five years of the simulated breeding programs (i.e., not enough data). Rather, the simulated crosses were randomly sampled from the founder population. Moreover, these initial years were not used to estimate RGG (Supplementary Material Figure A4).

#### Simulations of GEI in MET

Genotype by environment effects were defined as the sum of genotype $\times$ location (${\textit{GL}}$), genotype $\times$ year (${\textit{GY}}$), and genotype $\times$ location $\times$ year (${\textit{GLY}}$) interaction effects, i.e., ${\textit{GEI}} = {\textit{GL}} + {\textit{GY}} + {\textit{GLY}}$. Genotype refers to experimental lines. QTL effects were assigned to genotypes at the beginning of simulation runs and retained through all stages of line development. The simulation of the main genotypic effects (*G*) was accomplished using AlphaSimR as described in section “Breeding values”, with the matrix of additive QTL genotypes being used to simulate GEI effects. Individual plot phenotypic values were simulated according to the following general linear model:

1 $$\begin{aligned} Y_{ijkl} = \mu + G_i + L_j + Y_k + {\textit{GL}}_{ij} + {\textit{GY}}_{ik} + LY_{jk} + {\textit{GLY}}_{ijk} + \epsilon _{ijkl} \end{aligned}$$

where $Y_{ijk}$ is the simulated phenotype for the *ijk*th genotype (line) $\times$ location $\times$ year combination in the *l*th plot, $\mu$ is the intercept or mean trait value, and $\epsilon _{ijkl}$ is the residual plot-to-plot variability associated with the simulated phenotypic value. The remaining terms *L* and *Y* represent the locations and years’ main effects, respectively. All model terms except *L* were considered random effects sampled from various distributions described below (section “Variations of simulated MET models”). Locations were simulated as fixed effects to incorporate estimated values from empirical data (Supplementary Material Figure A5). Individual trial effects were not simulated.

##### Variations of simulated MET models

We used six simulation models for MET and labeled them A1, A2, B1, B2, B2-M, and B2-R. The “A” conditions represent simple genetic effects models where random effects from Model (1) were simulated as independent with homogeneous variances. The “B” conditions represent models where each simulation run ($s = 1, \dots , S = 225$) had a unique *G* ($\sigma _{G_s}^2$) and ${\textit{GLY}}$ ($\sigma _{{\textit{GLY}}_s}^2$) variance component. Also, the B model simulations consisted of correlated additive QTL effects across locations and years for the *GL* and *GY* interaction effects, respectively. The “1” versions of these models had no non-genetic trend, while the “2” versions did (see below). Simulations labeled B2-M are a modification of B2 where the sampled variance values for *GL*, *GY*, and *GLY* were divided by an *ad-hoc* factor of 10 to minimize the contribution of GEI components. Lastly, simulations labeled B2-R represent B2 where a random sample of experimental and breeding lines were retained (i.e., no selection). Heterogeneous residual variances represented plot-to-plot variability within trials and were sampled from a Log-Logistic distribution (Tables 1 and 2).

The GEI simulation approach for the “B” simulation models was developed to provide similar results as encountered in empirical data analyzed by Krause et al. (2023). A step-by-step description of the simulation of the correlated QTL effects is given in Supplementary Material. Briefly, define ${}_{\varvec{I}}\varvec{\Theta }_{\varvec{1000}}$ as the matrix of QTL dosages (0, 1, and 2) simulated by AlphaSimR. The matrix $\varvec{\Theta }$ has dimension $I \times 1000$, where *I* $(i = 1, \dots , I)$ represents the number of genotypes and $q = 1000$ is the number of simulated QTL. The sampled effects are defined in matrix notation as ${}_{\varvec{q}}\varvec{\phi }^{\varvec{G}}_{\varvec{1}}$, ${}_{\varvec{q}}\varvec{\phi }^{\varvec{GL}}_{\varvec{J}}$, ${}_{\varvec{q}}\varvec{\phi }^{\varvec{GY}}_{\varvec{K}}$, and ${}_{\varvec{q}}\varvec{\phi }^{\varvec{GLY}}_{\varvec{JK}}$ for model terms $G, {\textit{GL}}, {\textit{GY}}$, and ${\textit{GLY}}$, respectively. Their dimensions are represented by *I*, *J*, *K* for genotypes, locations ($j = 1, \dots , J$), and years ($k = 1, \dots , K$), respectively. Note ${}_{\varvec{q}}\varvec{\phi }^{\varvec{G}}_{\varvec{1}}$ is a vector of length 1000 as defined in section “Breeding values”. This notation is general and reflects the sample size associated with the model parameters in each trial (Tables 1 and 2).

**Table 1:** Variance component values and probability distributions implemented in the simulator

Effect	Variance component^a,b^	
	Models A1 and A2	Models B1 and B2
Genotypic (*G*)	6.3	Log-Logistic (5)
Year (*Y*)	15.3	15.3
*GL*	4.8	Log-Logistic (3)
*GY*	3.2	Log-Logistic (6)
*LY*	81.4	81.4
*GLY*	10.1	Gamma (5)
Trial error ($\epsilon _{ijk}$)	Log-Logistic (1)	Log-Logistic (1)

^a^Numerical values are point estimates in units of (bu/ac)$^2$. Locations were considered fixed effects (Supplementary Material Figure A5)

^b^Log-Logistic (*n*) and Gamma (*n*) are empirical density functions, where *n* refers to the number of mixture models. A full description of parametric density functions for variance components is given in Supplementary Material

**Table 2:** Fixed effect and distributional assumptions for random effects implemented in the simulator. *G*, *L*, and *Y* represent genotypic (line), location, and year factors. Note the sampling for the genotypic-related terms *G*, *GL*, *GY*, and *GLY* were applied at the level of simulated QTL

Model terms	Simple effects^a^	Complex effects^a^
	Simulation models A1 and A2^b^	Simulation models B1 and B2^b^
Intercept ($\mu$)	46	46
Genotypic (*G*)	$\varvec{{}_{I}\Theta _{q}} \cdot \varvec{{}_{q}\phi ^G_{1}} \text {, where } \varvec{{}_{q}\phi ^G_{1}} \sim N(\varvec{0_q}, \sigma _{G}^2{\textbf {I}}_q)$	$\varvec{{}_{I}\Theta _{q}} \cdot \varvec{{}_{q}\phi ^G_{1}} \text {, where } \varvec{{}_{q}\phi ^G_{1}} \sim N(\varvec{0_q}, \sigma _{G{_s}}^2{\textbf {I}}_q)$
Location (*L*)	Fixed	Fixed
Year (*Y*)	$N(\varvec{0}_K, \sigma _{Y}^2{\textbf {I}}_K)$	$N(\varvec{0}_K, \sigma _{Y}^2{\textbf {I}}_K)$
*GL*	$\varvec{{}_{I}\Theta _{q}} \cdot \varvec{{}_{q}\phi ^{GL}_{J}} \text {, where } \varvec{{}_{q}\phi ^{GL}_{J}} \sim N(\varvec{0_J}, \sigma _{GL}^2{\textbf {I}}_{\varvec{J}})$	$\varvec{{}_{I}\Theta _{q}} \cdot \varvec{{}_{q}\phi ^{GL}_{J}} \text {, where } \varvec{{}_{q}\phi ^{GL}_{J}} \sim N(\varvec{0_J}, \varvec{\Sigma _J}{_s})$
*GY*	$\varvec{{}_{I}\Theta _{q}} \cdot \varvec{{}_{q}\phi ^{GY}_{K}} \text {, where }\varvec{{}_{q}\phi ^{GY}_{K}} \sim N(\varvec{0_K}, \sigma _{GY}^2{\textbf {I}}_{\varvec{K}})$	$\varvec{{}_{I}\Theta _{q}} \cdot \varvec{{}_{q}\phi ^{GY}_{K}} \text {, where } \varvec{{}_{q}\phi ^{GY}_{K}} \sim N(\varvec{0_K}, \varvec{\Sigma _K}{_s})$
*LY*	$N(\varvec{0}, \sigma _{LY}^2{\textbf {I}}_{JK})+(z_{jk} \times \eta _j)$	$N(\varvec{0}, \sigma _{LY}^2{\textbf {I}}_{JK})+(z_{jk} \times \eta _j)$
*GLY*	$\varvec{{}_{I}\Theta _{q}} \cdot \varvec{{}_{q}\phi ^{GLY}_{JK}} \text {, where } \varvec{{}_{q}\phi ^{GLY}_{JK}} \sim N(\varvec{0_{JK}}, \sigma _{GLY}^2{\textbf {I}}_{\varvec{JK}})$	$\varvec{{}_{I}\Theta _{q}} \cdot \varvec{{}_{q}\phi ^{GLY}_{JK}} \text {, where } \varvec{{}_{q}\phi ^{GLY}_{JK}} \sim N(\varvec{0_{JK}}, \sigma _{GLY_{s}}^2{\textbf {I}}_{\varvec{JK}})$
Trial error^c^	$N(\varvec{0}, \oplus _{b =1}^{B}\sigma ^2_{\epsilon _b}{} {\textbf {I}}_{n_b})$	$N(\varvec{0}, \oplus _{b =1}^{B}\sigma ^2_{\epsilon _b}{} {\textbf {I}}_{n_b})$

^a^The matrix $\varvec{\Theta }$ has dimension $I \times 1000$, where $I (i = 1, \dots , I)$ represents the number of genotypes and $q = 1000$ is the number of simulated QTL. The sampled effects are defined in matrix notation as $\varvec{{}_{q}\phi ^G_{1}}$, $\varvec{{}_{q}\phi ^{GL}_{J}}$, $\varvec{{}_{q}\phi ^{GY}_{K}}$, and $\varvec{{}_{q}\phi ^{GLY}_{JK}}$ for model terms *G*, *GL*, *GY*, and ${\textit{GLY}}$, respectively. Their dimensions are represented by *I*, *J*, *K* for genotypes, locations ($j = 1, \dots , J$), and years ($k = 1, \dots , K$), respectively. Note $\varvec{{}_{q}\phi ^G_{1}}$ is a vector of length 1000 and it was simulated as defined in section “Breeding values”. *N* represent the Multivariate Normal distribution and $\cdot$ matrix multiplication. The matrices $\varvec{\Sigma _J}{_s}$ and $\varvec{\Sigma _K}{_s}$ are positive-definite covariance matrices for *GL* and *GY* effects, respectively. $\varvec{0}$ is a vector of zeros and ${\textbf {I}}$ an identity matrix with their respective dimensions

^b^The interaction *LY* simulates the inclusion of a positive non-genetic trend in models A2 and B2, according to the mapping variable $z_{jk}$ described in Eq. (2). In scenarios A1 and B1, the non-genetic trend was not simulated, hence $z_{jk}$ was absent. For details, see section “Simulation of the non-genetic trend”

^c^Trials ($b = 1, \dots , B$, with a sample size of $n_b$) were simulated with heterogeneous residual variances ($\sigma ^2_{\epsilon _b}$) based on empirical evidence from Krause et al. (2023). $\oplus$ is the direct sum operator

##### Simulation of the non-genetic trend

Simulation models A2 and B2 include a positive non-genetic trend whereas models A1 and B1 do not (Tables 1 and 2). To simulate a positive non-genetic trend, we proposed that the location $\times$ year interaction term ($LY_{jk}$) from Model (1) be further split into two components:

2 $$\begin{aligned} LY_{jk} = u_{jk} + (z_{jk} \times \eta _j),\quad \text {for}\quad \eta _j \in \left( \frac{1}{2}, \frac{1}{3}, \frac{1}{4}, \frac{1}{5}, \frac{1}{6}\right) \end{aligned}$$

where $u_{jk} \sim N(0, \sigma ^2_{LY})$, $z_{jk}$ is a location–year covariate mapping if the *j*th location was observed in the *k*th and $(k+1)$th years, and $\eta _j$ is a constant of the non-genetic gain randomly chosen with equal probability. Equation (2) was designed to simulate a (cumulative) positive non-genetic trend by adding increments of $\eta _j$ units (bu/ac) every time a specific location was observed across consecutive years. This model was used to represent improvements in management practices in field trials that are continuously used by the breeding program, and is linked to the location $\times$ year effect. For example, suppose location “L1” is used for yield evaluations in the current year, and the farmer/researcher identifies regions of low fertility at L1. The issue will be addressed and the yield at L1 in subsequent years will likely improve. For clarity, an example of the covariate mapping is provided in Tables 3 and 4.

Annual effects ($Y_k$) were assumed to be random: there are favorable (positive increments in yield) and unfavorable (negative increments in yield) years. This was simulated by randomly sampling year effects from a Normal distribution (Table 1). If the cumulative non-genetic trend is not simulated, the term $z_{jk}$ does not appear, and therefore Eq. (2) is reduced to $LY_{jk} = u_{jk}$. In this case, the location–year effects are only a function of the random sample from the Normal distribution ($u_{jk}$).

**Table 3:** A hypothetical example of the covariate mapping ($z_{jk}$) used to simulate cumulative ($z_{jk} \times \eta _j$) rates ($\eta _j$) of non-genetic gain

Calendar year	Is the *j*th location used?	$z_{jk}$	$\eta _j$	How many years has the *j*th location been used?	Cumulative non-genetic gain ($z_{jk} \times \eta _j$)
1	Yes	0	0	1	0
2	No	–	–	1	–
3	Yes	1	1/2	2	1/2
4	Yes	2	1/2	3	$1/2+1/2$
5	No	–	–	3	–
6	No	–	–	3	–
7	Yes	3	1/2	4	$1/2+1/2+1/2$

**Table 4:** Hypothetical example of $L_j + Y_k + LY_{jk}$ from Model (1) when the non-genetic trend is included

$L_j + Y_k + LY_{jk}$	$\Rightarrow$	Fixed effect	+	$Y_k \sim N(0, \sigma ^2_Y)$	+	$LY_{jk} \sim N(0, \sigma ^2_{LY})$	+	$(z_{jk} \times 0.5)$	
	$\Rightarrow$	+ 3		− 2.27		− 17.42	+	($0\times 0.5$)	[Year 7]
	$\Rightarrow$	+ 3		+ 2.79		− 10.40	+	($1 \times 0.5$)	[Year 8]
	$\Rightarrow$	+ 3		+ 3.22		+ 4.23	+	($2 \times 0.5$)	[Year 9]
		$\vdots$		$\vdots$		$\vdots$		$\vdots$	$\vdots$
	$\Rightarrow$	+ 3		+ 9.03		+ 5.53	+	($15 \times 0.5$)	[Year 22]

#### Simulated “true” RGG values

The simulated “true” values of RGG were calculated from the genetic values of lines used for crossing. Explicitly, it was calculated as the slope ($\beta _{T_{(s)}}$) of the regression line of true genetic values of breeding lines ($g_{T_{i(s)}}$) on the year they were used in crossing blocks ($w_{i_{(s)}}$):

3 $$\begin{aligned} g_{T_{i(s)}}&= \beta _{0_{(s)}} + \beta _{T_{(s)}} w_{i_{(s)}} + \epsilon _{T_{i(s)}}, \quad \text {where}\nonumber \\&\quad \beta _{0_{(s)}}\, \text {is the intercept and}\, \epsilon _{T_{i(s)}} \sim \, \text {N}(0, \sigma ^2_{\epsilon _{T(s)}}) \end{aligned}$$

The slope $\beta _{T_{(s)}}$ represents the rate of accumulation of beneficial (additive) alleles among breeding lines across years of breeding operation. A breeding line may be crossed multiple times (hub network crossing design), but only in a single crossing nursery/year.

### Data and estimation of RGG

Historical data records have the potential to provide four possible data sets generated in the line development process: (i) the most advanced set of experimental lines evaluated in the URT, (ii) data from experimental lines that are first evaluated in PYT and subsequently in the URT, (iii) data from BT3, PYT, and URT that provide information from three years of trials for the most advanced experimental lines as well as information from breeding lines (i.e., pedigree information from the previous cycle), and (iv) data from all MET stages (BTs, PYT, URT). The first and second data sets are typical of historical data available for most crop species. For our simulations, we also evaluated the third data set to determine if estimating RGG exclusively from breeding lines would provide more accurate estimates. The fourth data set was not considered in this study.

Check cultivars were included in the models to provide estimates of non-genetic trends across years but were not considered to estimate RGG. The estimation models can be classified as providing either direct or indirect estimates of RGG, as they differ in the decomposition of the genotypic effects ($G_i$). The direct estimate was computed by replacing $G_i$ with $\beta _{g_{(s)}} r_{i_{(s)}}$, where $\beta _{g_{(s)}}$ is a heterogeneous regression coefficient of RGG for experimental lines, and $r_{i_{(s)}}$ is the year of first testing. The year of first testing is designated as the first year in which the phenotypic data of the *i*th experimental line is available. For public soybean empirical MET data, this occurs in the PYT. $\beta _{g(s)}$ is considered heterogeneous so that data from check cultivars do not contribute to the estimation of RGG. In contrast, indirect estimates of RGG require two analytic steps: The first step consisted of estimating/predicting genotypic main effects ($\hat{g}_{i_{(s)}}$), which can be either eBLUE or the eBLUP values for only the experimental lines. The second step estimates RGG as the slope ($\beta _{R_{(s)}}$) of the linear regression between $\hat{g}_{i_{(s)}}$ and $r_{i_{(s)}}$, defined as:

4 $$\begin{aligned} \hat{g}_{i_{(s)}}&= \beta _{0_{(s)}} + \beta _{R_{(s)}} r_{i_{(s)}} + \epsilon _{R_{i(s)}},\quad \text {where}\nonumber \\&\quad \beta _{0_{(s)}}\, \text {is the intercept and}\, \epsilon _{R_{i(s)}} \sim \, \text {N}(0, \sigma ^2_{\epsilon _{R(s)}}) \end{aligned}$$

Alternatively, when $\hat{g}_{i_{(s)}}$ are eBLUP values, RGG can indirectly be estimated with the cumulative sum of the average $\hat{g}_{i_{(s)}}$ values of all lines ($\varvec{\kappa }_{(s)}$):

5 $$\begin{aligned} \varvec{\kappa }_{(s)} = \left( \kappa _1, \kappa _1 + \kappa _2, \dots , \sum _{k=1}^{K} \kappa _k\right) ,\quad \text {where}\;\kappa _n = \frac{\sum _{i\in S{_k}} \hat{g}_{i}}{n_k} \end{aligned}$$

where *K* is the number of years in the data set, $\hat{g}_{i}$ is the predicted eBLUP value of the *i*th experimental line, $S_k$ is the set of lines first tested in the *k*th year, and $n_k$ is the number of experimental lines evaluated in $S_k$. The RGG is then estimated by regressing $\varvec{\kappa }_{(s)}$ on the year of first testing ($r_{i_{(s)}}$). An example is presented in Table 5.

**Table 5:** Hypothetical example of the cumulative sum ($\varvec{\kappa }$) of the average ($\kappa _n$) eBLUP values ($\hat{g}_{i}$) linked to the first year of trial ($r_{i}$) for nine experimental lines (ID)

ID	$r_{i}$	$\hat{g}_{i}$	$\kappa _n$	$\varvec{\kappa }$
G$_1$	1	+ 2.1	+ 0.8	+ 0.8
G$_2$		+ 1.0		
G$_3$		− 0.7		
G$_4$	2	+ 0.1	+ 1.0	+ 1.8
G$_5$		+ 3.4		
G$_6$		1.5		
G$_7$		− 1.0		
G$_8$	3	− 0.3	+ 1.8	+ 3.6
G$_9$		+ 3.9		

Thus, $\varvec{\kappa }_{(s)}$ is a generalization of the expected gain from selection (Walsh and Lynch 2018; Falconer and Mackay 1996). Variability from year to year (i.e., *GY*, *GLY*, *LY*, and *Y*) is accounted for because $\varvec{\kappa }_{(s)}$ is computed with a full, $G\times L\times Y$ model.

#### Estimation models applied to simulated PYT and URT

Twenty-one models were applied to simulated data sets consisting of PYT and URT (Table 6). The first model, designated EB (benchmark), indirectly estimated RGG with eBLUP values. Model E0 uses the same framework as EB, but RGG was estimated with $\varvec{\kappa }_{(s)}$. Model E1 (Mackay et al. 2011) estimated RGG with eBLUE values. Model E2 (Piepho et al. 2014) directly estimated RGG with a fixed effect regression coefficient, and Model E3 with a random regression coefficient. Models E4 and E5 are variations of Models E0 and E1, respectively, where RGG was estimated only from lines in URT (i.e., the last year of trial). Model E6 (control population, Rutkoski 2019b) directly estimated RGG ($\beta _t$) by contrasting checks and experimental lines with a fixed regression term ($\beta _t t_k$), where $t_k$ is a continuous covariate for the calendar year. Model E7 was designed to assess if environmental (the combination of location–year) effects will be properly modeled using check cultivars. An initial model is applied to the phenotypic data associated with only the check cultivars to obtain predicted values (i.e., eBLUP) of environmental effects. These predicted values are subsequently used as a fixed effect covariate in a second analysis model. In the second model, the eBLUP values of experimental lines are predicted and used to estimate RGG indirectly. Model E8 is a variation of E7, in which RGG was directly estimated in the second step.

We also included parameters for several covariance structures representing *GL* and *GY* interaction effects. Models E0, E1, E2, E3, E4, E5, and E6, assumed independent effects with homogeneous variance, whereas their counterparts E0V, E1V, E2V, E3V, E4V, E5V, and E6V, involved correlated random effects with heterogeneous variances. We restricted investigations of diagonal and first-order factor-analytic models to avoid convergence issues across simulation runs. For Models E0G, E0GV, and E7G, we also investigated the RGG estimation with genomic estimated breeding values (Table 6).

Model E9 takes advantage of both experimental lines and checks replicated between consecutive pairs of years: (i) an initial model was fit within years to obtain eBLUP values for experimental lines ($g_{ik}$); and (ii) the $g_{ik}$ values were then corrected for the “year effect” according to the “reference year (*R*).” For example, in a MET dataset of 10 years, if *R* = year **5**, the corrections for the year effects will be performed with a forward-backward regression algorithm among years, e.g., $1\leftarrow 2\leftarrow 3\leftarrow 4\leftarrow {\textbf {5}}\rightarrow 6\rightarrow 7\rightarrow 8\rightarrow 9\rightarrow 10$. If *R* = **1**, only a forward process (${\textbf {1}}\rightarrow \dots \rightarrow 10$) was used, and if *R* = **10**, only a backward process ($1\leftarrow \dots \leftarrow {\textbf {10}}$) was used (Table 6). The methodology is presented with references in Supplementary Material. In addition to the described models, RGG was also computed with Model 4 using raw phenotypes at each location (“Pheno”) to provide an estimate of phenotypic changes across years. Thus, it accesses the impact of estimating RGG without explicit models.

**Table 6:**
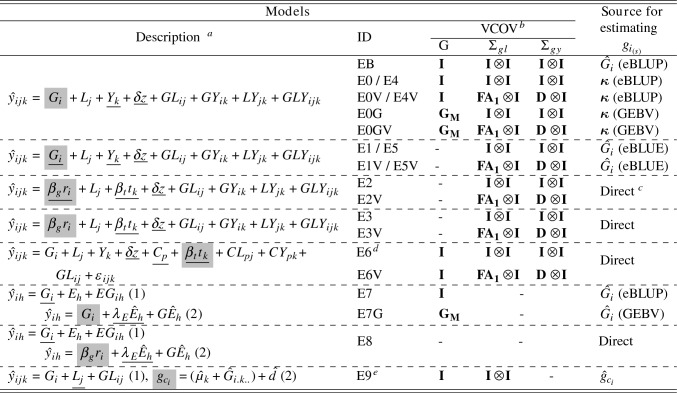
Models applied to simulated PYT and URT. Fixed effects are underlined and the RGG was estimated with model terms highlighted in gray

$^{\textrm{a}}$Model terms *G*, *L*, *Y*, and *E*, are the main effects of genotypes (lines), locations, years, and environments ($h = 1, \dots , H$), respectively. The response variables $\hat{y}_{ijk}$ and $\hat{y}_{ih}$ are the vectors of eBLUE values of experimental lines, such as found in historical records. The covariates *z* and $t_k$, with regression coefficients $\delta$ and $\beta _t$, represent variables of non-genetic trend linked to the frequency of locations (Eq. 2) and a continuous covariate for the calendar year, respectively. The covariate $\hat{E}$ is the predicted eBLUP values for *E*. The residual $\epsilon _{ijk}$ is included when stage-wise models were not used. Models E4 and E5 are similar to models E0 and E1, respectively, except that RGG for E4 and E5 were computed only with experimental lines in the last year of trial (i.e., URT). The numbers in parentheses (1, 2) in the model’s description represent steps to fit the models. Models in step one (1) were adjusted only with check cultivars

$^{\textrm{b}}$Variance-covariance matrices (VCOV) were modeled for *G*, *GL* ($\Sigma _{gl}$), and *GY* ($\Sigma _{gy}$). The evaluated VCOV were identity (**I**), diagonal (**D**), and factor-analytic (${\textbf {FA}}_{{\textbf {k}}}$) of order $k = 1$. ${\textbf {G}}_{{\textbf {M}}}$ stands for the additive genomic relationship matrix (Endelman and Jannink 2012). Models’ ID with “V” stand for any VCOV other than ${\textbf {I}}$ in *GL* or *GY*, and “*G*” when ${\textbf {G}} \sim (\varvec{0}, \sigma ^2_G {\textbf {G}}_{{\textbf {M}}})$ instead of ${\textbf {G}} \sim (\varvec{0}, \sigma ^2_G {\textbf {I}})$

$^{\textrm{c}}$RGG is directly estimated from $\beta _g r_i$, where $\beta _g$ is a heterogeneous regression coefficient of the genetic trend for experimental lines and check cultivars, and $r_i$ is the first year of testing. The same holds for $\beta _t t_k$, where $t_k$ is a continuous covariate for the calendar year

$^{\textrm{d}}$ $C_p$ is used to designate control population, which has two factors (check varieties and experimental lines)

$^{\textrm{e}}$ $\hat{d}$ represents the correction term presented in Supplementary Material

#### Estimation models applied to simulated BT3, PYT and URT

All experimental lines in BT3, PYT, and URT were included in the analyses, but RGG was estimated only from the breeding lines (Table 7). The indirect RGG estimate was computed with modified versions of Models (4) and (5), where $\hat{g}_{i_{(s)}}$ and $\varvec{\kappa }_{(s)}$ are restricted to breeding lines, and $r_{i_{(s)}}$ was replaced by $w_{i_{(s)}}$.

Models with data from BT3 provide crossing information and include variables for types of general ($P_i$) and specific ($F_{ii'}$) combining abilities (Table 7). We refer to this information as “types of combining abilities” because data from PYT and URT are selected lines derived from progeny of crosses. The selection process in MET (BT1 $\rightarrow$ BT2 $\rightarrow$ BT3 $\rightarrow$ PYT) results in the best $\sim$0.4 percent of self-pollinated experimental lines in advanced trials. Thus, we can actually estimate the combining abilities of breeding lines to produce the best $\sim$ 0.4 percent of lines created for the next cycle of line development.

Six models were applied to these data sets. Model E10 treats the variable values in $P_i$ as random effects (Möhring et al. 2011) and Model E11 as fixed effects. Model E10G included ${\textbf {G}}_{{\textbf {M}}}$. Shrunken or GEBVs were then calculated as $2 \times \hat{P}_i$ (Isik et al. 2017) and used to indirectly estimate RGG. For Models E10 and E10G, estimates of RGG were obtained using Model 5 ($\varvec{\kappa }_{(s)}$). Model E12 provided direct estimates of RGG by replacing the main effects of lines used for crossing with $\beta _{f_{(s)}} w_{i_{(s)}}$, where $\beta _{f_{(s)}}$ is the estimated slope, and $w_{i_{(s)}}$ the year the *i*th breeding line was used for crossing. Note only one term $\beta _{f_{(s)}} w_{i_{(s)}}$ was included in this model because breeding lines were not used for crosses in multiple years, so that $w_{i_{(s)}}$ is equivalent for $P_i$ and $P_{i'}$. Models E13 and E14 are similar to E7 and E8 (Table 6), respectively, where the check cultivars were used to predict environmental effects in the first step.

**Table 7:**
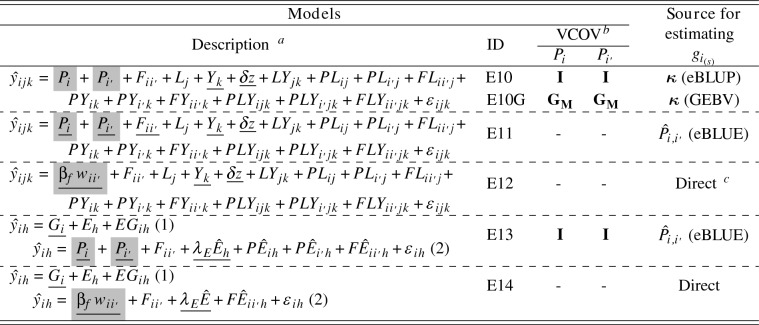
Models applied to simulated BT3, PYT and URT. These models include information from breeding lines used in crossing. Fixed effects are underlined, and the RGG was estimated with model terms highlighted in gray

$^{\textrm{a}}$ $P_{i}$ and $P_{i'}$ are the general combining ability effects of experimental lines used in crossing blocks, and $F_{ii'}$ stands for specific combining ability effects. Model terms *G*, *L*, *Y*, and *E*, are the main effects of genotypes (experimental lines), locations, years, and environments ($h = 1, \dots , H$), respectively. The response variables $\hat{y}_{ijk}$ and $\hat{y}_{ih}$ are the vectors of eBLUE values of genotypic means. The covariates *z* and $t_k$, with regression coefficients $\delta$ and $\beta _t$, are the mapping variables of non-genetic trend linked to the frequency of locations (Eq. 2), and a continuous covariate for the calendar year, respectively. The covariate $\hat{E}$ is the predicted eBLUP values of *E*. The residual $\epsilon _{ijk}$ ($\epsilon _{ih}$) was included when stage-wise models were not used. The numbers in parentheses (1, 2) in the model’s description represent steps to fit the models. Models in step one (1) were adjusted only with check cultivars

$^{\textrm{b}}$VCOV modeled for P$_i$ and P$_i'$ were identity (**I**) and the additive genomic relationship matrix (${\textbf {G}}_{{\textbf {M}}}$)

$^{\textrm{c}}$ RGG is directly estimated from $\beta _f w_{ii'}$, where $\beta _f$ is the regression coefficient of the genetic trend for parental lines, and $w_{ii'}$ is the year parents were used in crossing blocks (i.e., the F$_1$ was generated)

### Statistical comparisons of evaluated models

#### Covariance modeling

Models E0, E1, $\dots$, and E6, assumed independent GEI effects with homogeneous variance, whereas their counterparts E0V, E1V, $\dots$, and E6V, assumed correlated effects with heterogeneous variances or covariances (Table 6). We hypothesize there is no substantial difference in estimating RGG due to variance-covariance (VCOV) modeling. We formally tested our hypothesis by applying an analysis of variance (ANOVA) to the estimated RGG values as a function of the simulated breeding program (i.e., simulation run) and evaluated models (Supplementary Material Figure A6). The average RGG (i.e., the slope $\beta _{R}$) of each model ($\beta _{R_{E0}}$, $\beta _{R_{E0V}}$, $\dots$, $\beta _{R_{E6}}$, $\beta _{R_{E6V}}$) was then computed, and the pairwise differences ($\beta _{R_{E0}} - \beta _{R_{E0V}}$, $\beta _{R_{E1}} - \beta _{R_{E1V}}$, $\dots$, $\beta _{R_{E6}} - \beta _{R_{E6V}}$) were tested with the Tukey method with $\alpha = 0.05$ adjusted for multiplicity using the R package emmeans (Lenth 2022, functions *lstrends* and *pairs*). The same procedure was applied to compare independent versus correlated genotypic effects, and direct versus indirect estimates of RGG.

#### Bias and linearity

Because $\hat{\beta }_{R_{(s)}}$ (Model 4) is an estimator of $\beta _{T_{(s)}}$ (Model 3, “true” simulated RGG), the estimation bias or error is defined as $\xi _{(s)} = \hat{\beta }_{R_{(s)}}-\beta _{T_{(s)}}$. Models were evaluated based on the average ($\bar{\xi }$) and relative ($\xi ^{\textrm{rel}}_{(s)}$) bias across simulation runs, as well as on the root mean squared error (${\textit{RMSE}}$), computed as follows:

6 $$\begin{aligned} \bar{\xi }&= \frac{\sum _{s=1}^{S}\xi _{(s)}}{S} \end{aligned}$$

7 $$\begin{aligned} \xi ^{\textrm{rel}}_{(s)}&= \frac{\sum _{s=1}^{S} \xi _{(s)} / \beta _{T_{(s)}}}{S} \times 100 \end{aligned}$$

8 $$\begin{aligned} {\text {RMSE}}&= \sqrt{\frac{\sum _{s=1}^{S}\xi ^2_{(s)}}{S}} \end{aligned}$$

The same metrics were calculated for $\hat{\beta }_{g_{(s)}}$, the direct estimator of RGG. Most models used to obtain estimates of RGG did not use information from experimental lines used in crosses (Table 6). All experimental lines selected for recycling based on the results of BT3 + BT2 + BT1 were included in the PYT (Fig. 1), however, not all experimental lines in PYT were selected as breeding lines. Thus, when pedigree information is not available, the estimated RGG from advanced PYT and URT trials is a mixture of experimental lines used for crossing, and experimental lines that simply advanced from BT3 to PYT based on their yearly performance. Thus, the expected bias of estimating RGG from advanced trials without pedigree information is the difference between true simulated RGG ($\beta _{T_{(s)}}$) and the true trend from MET ($\beta ^{\textrm{true}}_{R_{(s)}}$). The regression slope $\beta ^{\textrm{true}}_{R_{(s)}}$ was calculated from Model 4 by replacing $\hat{g}_{i_{(s)}}$ by true simulated genetic values (i.e., no error nor GEI). Results for expected bias are reported using the term “Expected.” Note the expected bias is zero for Models E10, $\dots$, E14 (Table 7), where the pedigree is known and the RGG was estimated from breeding lines.

In addition to bias, a metric for linearity was assessed. The linearity metric is based on the premise that if there is continuous genetic progress due to the selection of a (additive) quantitative trait and constant genetic variance, then a linear trend between years of line development and genotypic means for MET should be evident. For indirect models where marginal genotypic (line) effects are estimated/predicted, we formally tested for linearity with the sieve-bootstrap version of the Student’s *t*-test (Lyubchich and Gel 2022; Noguchi et al. 2011). The null hypothesis of no trend versus the alternative hypothesis of a linear trend was assessed on $\alpha = 0.05$ with Bonferroni correction, and the proportion of statistically significant trends across simulation runs was reported. For direct models, we report the distribution of the two-tailed *p*-values from the estimated z-ratio (point estimate/standard error) of the slope ($\beta _g, \beta _t, \beta _f$) as an indication of its significance. This distribution was compared to the random sample simulation model B2-R (Table 2).

### Estimation of RGG from the empirical soybean data

Models that demonstrated the least bias in analyses of simulated data were used to estimate RGG from the empirical data. This data refers to historical soybean seed yield records of maturity groups II and III evaluated in PYT and URT between 1989 to 2019. The data set contains 39,006 data points, 4257 experimental genotypes derived from multiple public breeding programs, and 591 observed environments located in the United States and Canada. The dataset can be obtained from the R package SoyURT. Refer to Krause et al. (2023) for more details.

## Results

### Simulation overview

**Fig. 3:**
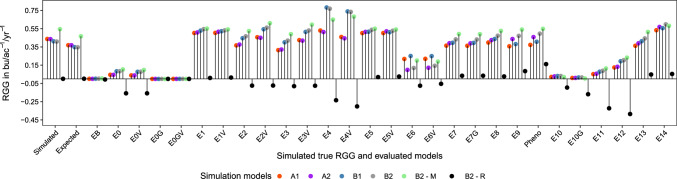
Average values for true RGG ($\hat{\beta }_{T}$), expected RGG ($\hat{\beta }^{\textrm{true}}_{R}$), and estimated RGG ($\hat{\beta }_{R}, \hat{\beta }_g, \hat{\beta }_t, \hat{\beta }_f$) for the evaluated models (*x*-axis)

In total, $\sim 1.03$ trillion data points were simulated in MET across 1350 breeding programs. The number of different sampled locations ranged from 28 to 41. The sample sizes (i.e., the number of experimental lines excluding checks) ranged from 5000–18,723 for BT1; 500–1872 for BT2; 100–374 for BT3; 21–75 for PYT; and 4–15 for URT. Estimated broad sense heritabilities on an entry mean basis (Cullis et al. 2006) for individual trials within breeding stages had larger values for the more advanced trials (Supplementary Material Figure A7 and Supplementary Material Figure A8). The average true simulated RGG in bu/ac$^{-1}$/yr$^{-1}$ was 0.44 for A1 and A2, 0.41 for B1 and B2, 0.55 for B2-M, and zero for B2-R (Fig. 3).

### Covariance modeling

Including covariance models for GEI significantly affected contrasts of estimated RGG values. Three contrasts were statistically significant when a random sample of breeding lines was used to create a new cycle of breeding (B2-R). The inclusion of the genomic relationship matrix (${\textbf {G}}_{{\textbf {M}}}$) was statistically significant for the contrast E10–E10G in B2-R, and E0–E0G was significant for all B simulation models. The direct versus indirect estimation of RGG resulted in significant differences between estimated RGG values for most simulation models; the primary exception was that the contrast between Models E7 and E8 was not large for any of the simulation models (Table 8).

**Table 8:** Adjusted Turkey *p*-values for the contrasts between variance-covariance modeling (VCOV) and direct versus indirect (DI) estimation of RGG

Contrast	Simulation models						Type
	A1	A2	B1	B2	B2-M	B2-R	
E0–E0V	1.00	1.00	1.00	1.00	1.00	1.00	VCOV
E0–E0G	0.48	0.67	< 0.01	< 0.01	< 0.01	< 0.01	VCOV
E1–E1V	1.00	1.00	1.00	1.00	1.00	1.00	VCOV
E2–E2V	< 0.01	< 0.01	< 0.01	< 0.01	< 0.01	1.00	VCOV
E3–E3V	< 0.01	< 0.01	< 0.01	< 0.01	< 0.01	1.00	VCOV
E4–E4V	< 0.01	< 0.01	0.71	0.98	0.99	0.01	VCOV
E5–E5V	1.00	1.00	1.00	1.00	1.00	1.00	VCOV
E6–E6V	1.00	1.00	1.00	1.00	1.00	1.00	VCOV
E7–E7G	1.00	1.00	1.00	1.00	1.00	1.00	VCOV
E10–E10G	1.00	1.00	1.00	1.00	1.00	< 0.01	VCOV
E1–E2	0.00	0.00	0.00	0.01	1.00	0.00	DI
E1V–E2V	0.35	0.03	1.00	1.00	0.03	0.00	DI
E7–E8	0.94	0.97	0.80	0.92	0.98	1.00	DI
E11–E12	0.00	0.00	0.00	0.00	0.00	0.00	DI
E13–E14	0.00	0.00	0.00	0.00	0.04	0.00	DI

### Relative bias and overall performance

On average, RGG values from analytic Models EB, E0G, E0GV, and E10G were estimated at zero (Fig. 3). The simulation model B2-R was designed to not have a positive RGG value due to the random sampling of genotypes (i.e., no selection). Then, when the estimated RGG values from the tested models were compared in B2-R, a large variation in the results complicated the comparison of the models. For example, if the true RGG in a simulation run in B2-R was 0.0001 bu/ac$^{-1}$, an estimated value of 0.01 from any model would be 100 times bigger than the simulated one. Thus, results from these models and from B2-R will not be included in the reported summary statistics below. Raw estimates of RGG are available in (Supplementary Material Figure A9–Supplementary Material Figure A14), as well as the estimated bias without standardization in (Supplementary Material Figure A15).

**Fig. 4:**
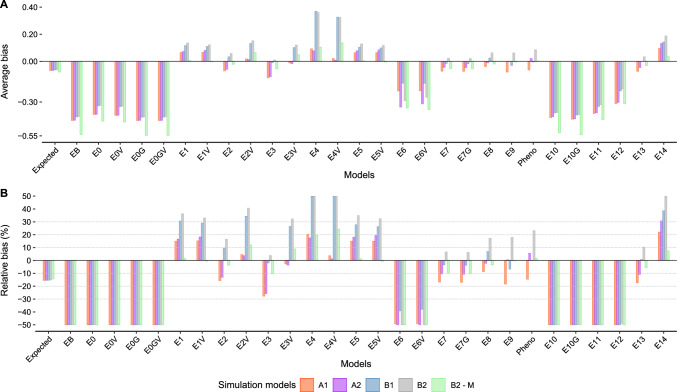
**A** Average bias in bu/ac$^{-1}$/yr$^{-1}$ and **B** relative bias across simulations. The *y*-axis in **B** was limited to $\pm\, 50$% to enhance visualization

**Fig. 5:**
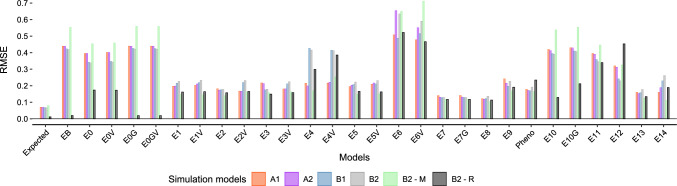
Root mean squared error (RMSE)

All models demonstrated some degree of bias. On average, a smaller bias was observed for simulation models with complex GEI effects. The expected relative bias for models without information from breeding lines had an average value of $-$15.21% (Figs. 4 and Supplementary Material Figure A16). Models with information from breeding lines are expected to have no bias, but did not outperform (i.e., less biased) models that only considered advanced trials (Figs. 3, 4 and 5). Including ${\textbf {G}}_{{\textbf {M}}}$ to account for correlated genetic effects did not improve the accuracy of the estimated RGG values. Across simulation runs, the average value of the diagonal values of ${\textbf {G}}_{{\textbf {M}}}$ was 1.91, and zero for the off-diagonal. Within years, these values were 1.91 and 0.49, respectively (data not shown).

On average, 15 models presented less than $\pm\, 5$% relative bias for at least one simulation model. Models E2, E7, E7G, E8, and E13 had less than $\pm\, 18$% relative bias across all simulation models (Fig. 4). Directional relative bias (i.e., under or overestimating true RGG on average) was observed for Models E1, E2V, E4, E4V, E5, E6, E6V, E10, E11, E12, and E14, and very similarly for Models E1V, E3, E3V, and E5V. Across simulation models and excluding very few negative estimates (Supplementary Material Table A1), the range of estimated RGG values from Models E1, E2V, and E7, contained the true simulated RGG in 58% of the simulations (Supplementary Material Figure A17). For these models, the relative bias ranged from $-$16.82% to 40.73%, which represents biases of $\pm\, 7.41$ kg/ha$^{-1}$/yr$^{-1}$ or $\pm\, 0.11$ bu/ac$^{-1}$/yr$^{-1}$.

Estimates of RGG using raw phenotypic data (“Pheno”) resulted in relative bias across simulations from $-$14.64% to 23.23% (Fig. 4), with a similar $\textit{RMSE}$ as other analytic models (Fig. 5). However, in B2-R, where there was no true simulated RGG, using raw phenotypic data resulted on average in an estimated RGG of 0.16 bu/ac$^{-1}$/yr$^{-1}$ (Fig. 3). Simulation models A2, B2, B2-M, and B2-R, included a positive non-genetic trend. Although in this work we are not investigating the estimation of the rate of non-genetic gain (i.e., we were trying to isolate it from RGG), by comparing the relative bias in scenarios A1 versus A2, and B1 versus B2, it is evident some analytic models successfully isolated it (Fig. 4).

### Linearity

**Fig. 6:**
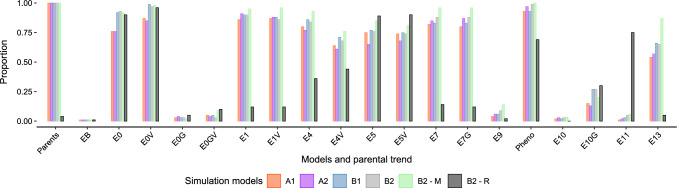
The proportion of statistically significant linear trends for models that indirectly estimated RGG

**Fig. 7:**
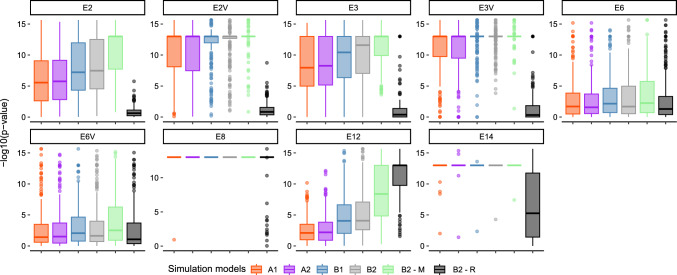
Distribution of the $-\log 10$ (*p*-values) from the z-ratio statistic of direct RGG estimation

For Models E0V, E1, E1V, E7, and E7G, the proportion of statistically significant linearity ranged from 0.80 to 0.99, with an average value of 0.90. As expected, true simulated genetic values from breeding lines were always statistically significant. In B2-R, Models E0, E0V, E4, E4V, E5, E5V, E10G, and E11, presented an average value of 0.63, similar to when raw phenotypic values were considered (Fig. 6). For models in which RGG was directly estimated, the distribution of the *p*-values from the z-ratio statistic showed the linearity metric could be used to indicate there was RGG, except for Models E6, E6V, and E12, where results were similar to the simulation model B2-R (Fig. 7).

### Estimates of RGG from empirical data

According to the estimates of bias, directional bias, RMSE, and linearity from the evaluated models using simulated data, we used Models E1, E2V, and E7 to estimate RGG for the empirical data. For Model E2V, several covariance structures were evaluated, and the best-fit model was selected (Supplementary Material Table A2). The point estimates of RGG ranged from 0.27 to 0.59 bu/ac$^{-1}$/yr$^{-1}$. For Models E1 and E7, the *p*-values from the linearity test were statistically significant, as well as for the z-ratio of the estimated slope $\beta _g$ (i.e., the direct estimate of RGG) from Model E2V (Table 9). Therefore, although imprecise, there is strong evidence that RGG from public soybean breeding programs has been positive for maturity groups II and III in the period from 1989 to 2019.

**Table 9:** Estimated RGG with 95% confidence interval (bu/ac$^{-1}$/yr$^{-1}$) and linearity for the empirical soybean dataset

Model	RGG	Linearity
E1	0.59 (0.58, 0.61)	< 0.01
E2V	0.27 (0.20, 0.35)	< 0.01
E7	0.42 (0.40, 0.43)	< 0.01

## Discussion

Since the beginning of selection for quantitative traits, the development of analytical methods to estimate RGG has been pursued by animal and plant breeders (Eberhart 1964; Garrick 2010; Rizzo et al. 2022). The motivations include the need for an accurate metric that can be used to assess return on investment and can be used to evaluate proposed novel breeding strategies (Rutkoski 2019a, b). The challenge for the development of any statistical metric is to clearly define the inference space. For line development programs, we propose that the inference space consists of lines used in crossing, i.e., a breeding population, consisting of nearly homozygous genotypes that are used as parents in crossing blocks. If experimental lines in advanced MET have accumulated favorable alleles from selection, i.e., have higher average breeding values than their parents from the previous cycle of development, then genetic trends across years of MET are a function of the breeding values of the lines used for crosses, and therefore herein we interpret the comparison across time as an estimate of RGG from all possible germplasm that is adapted to the variable and changing conditions of MET. This connection between accumulation of favorable alleles and expression of the phenotype in advance MET recognizes that RGG in cultivar development programs can include a culling process executed across multiple environments, and migration and drift, as contributors to the concept of genetic gain (Kempthorne 1957; Falconer 1960).

Similar to breeding using recurrent population improvement methods (Jenkins 1940), cultivar development consists of evaluation, selection, and reproduction, although there are relevant distinctions. There is no single cycle zero from which all genotypes are recurrently derived. Indeed, within both public and proprietary plant breeding programs, there are many geographically distributed line development programs that began with unique sets of founders. Further, each line development program has its own set of objectives and local environments, thus resulting in breeding islands, although lines are exchanged and evaluated in common MET. Consequently, genetic gains can be due not only to selection, but also to migration and drift. In this work, we simulated a broad range of environments in a closed system, i.e., there was no exchange or introgression of germplasm from external sources. Future simulations should consider the role of breeding “islands” and the exchange of breeding lines to more accurately reflect the underlying mechanisms responsible for the historical records (Ramasubramanian and Beavis 2021). Fortunately, the concept of RGG using our defined inference space still applies to a more open system as long as adjusted genotypic means (i.e., eBLUE values) for migrants that become breeding lines are included in the estimation of RGG.

Distinguishing RGG from genotypic and/or commercial gain will help avoid misleading interpretations. Genotypic gain, not genetic gain, is RGG plus the expression of non-additive genetic effects such as epistatic (Hansen and Wagner 2001; Pavlicev et al. 2010) and dominance deviations. Hence, for a trait not only controlled by additive effects (e.g., Garcia et al. 2008), the genotypic gain is expected to be higher than the RGG. Commercial gain is the genotypic gain delivered in the farmer’s field. Examples of commercial gain are estimates from era trials (e.g., Bruce et al. 2019; Cooper et al. 2020) where only widely grown cultivars are used to represent a specified era. The essential distinction between RGG, genotypic, and commercial gain, is that only RGG is relevant to genetic improvement by the breeding project.

Another metric called “yield gain,” ‘yield advances,” or simply “genetic trend”, has been used to quantify yield increase of staple crops worldwide (Grassini et al. 2013; Prasanna et al. 2022) or in specific countries (Fischer et al. 2022; Guo et al. 2022; Rizzo et al. 2022). For example, Rizzo et al. (2022) collected maize field-trial data over 14 years (2005–2018) from the state of Nebraska (USA), and concluded that climate and agronomy represented 87% of the yield gains in high-yield irrigated environments, leaving 13% for the genetic contribution. The 13% genetic contribution can be interpreted as an unweighted average of the commercial gain. It is unweighted in the sense that individual commercial programs breeding for Nebraska have a specific contribution to the reported gain, given each program has released a number of hybrids with an average lifespan of three years according to their market share. For that reason, the yearly yield gain is a composition of commercial gain within and across breeding programs. Consequently, careful consideration should be given when linking reported genetic trends with RGG.

The main emphasis of this study was to estimate RGG from advanced MET. We choose LMM for estimators because they are commonly used in data analysis of MET (Isik et al. 2017; Dias et al. 2018; Krause et al. 2020) and are well-known in the plant and animal breeding communities. The underlying distributional assumptions of LMM are that random effects have an expected value of zero, and are realizations of independent and multivariate Gaussian distributions with positive-definite variance matrices (Gumedze and Dunne 2011). By assuming the random effects have an expected value of zero, both the average (Isik et al. 2017) and sum (Searle 1997) of predicted values for the random factor are zero. This analytic constraint, however, does not assure the eBLUP value of a newly developed genotype will be numerically higher than that of an older genotype when historical MET data is analyzed using $G\times L\times Y$ models (Supplementary Material Figure A21). Thus, our benchmark Model EB did not provide positive values for RGG.

An alternative parametrization for the $G\times L\times Y$ benchmark model was computed with the cumulative sum of the average eBLUP values of all genotypes according to the year of first testing (e.g., the PYT for the empirical and simulated data). This strategy is connected to the expected gain from selection when all genotypes that are under selection are analyzed together (i.e., the same model). In this case, the expected/predicted gain can be directly calculated with the average of the eBLUP values from selected individuals (Falconer and Mackay 1996; Walsh and Lynch 2018). The rationale is that, when likelihood-based estimators of variance components are used (e.g., REML), the regularization parameter lambda in the BLUP predictor is usually the ratio of residual and random term variance estimates. Hence, for the main effects of genotypes, the shrinkage of the genetic term is inversely proportional to the heritability/repeatability of the trait (Xavier et al. 2016). The practical application of $\varvec{\kappa }$ relies on the assumption that the observed experimental genotypes in MET are a realization of a multivariate random variable with covariance depending on genetic relationships. This assumption does not require reference to a specific base population with idealized properties (Piepho et al. 2008). Furthermore, in parallel to recurrent selection, the first cycle is arbitrarily set by the first available year in the MET data, thus representing the initial value of $\varvec{\kappa }$.

Another alternative for the benchmark Model EB was including a variable for environments (location–years combination). The idea underlying this approach is from Diers et al. (2018) and Montes et al. (2022). These authors analyzed phenotypic data from a soybean nested association panel using stage-wise models. In the first-stage model, the eBLUP values of incomplete blocks within locations were predicted using a unique identifier in the dataset, and in the second stage, the eBLUP values for incomplete blocks were used as a fixed effect covariate. We modified their first-stage model to obtain the eBLUP values of environmental effects ($\hat{E}$) based on evaluations of check cultivars, and subsequently considered the values as a fixed effect covariate to represent both *E* and GEI effects. We hypothesized that if the non-genetic effects can be successfully captured by check cultivars in the first stage (Supplementary Material Figure A22), then unbiased estimates of genetic effects could be obtained from the second-stage model.

While this second modeling strategy successfully captured RGG and GEI effects, it significantly inflated the estimates of genotypic variance, and consequently, the estimates of heritability (Supplementary Material Figure A21 and Supplementary Material Figure A23). For example, estimates of genotypic variance and heritability using empirical soybean MET data changed, respectively, from 6.30 (bu/ac)$^{2}$ and 0.47, in the benchmark Model EB, to 31.11 (bu/ac)$^{2}$ and 0.94 with Model E7. A similar outcome can be achieved by dropping the interaction terms *GL*, *GY*, and *GLY* from the benchmark model, where the estimated genotypic variance changed from 6.30 (bu/ac)$^{2}$ to 27.01 (bu/ac)$^{2}$. These results suggest the genotypic variance in Models E7 and E7G were inflated by the *GL*, *GY*, and *GLY* variances associated with the experimental genotypes. Thus, while checks account for variability among environments of MET, the GEI effects from experimental genotypes are still confounded with the genotypic effects.

The aforementioned inflation due to GEI applies to other mixed models used for analyzing data from MET. For example, the “EBV” model from Rutkoski (2019b) only accounted for genotypic main effects and successfully captured RGG with a biased (inflated) genotypic variance. In this case, RGG estimates from eBLUP values [${\textbf {G}} \sim N(\varvec{0}, {\textbf {I}}\sigma ^2_G)$], estimated breeding values [${\textbf {G}} \sim N(\varvec{0}, {\textbf {A}}\sigma ^2_G)$], or genomic estimated breeding values [${\textbf {G}} \sim N(\varvec{0}, {\textbf {G}}_{{\textbf {M}}}\sigma ^2_G)$], will not produce large differences, as shown in Models E7 and E7G. Nonetheless, for annual and breeding line selections, it is well-known that under normality, equal variance, and independence, BLUP minimizes the mean squared error of predicted values (Robinson 1991; Piepho et al. 2008) and hence maximizes the correlation between true and predicted genotypic values (Henderson 1963; Searle 1974; Searle et al. 1992). Thus, although RGG from MET cannot be directly estimated from eBLUP values of the $G\times L\times Y$ models using large historical data, the use of BLUP is likely to increase gains from selection if data is correctly modeled (Smith et al. 2005; Hartung et al. 2023).

Every model assessed to estimate RGG in the simulations demonstrated some degree of bias. For example, the average bias was similar between simulation models A1 and A2 (simple GEI effects), and in B1 and B2 (complex GEI effects), but were relatively different between the A and B simulations. Including a positive, cumulative, non-genetic gain did not result in major bias when comparing simulation models A1 versus A2, and B1 versus B2. Ideally, RGG should be estimated with models with small values for root mean squared error (Supplementary Material Figure A18–Supplementary Material Figure A20). Even though all evaluated estimation models produced biased results, note that for some models the bias was consistently in the same negative/positive direction relative to the “true” simulated RGG values, indicating that the estimated RGG values underestimate/overestimated on average the true RGG. Such directional bias can be seen as insurance that the estimated RGG from empirical datasets is likely under or overestimated, depending on the model of choice.

The simulator was constructed to mimic public soybean breeding programs responsible for maturity zones II and III in the USA. These programs evaluate nearly homozygous experimental lines in replicated field trials for four to five years (Fig. 1), but the available empirical data consist of only the last two years of MET. We assumed for simulation that breeding lines were selected in BT3 after three years of trials, and then were used in the crossing nursery as well as advanced to a regional PYT. However, the PYT stage could include lines that were not used in the crossing nursery. There could be lines from other line development programs or there could be experimental lines that might be considered for release as purelines, but not for crossing; not all lines considered for release as purelines also produce high-yielding progeny. Consequently, estimates of RGG based on data from only PYT and URT, with no information about which lines are used for crossing, represent a biased sample relative to the breeding lines that are used to create the next cycle of segregating progeny. Our results show that the average bias was $-$15.21% across all simulation models, indicating that, on average, the samples of lines included in the PYT and URT underestimate the true RGG. In addition, given only 20–30 experimental lines were used in crossing nurseries each year, genetic drift associated with different intensities of selection throughout the pipeline might also have contributed to the observed bias (Falconer and Mackay 1996; Vaughn and Li 2016; Walsh and Lynch 2018).

Based on theory, larger samples will asymptotically produce more accurate and precise estimates. We focused on estimating RGG from 30 years of advanced MET and could not obtain an unbiased, robust estimator. Results considering only 10 years of data revealed a much larger bias and root mean squared error, as also observed by Rutkoski (2019b). The simulated breeding programs in this work and from Rutkoski (2019b) assumed small breeding projects relative to proprietary ones. Proprietary breeding programs have a much larger network of trials, which could easily represent a threefold to sixfold increase in the amount of data. In addition, their field trials comprise lines and checks that belong to narrow (0.1) relative maturity (RM) groups. For example, Byrum et al. (2017) developed an environmental index representing the non-genetic component of seed yield from data collected on thousands of check varieties, each representing every 0.1 RM group and grown in hundreds of MET per RM every year for a ten year period. Yield values for each experimental line grown in the same trials as the check varieties were adjusted by subtracting the environmental index. The primary feature of this Genetic Gain Performance (GGP) metric is its reliance on data from a large number of check varieties, grown in a large number of trials and environments, so that the environmental index could be accurately estimated using the Expectation-Maximization (EM) algorithm (Dempster et al. 1977). Thus, a question for further investigation is to determine the optimal (or minimum) number of trials, locations, and years from advanced MET needed to obtain unbiased estimates of RGG using LMM. Also, strategies to increase the trial’s accuracy (e.g., reliability) such as modeling field spatial patterns can be considered to increase the accuracy of the estimated genotypic means (Gilmour et al. 1997; Borges da Silva et al. 2021).

We used simulated data from two or three years of MET, but excluded the simulated data from local breeder trials. We excluded these because empirical data from local breeder trials historically have not been published nor recorded in accessible databases. However, it is well known that a pattern of missing data also can introduce bias in trend (Hartung et al. 2023) and REML variance estimation (Piepho and Mohring 2006; Aguate et al. 2019; Hartung and Piepho 2021). Further research is needed to investigate if this exclusion introduced bias in estimates of RGG. Furthermore, simulated breeding lines were selected in BT3 with BLUP models that consider all available data (BT1, BT2, BT3). It is worthwhile investigating if these predicted values could lead to less biased estimates of RGG. This approach would also be informative regarding the predicted genetic gain as previously discussed. In addition, early generations can also be used to estimate RGG (Cowling et al. 2023).

Computing RGG from raw phenotypes, without statistical modeling, can indicate positive RGG when no genetic gain was actually delivered. One might argue that when the data is balanced the arithmetic average and (generalized) least-squares yield numerically equivalent estimates. The first issue is that data from MET are rarely balanced within years, and largely unbalanced across years. Thus, RGG estimates from raw phenotypes are completely confounded with non-genetic effects. As emphasized by Hartung et al. (2023), careful consideration should be given in selecting the best-fit, proper, model for the dataset being analyzed. Great attention should be given to evaluating covariance modeling for non-genetic effects given it played an important role for most models. Metrics such as the Akaike and Bayesian information criteria (Akaike 1974; Schwarz 1978), as well as the proportion of genetic variance explained by FA models (Smith et al. 2015), should always be considered.

Both direct and indirect estimators of RGG provided useful information. Check Piepho et al. (2014) for the theoretical development of direct estimation. Including additive genomic relationships to account for correlated genetic effects did not improve the RGG estimates. This result likely occurs due to genotypes exhibiting little to no relationship across years of MET. Diallel-based models were also evaluated assuming pedigree was available, so RGG was estimated directly from breeding lines. These models did not outperform models that only considered advanced trials, and hence there is no clear advantage in considering this modeling approach. When pedigree is available, an alternative to compute RGG would be to use breeding lines *per se* performance. We did not test for this approach, but results from Rutkoski (2019b) showed unbiased estimates could not be obtained.

Overall, the best-performing models were E1, E2V, and E7. Since none of these models yielded unbiased estimates of RGG, the most suitable strategy would be to account for the range of the estimated values. This would increase the likelihood of capturing the true RGG. Using this strategy, we report estimates of RGG that ranged from 18.12 to 39.60 kg/ha$^{-1}$/yr$^{-1}$ (0.27 to 0.59 bu/ac$^{-1}$/yr$^{-1}$) for 31 years of empirical soybean MET, considering all sampled environments from the target population of environments. This result further assumes that there is only one set of BTs from a single cultivar development project. It should be pointed out that there are actually multiple variety development projects working in maturity zones II and III, so there are multiple sets of BTs and further work using island models (Ramasubramanian and Beavis 2021) are needed to provide better interpretation of results (including maintenance of genetic variability) from MET. Lastly, if the goal is to determine if there is evidence for RGG, regardless of bias, our linearity measure based on the simulation results is useful. Further investigation is needed if RGG is expected to be nonlinear due to changes in the genetic variance, heritability, and non-additive effects (Eberhart 1964; Bulmer 1971; Ramasubramanian and Beavis 2021).

## Conclusion

We evaluated several LMM to estimate RGG using advanced MET. We approach the research question by simulation and propose a careful characterization of the inference space for RGG that considers the intricate nature of cultivar development programs while remaining consistent with the original concept of genetic gain. Our results suggest it is not possible to accurately estimate RGG using data from two years of MET, such as are available in the public soybean breeding programs in the USA. Consequently, the evaluated estimators should not be used to compare breeding programs or quantify the relative efficiencies of proposed breeding systems. If the goal is only to determine whether there was RGG, the linearity metric is useful. Therefore, as also concluded by Rutkoski (2019b), there were no unbiased estimates from LMM to estimate RGG using limited samples from large complex interactions among genetic and non-genetic conditions. Lastly, in addition to the practical and theoretical results applied to soybean genetic improvement, the analyses performed in this study can be applied to quantitative traits evaluated in any diploid crop undergoing phenotypic evaluations in MET.

### Supplementary Information

Below is the link to the electronic supplementary material.

Supplementary file1 (PDF 5953 kb)

## Data Availability

The simulator and evaluated models are publicly available on GitHub (https://github.com/mdkrause/RGG). The soybean empirical data is available in the R package SoyURT (https://github.com/mdkrause/SoyURT).
