# Supplementary material for: Models to estimate genetic gain of soybean seed yield from annual multi-environment field trials
Source: Theor Appl Genet. 2023 Nov 21;136(12):252. doi: 10.1007/s00122-023-04470-3 (PMC10663270; doi:10.1007/s00122-023-04470-3)
Supplement: Supplementary file 1 — Supplementary file1 (PDF 5953 kb) [file 122_2023_4470_MOESM1_ESM.pdf]

Models to Estimate Genetic Gain of Soybean Seed Yield from Annual Multi-Environment Field Trials

https://doi.org/10.1007/s00122-023-04470-3

Supplementary Information

Throughout the Supplementary Information, the labels “Minimum GEI (B2)”, “Random selection (B2)”, and ”Scenarios”, are used as synonyms of B2-M, and B2-R, and simulation models, respectively.

A - Additional figures

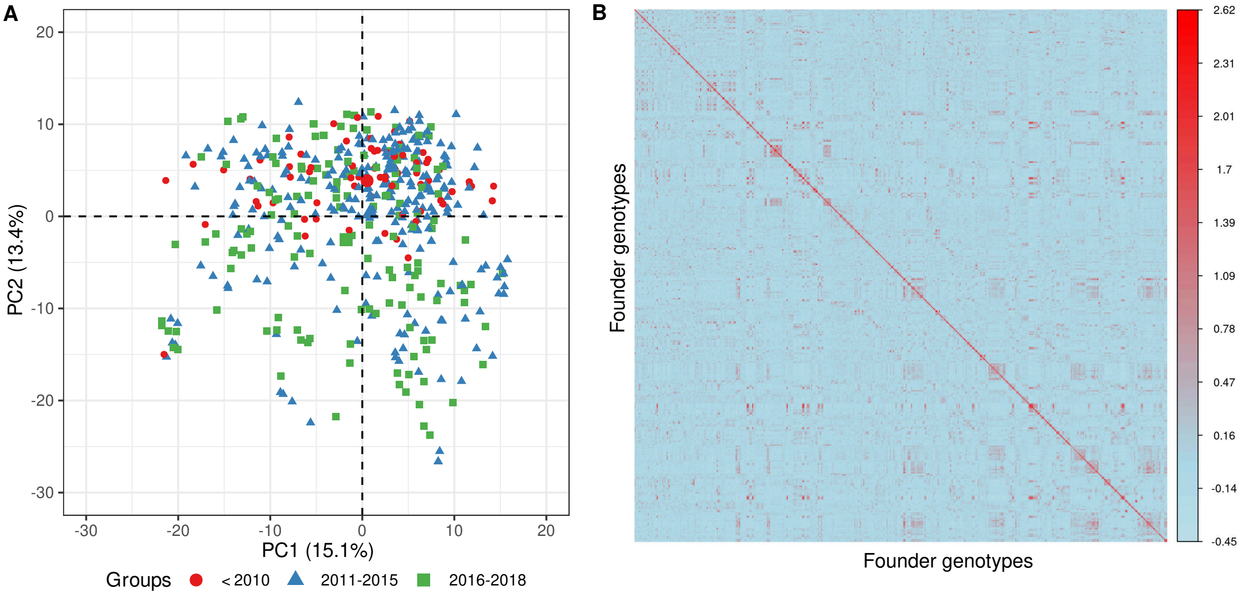

Figure A1: Principal component analysis of the additive genomic relationship matrix ($\mathbf{G_M}$) estimated from the population of experimental genotypes used as founders in the simulation (A), and a heatmap of the $\mathbf{G_M}$ matrix (B).

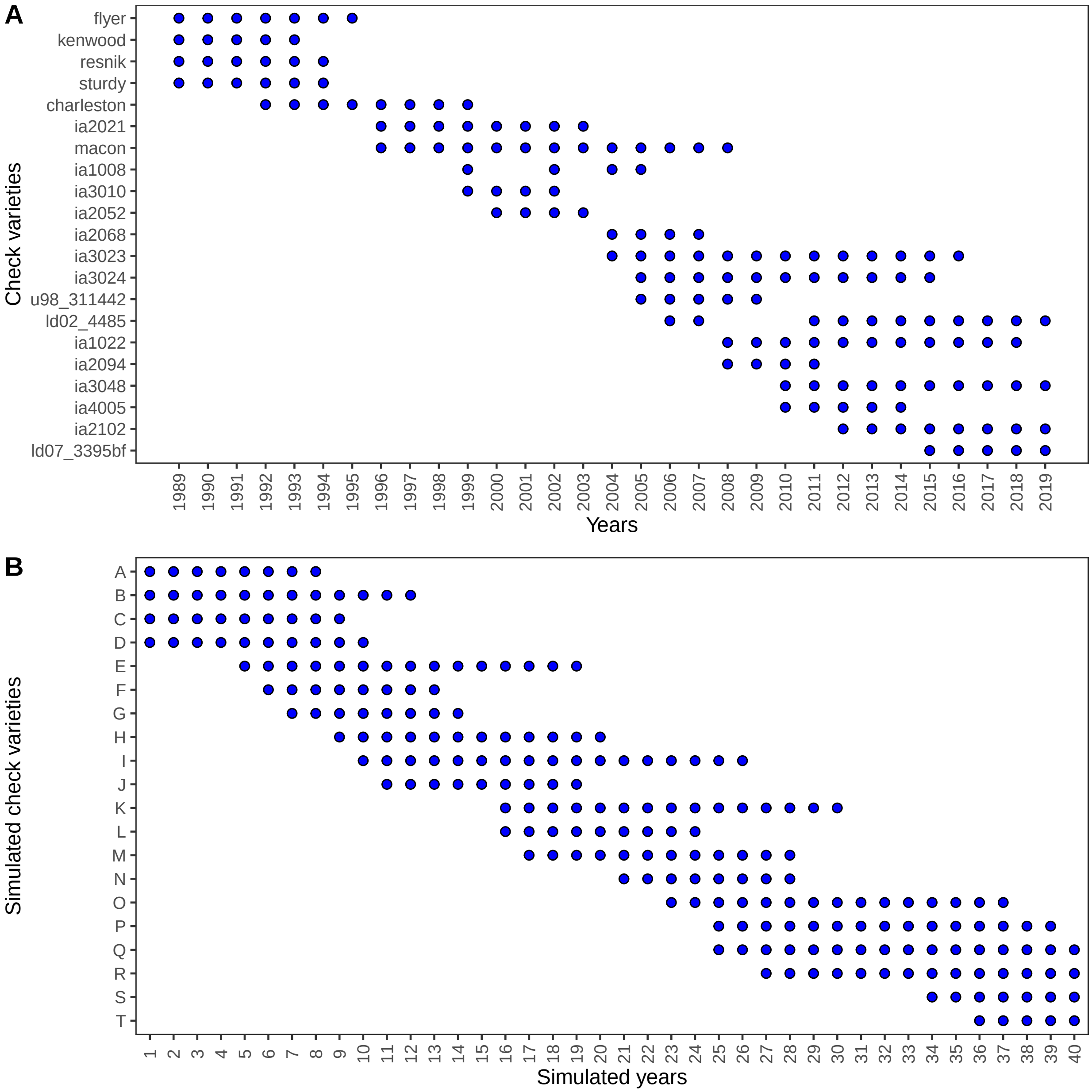

Figure A2: Frequency of empirical (A) and simulated (B) check cultivars.

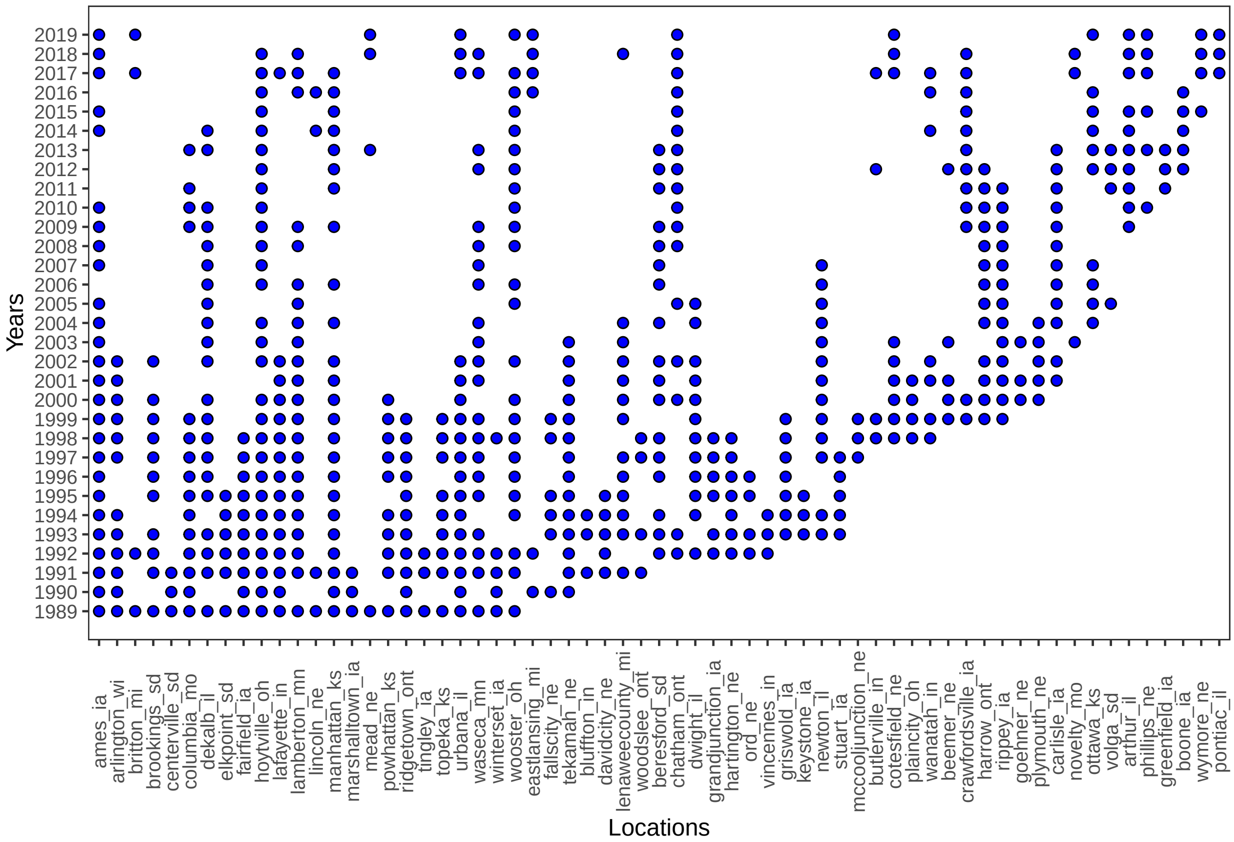

Figure A3: Frequency of locations from empirical data.

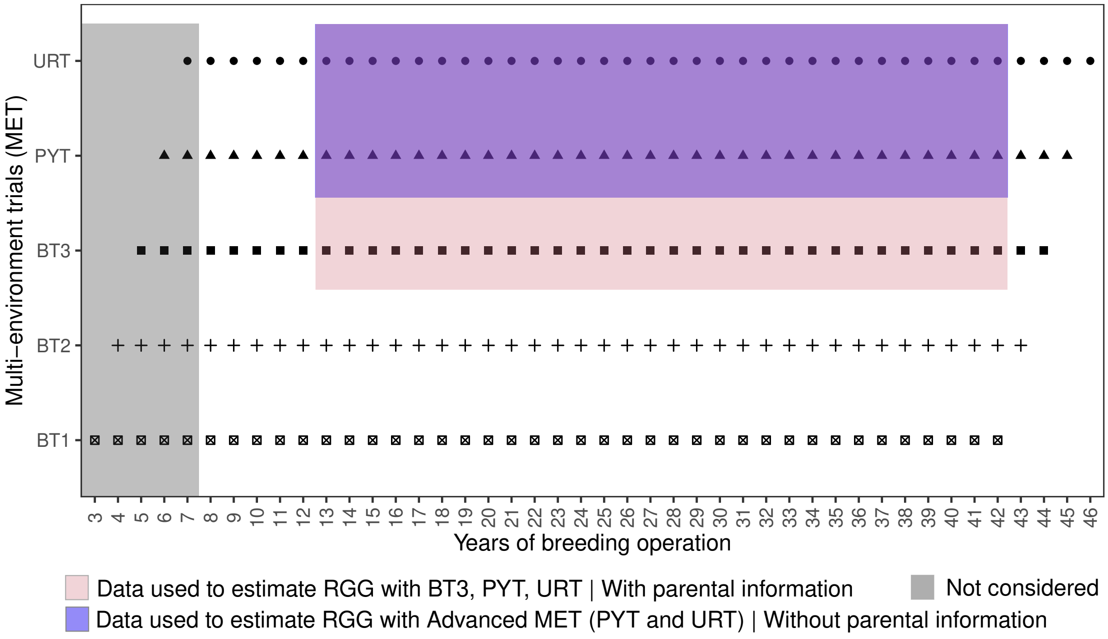

Figure A4: The structure of the simulated MET. Colors represent the source of data used to estimate RGG (with or without information from breeding lines).

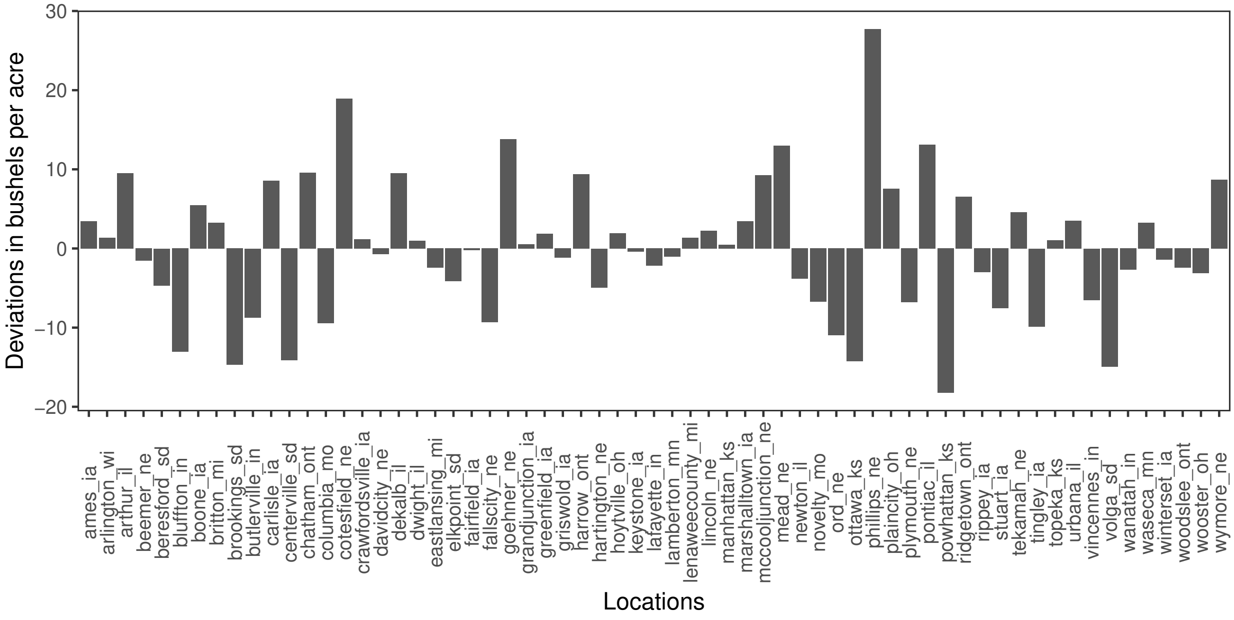

Figure A5: Estimated marginal values of location from empirical data used in the simulator.

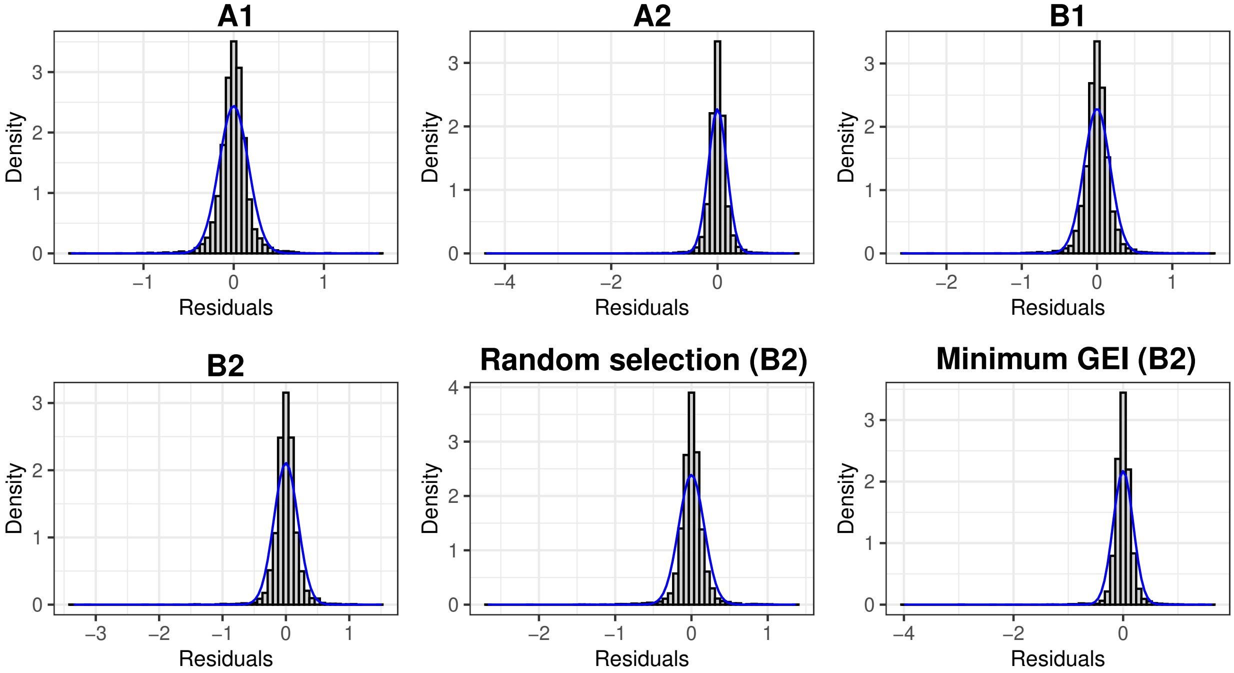

Figure A6: Histogram of estimated residuals from the analysis of variance performed to evaluate the average slope (RGG) of tested models (Tables 6 and 7).

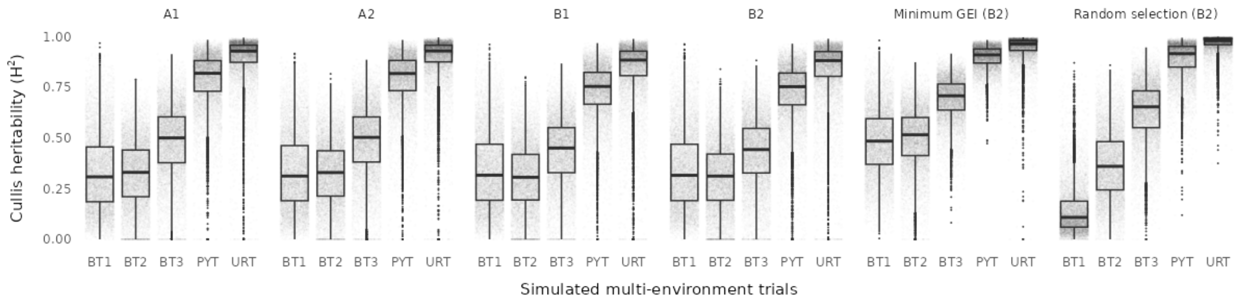

Figure A7: Estimates of broad sense heritabilities on an entry mean basis (Cullis et al. 2006) for each simulation model and trial.

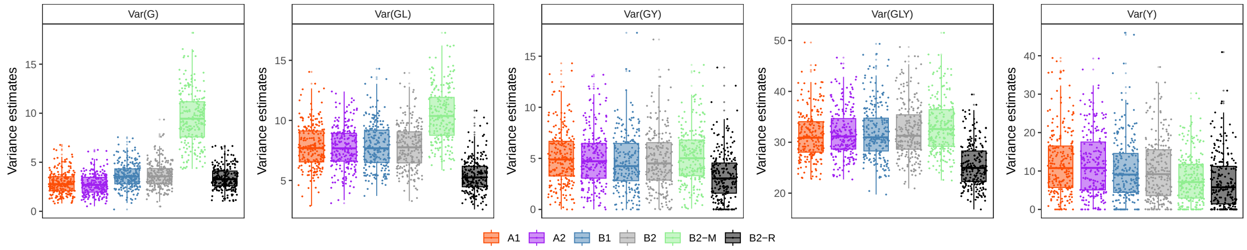

Figure A8: REML estimates of genotypic (G), year (Y), G × Location (L), G × Y, and G × L × Y variances for each simulation model (Table 2). Data were analyzed with Model 1 and included BT3, PYT, and URT.

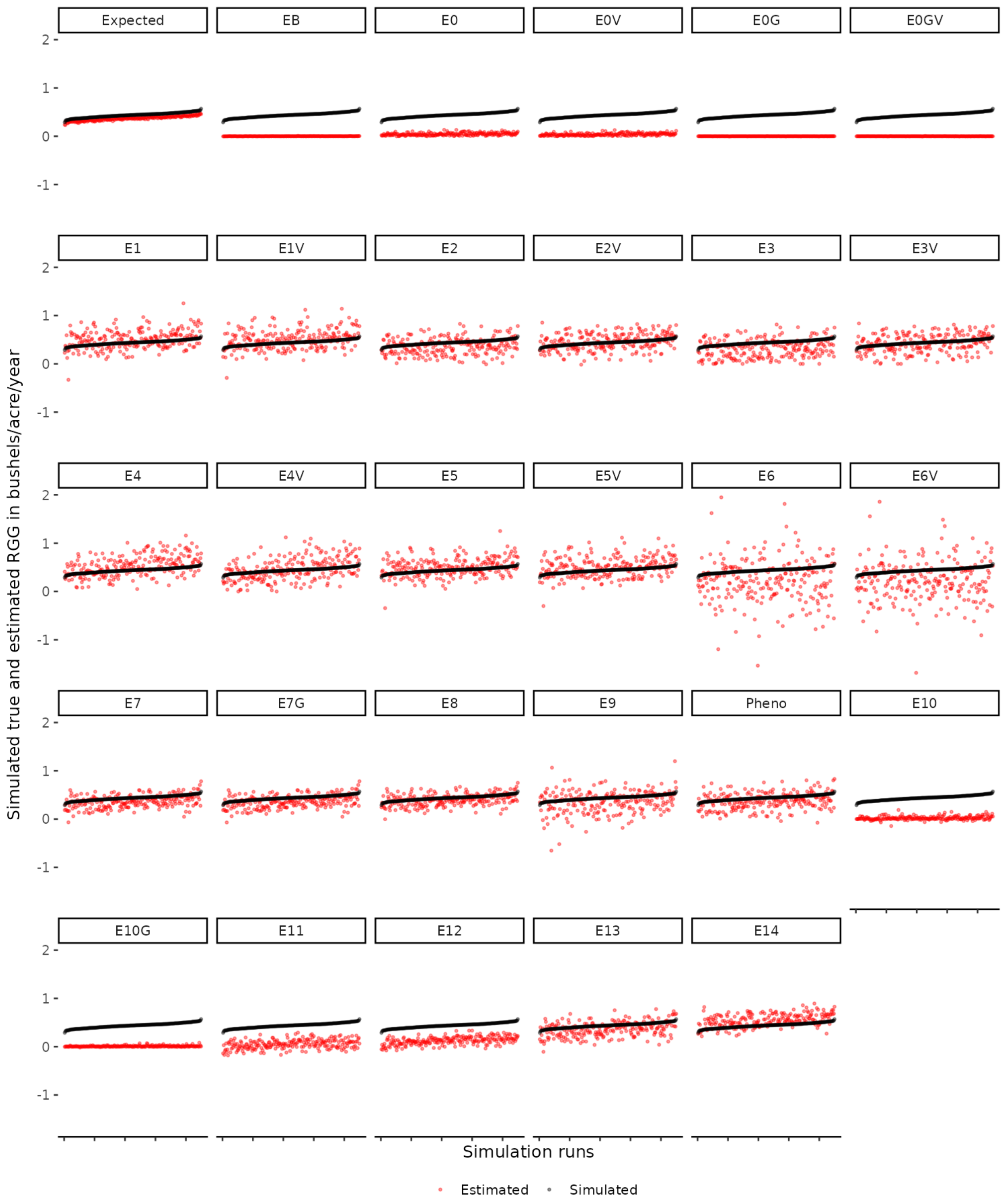

Figure A9: True (black) and estimated (red) RGG for evaluated models in scenario A1 (Tables 1 and 2). Check Section "Bias and linearity" for details on the "Expected" facet.

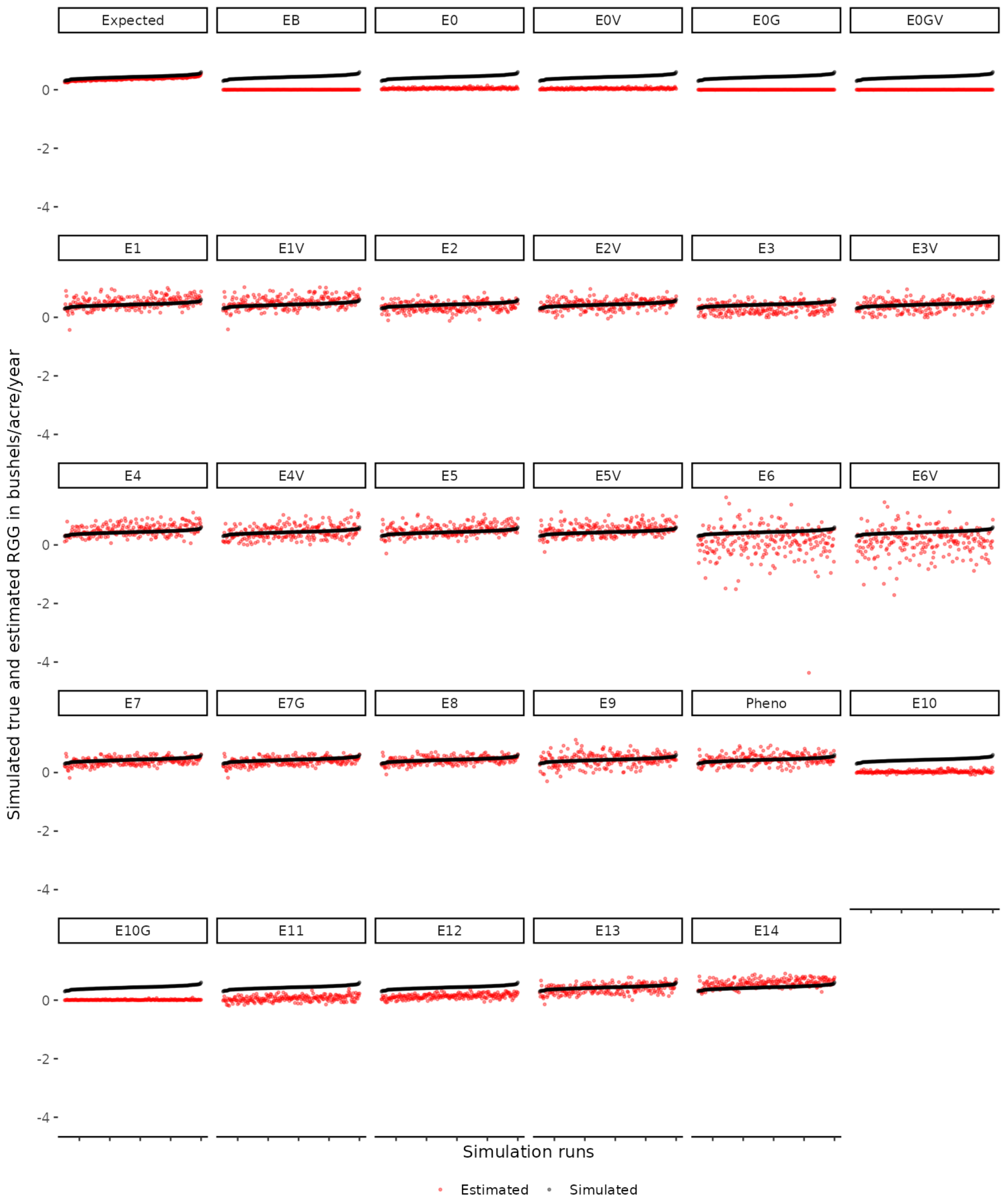

Figure A10: True (black) and estimated (red) RGG for evaluated models in scenario A2 (Tables 1 and 2). Check Section "Bias and linearity" for details on the "Expected" facet.

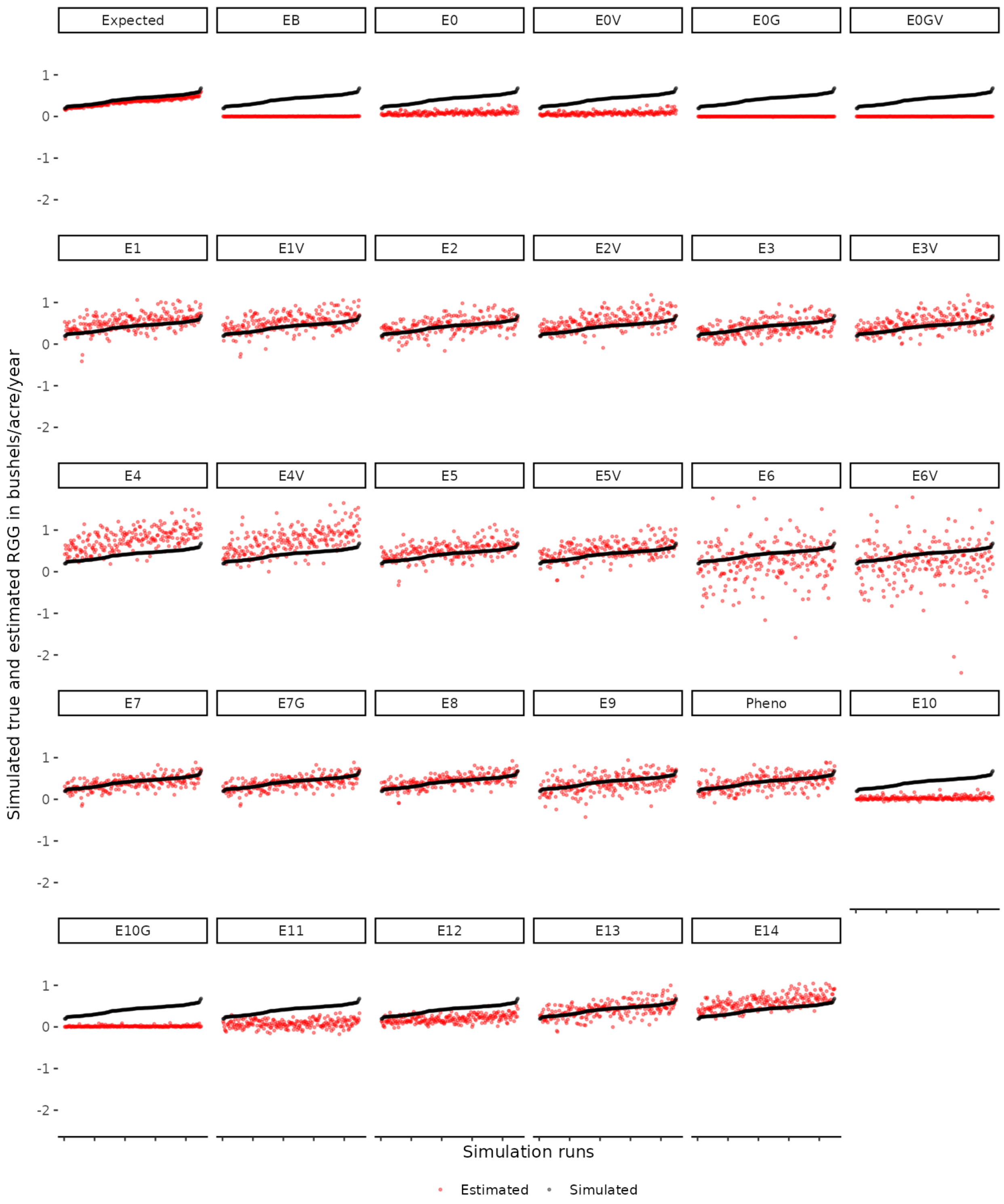

Figure A11: True (black) and estimated (red) RGG for evaluated models in scenario B1 (Tables 1 and 2). Check Section "Bias and linearity" for details on the "Expected" facet.

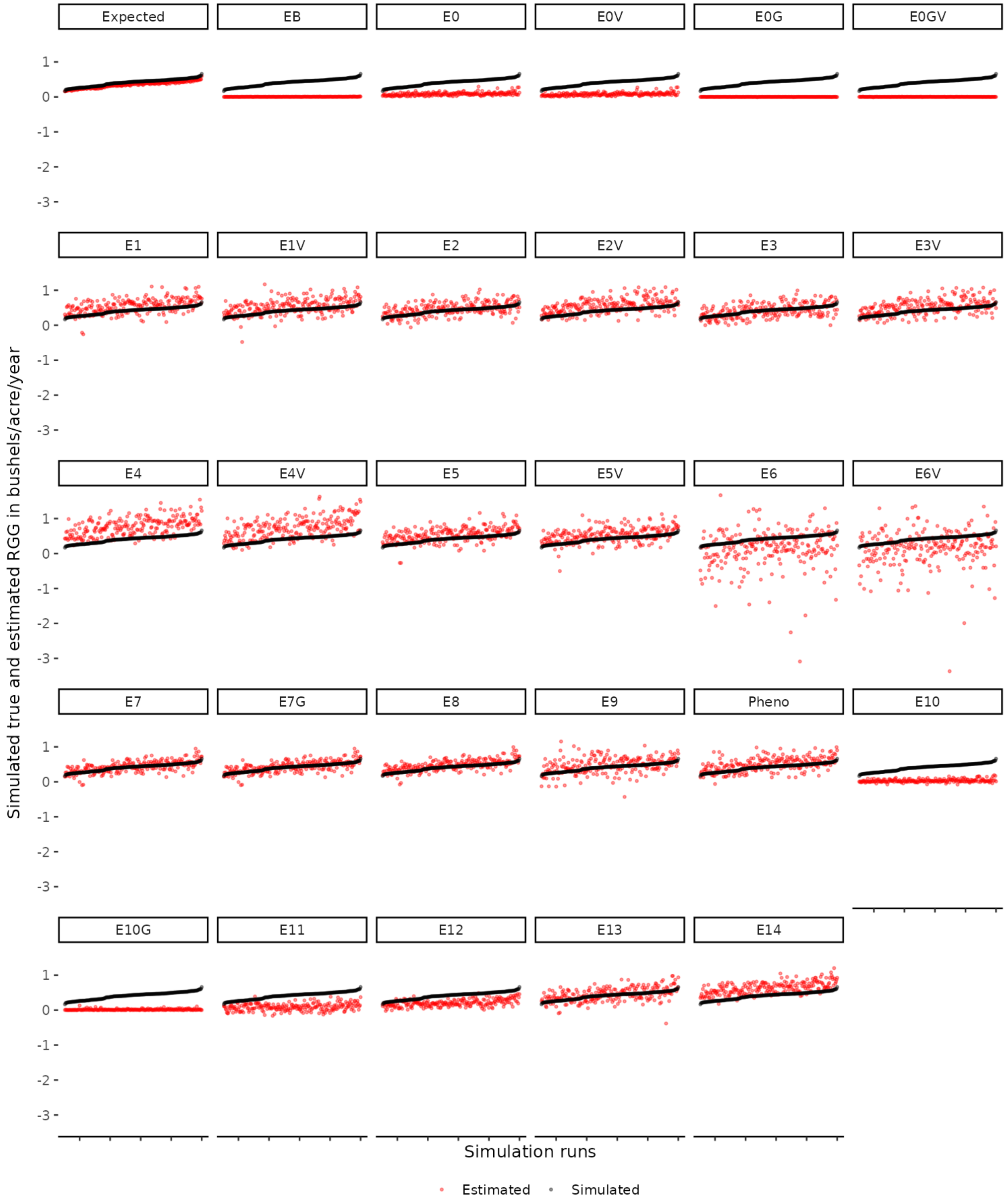

Figure A12: True (black) and estimated (red) RGG for evaluated models in scenario B2 (Tables 1 and 2). Check Section "Bias and linearity" for details on the "Expected" facet.

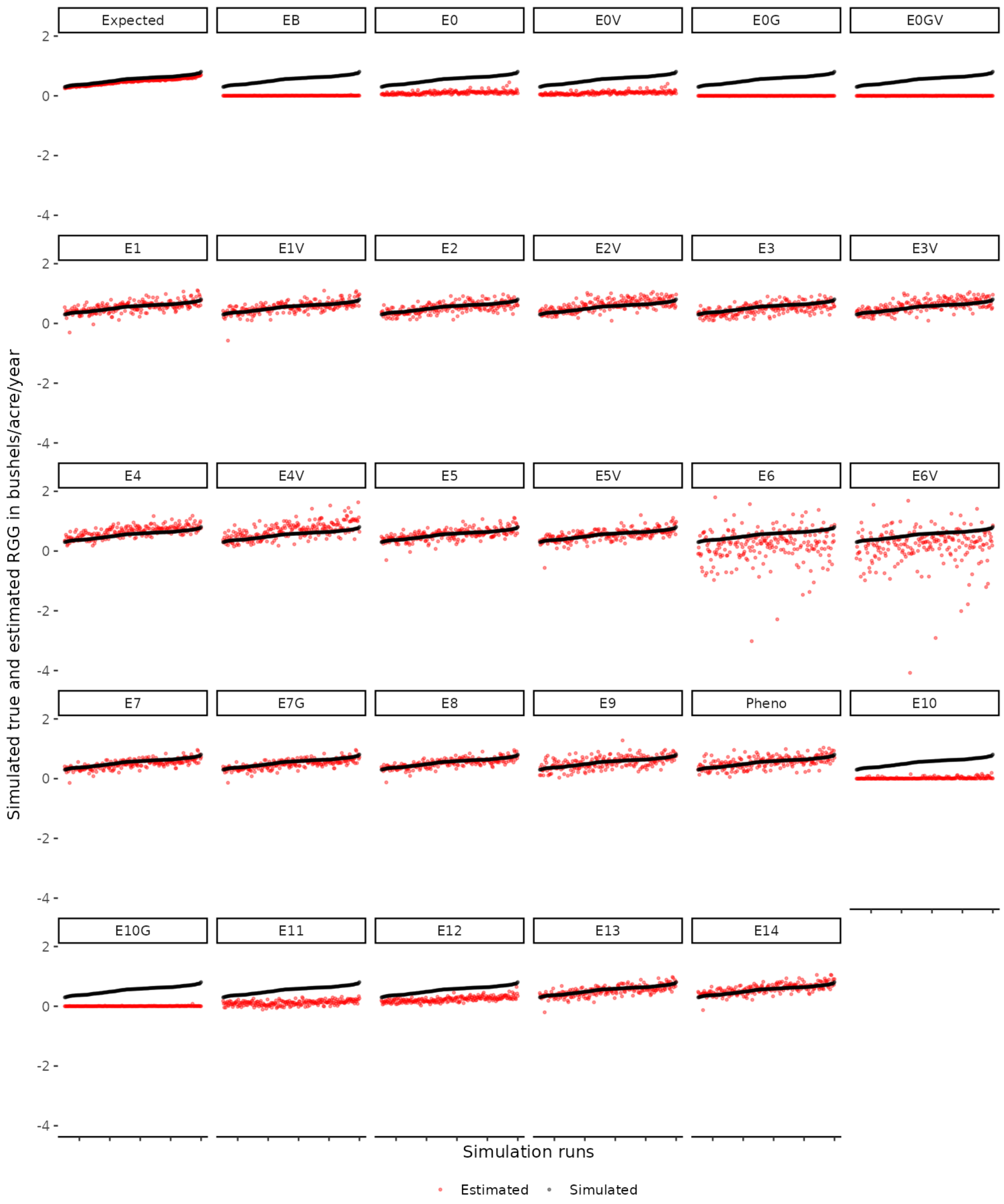

Figure A13: True (black) and estimated (red) RGG for evaluated models in scenario B2-M (Tables 1 and 2). Check Section "Bias and linearity" for details on the "Expected" facet.

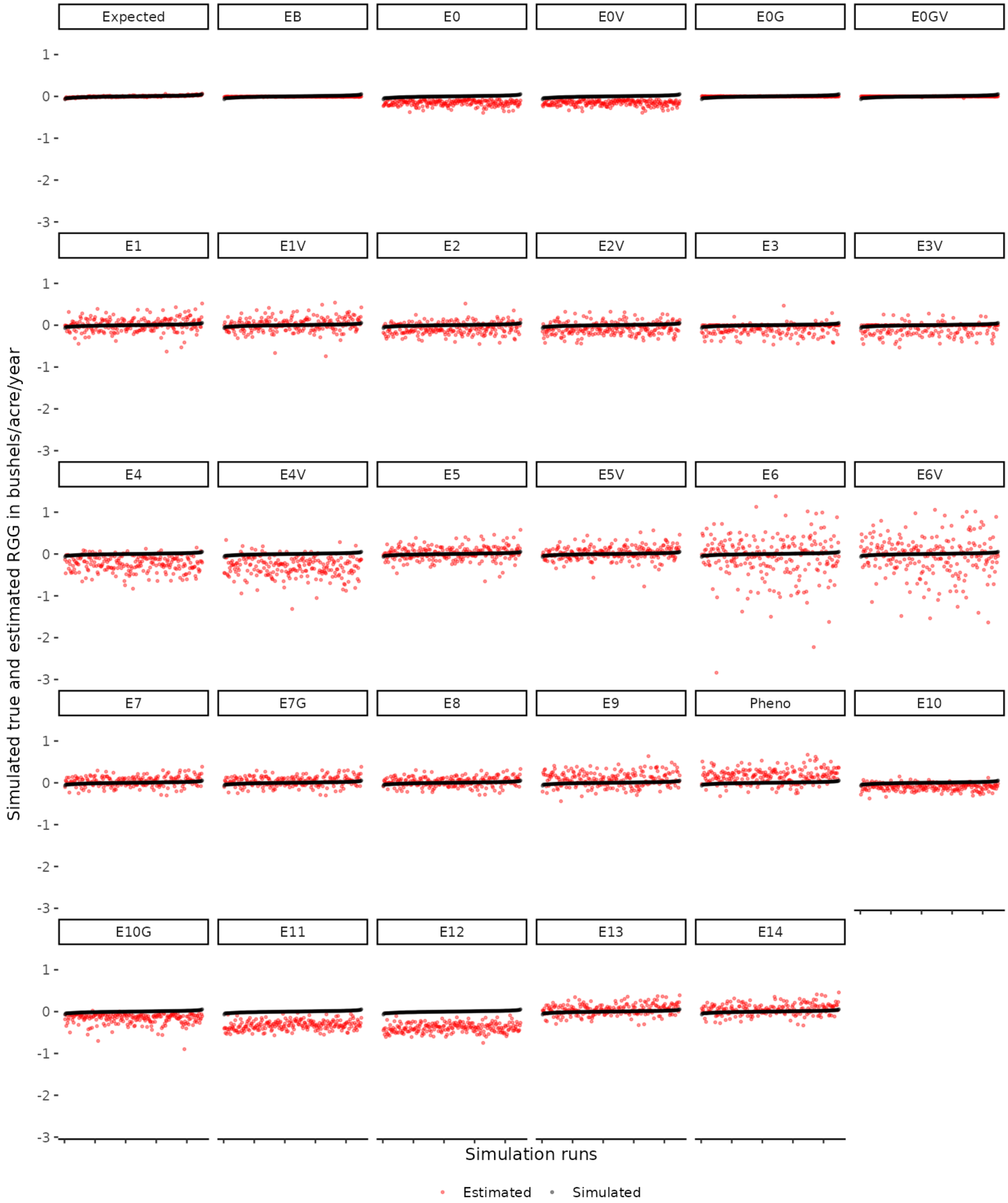

Figure A14: True (black) and estimated (red) RGG for evaluated models in scenario B2-R (Tables 1 and 2). Check Section "Bias and linearity" for details on the "Expected" facet.

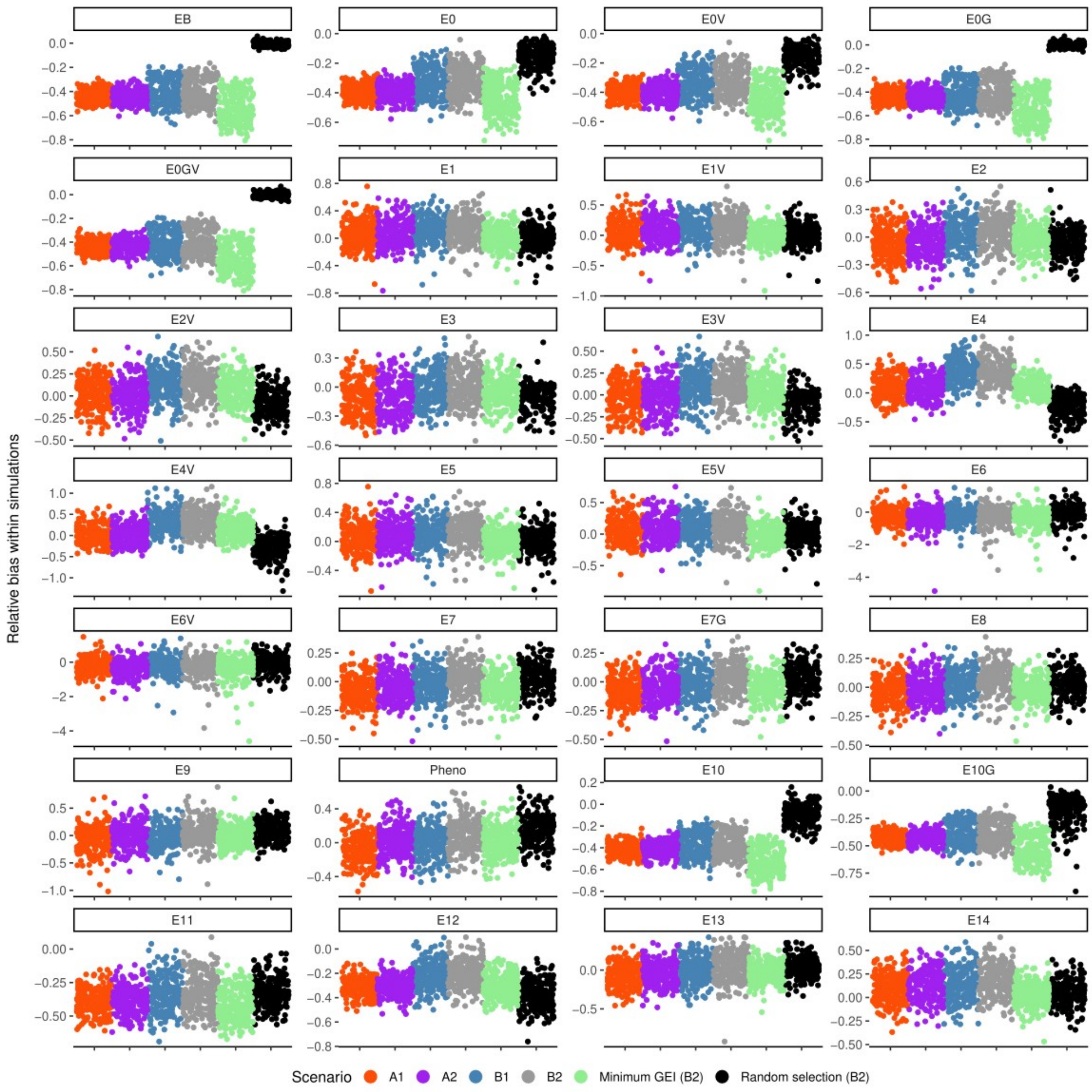

Figure A15: Estimated bias ($\hat{\xi}_s$) for each simulation run ($s = 1, \ldots, 225$).

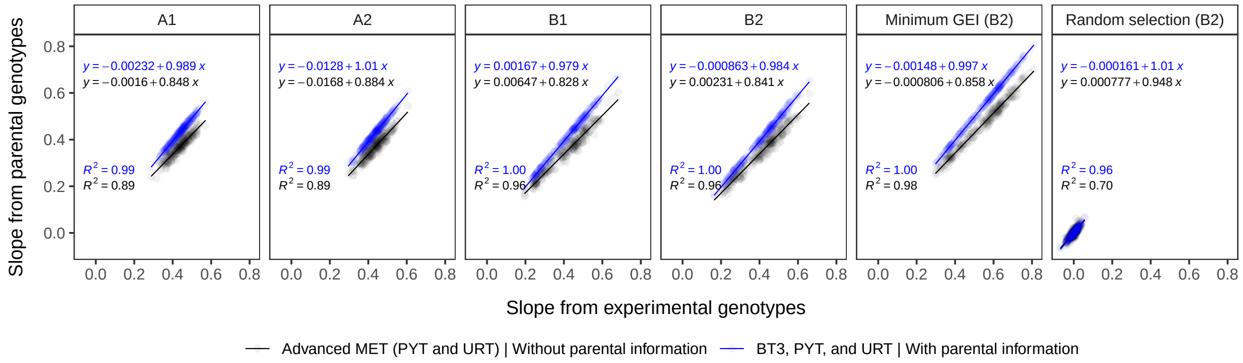

Figure A16: Linear regression (y) between true simulated genetic values of parental and experimental genotypes. The R^2 represents the coefficient of determination from the fitted (blue/black) regression lines. The slope on the y-axis is $\beta_{T_{(r)}}$ from Model 3, and on the x-axis is $\beta_{R_{(r)}}$ from Model 4.

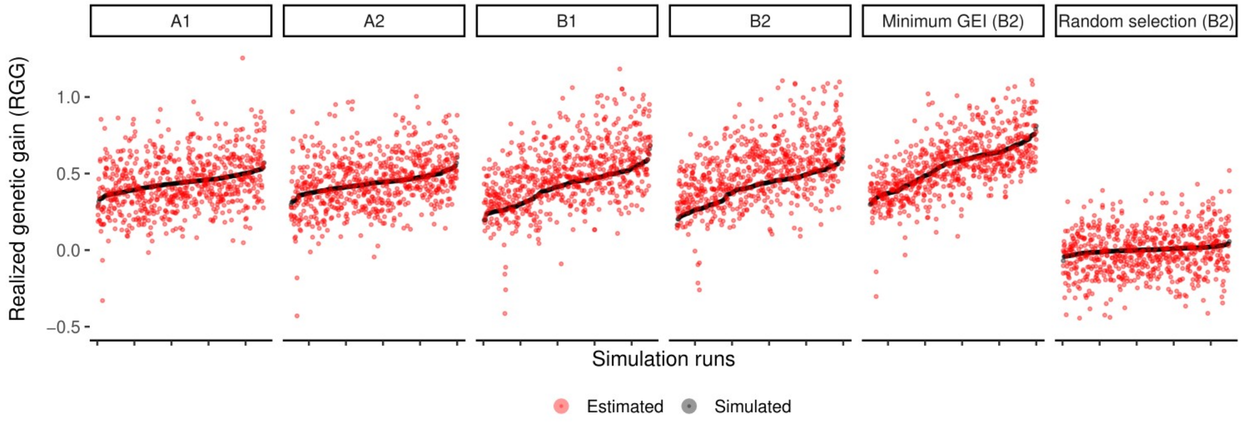

Figure A17: Scatter plot of simulated (black) and estimated (red) RGG values from Models E1, E2V, and E7 (Table 6).

Table A1: Number of positive (P) and negative (N) estimates of RGG.

Scenarios	A1		A2		B1		B2		B2-M		B2-R	
Model	P	N	P	N	P	N	P	N	P	N	P	N
Simulated	225	0	225	0	225	0	225	0	225	0	123	102
EB	135	90	140	85	181	44	184	41	219	6	7	218
E0	223	2	224	1	225	0	225	0	225	0	0	225
E0V	224	1	224	1	225	0	225	0	225	0	0	225
E0G	64	161	69	156	62	163	80	145	67	158	93	132
E0GV	80	145	78	147	97	128	96	129	57	168	94	131
E1	224	1	224	1	223	2	223	2	223	2	119	106
E1V	224	1	224	1	222	3	222	3	224	1	123	102
E2	224	1	221	4	220	5	224	1	225	0	71	154
E2V	224	1	223	2	224	1	225	0	225	0	66	159
E3	224	1	224	1	223	2	225	0	225	0	54	171
E3V	224	1	225	0	225	0	225	0	225	0	56	169
E4	225	0	225	0	225	0	225	0	225	0	16	209
E4V	225	0	225	0	225	0	225	0	225	0	19	206
E5	224	1	224	1	223	2	223	2	223	2	123	102
E5V	224	1	224	1	222	3	222	3	224	1	124	101
E6	163	62	146	79	171	54	153	72	163	62	109	116
E6V	169	56	149	76	176	49	159	66	170	55	110	115
E7	224	1	224	1	223	2	222	3	224	1	141	84
E7G	224	1	224	1	223	2	222	3	224	1	140	85
E8	224	1	224	1	223	2	223	2	224	1	137	88
E9	211	14	218	7	219	6	218	7	225	0	154	71
Pheno	223	2	225	0	224	1	224	1	225	0	189	36
E10	174	51	182	43	188	37	184	41	216	9	29	196
E10G	203	22	208	17	221	4	221	4	220	5	2	223
E11	158	67	157	68	182	43	184	41	202	23	0	225
E12	207	18	212	13	221	4	223	2	225	0	0	225
E13	224	1	224	1	222	3	222	3	224	1	150	75
E14	225	0	225	0	225	0	225	0	224	1	151	74

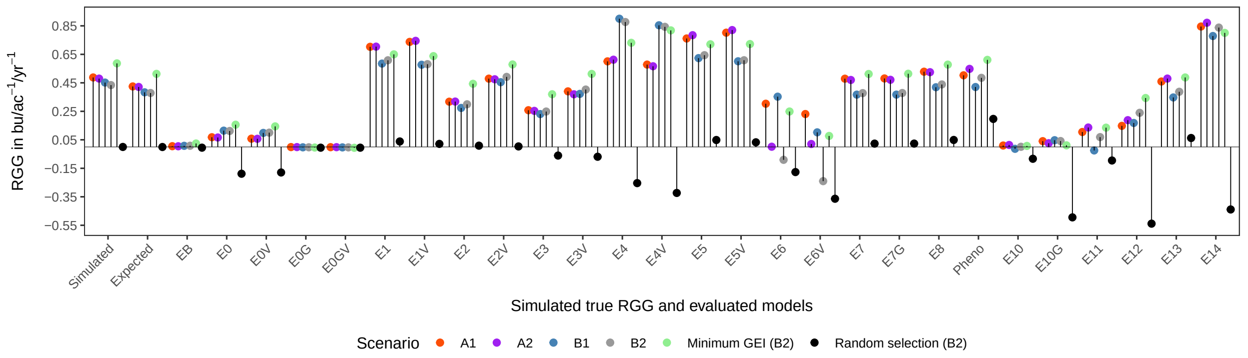

Figure A18: Average values for true RGG ($\hat{\beta}_T$), expected RGG ($\hat{\beta}_R^{true}$), and estimated RGG ($\hat{\beta}_R, \hat{\beta}_g, \hat{\beta}_t, \hat{\beta}_f$) for the evaluated models (x-axis), considering 10 years of MET. Model E9 (Table 6) was not included.

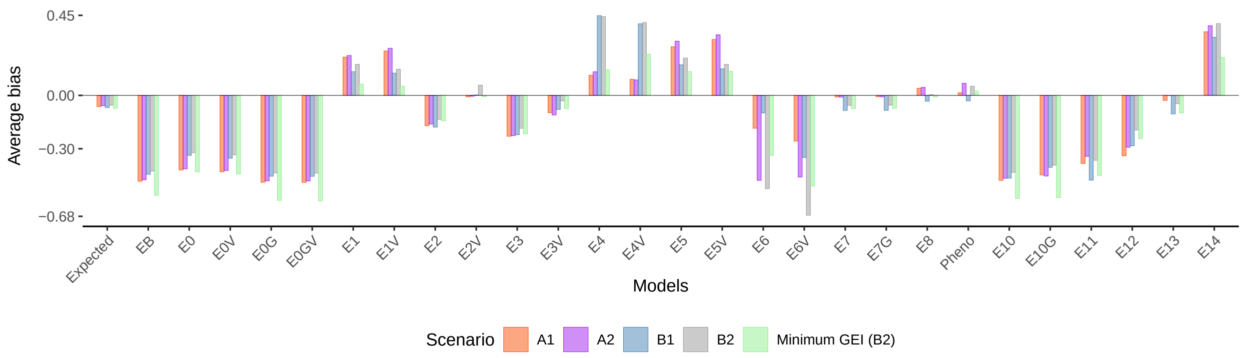

Figure A19: Average bias in bu/ac^{-1}/yr^{-1} considering 10 years of MET. Model E9 (Table 6) was not included.

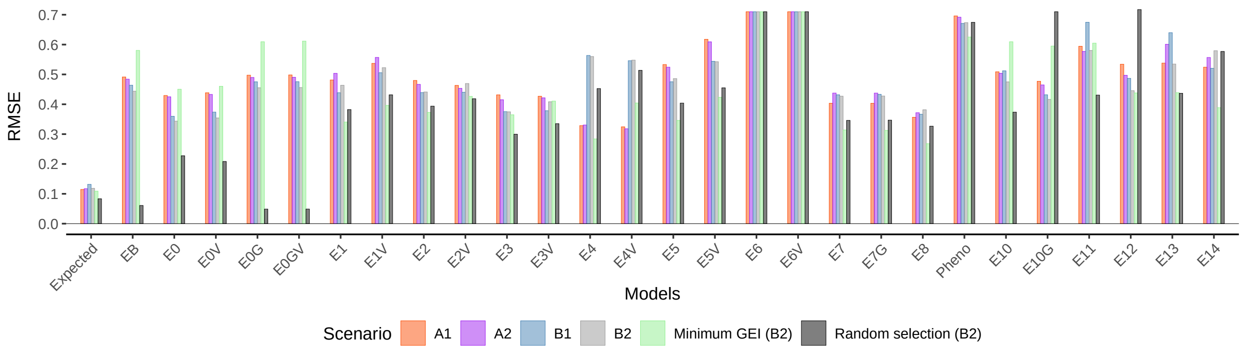

Figure A20: Root mean squared error (RMSE) considering 10 years of MET. Values were bounded at 0.72 units to enhance visualization. Model E9 (Table 6) was not included.

Table A2: Goodness-of-fit for models considered to estimate the RGG from the soybean empirical data. Fixed effects are underlined, and the RGG was estimated with model terms highlighted in gray. The numbers in parentheses (1, 2) in the model's description represent steps to fit the models. Models in step one (1) were adjusted only with check cultivars. Models E2, $E2_1, \ldots, E2_6$, were evaluated according to the Akaike information criterion (Akaike 1974, AIC), and the RGG reported with the selected model. For further details on model terms and covariance modeling, check Table 6.

Models					AIC
Description	ID	VCOV			
		G	Σ_{gl}	Σ_{gy}	
$\hat{y}_{ijk} = \underline{G_i} + L_j + \underline{Y_k} + \underline{\delta z} + GL_{ij} + GY_{ik} + LY_{jk} + GLY_{ijk}$	E1	**I**	**I** $\otimes$ **I**	**I** $\otimes$ **I**	159411.7
	E2	-	**I** $\otimes$ **I**	**I** $\otimes$ **I**	175764.9
	$E2V_1$	-	**D** $\otimes$ **I**	**D** $\otimes$ **I**	174366.1
$\hat{y}_{ijk} = \underline{\beta_g r_i} + L_j + \underline{\beta_t t_k} + \underline{\delta z} + GL_{ij} + GY_{ik} + LY_{jk} + GLY_{ijk}$	$E2V_2$	-	$\mathbf{FA_1} \otimes \mathbf{I}$	$\mathbf{FA_1} \otimes \mathbf{I}$	172889.9
	$E2V_3$	-	$\mathbf{FA_2} \otimes \mathbf{I}$	$\mathbf{FA_2} \otimes \mathbf{I}$	172582.2
	$E2V_4$	-	$\mathbf{FA_3} \otimes \mathbf{I}$	$\mathbf{FA_2} \otimes \mathbf{I}$	172380.3
	$E2V_5$		$\mathbf{FA_3} \otimes \mathbf{I}$	$\mathbf{FA_3} \otimes \mathbf{I}$	172399.0
	$E2V_6$		$\mathbf{FA_4} \otimes \mathbf{I}$	$\mathbf{FA_2} \otimes \mathbf{I}$	172326.3
$\hat{y}_{ih} = \underline{G_i} + E_h + EG_{ih}$ (1)<br>$\hat{y}_{ih} = G_i + \underline{\lambda_E \hat{E}_h} + G\hat{E}_h$ (2)	E7	**I**	-		211842.9

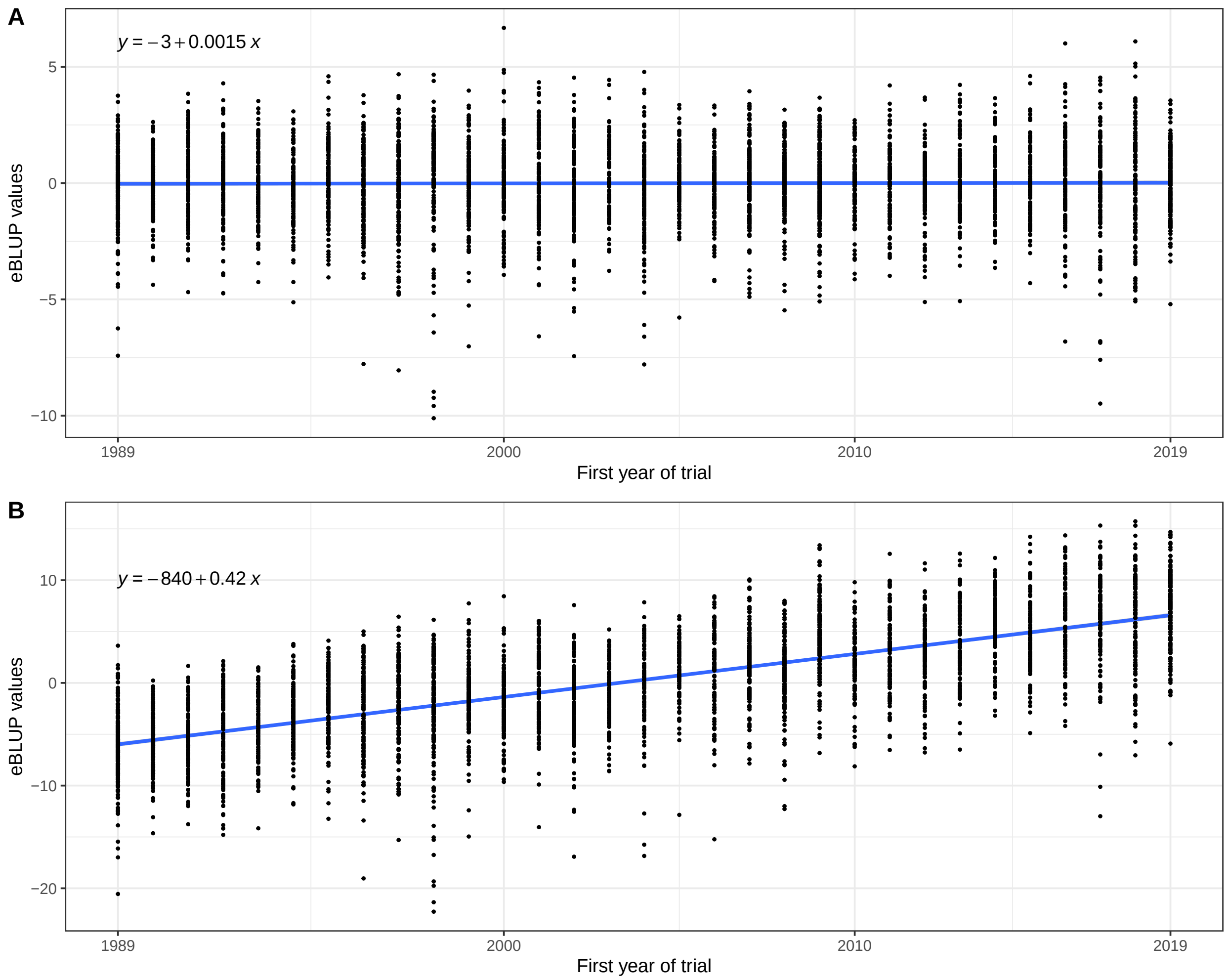

Figure A21: Predicted eBLUP values from the benchmark Model E0 (A) and from Model E7 (B) for the soybean empirical data. The blue line is from the fitted regression of the predicted values as a function of the first year of testing. For details on the models check Table 6.

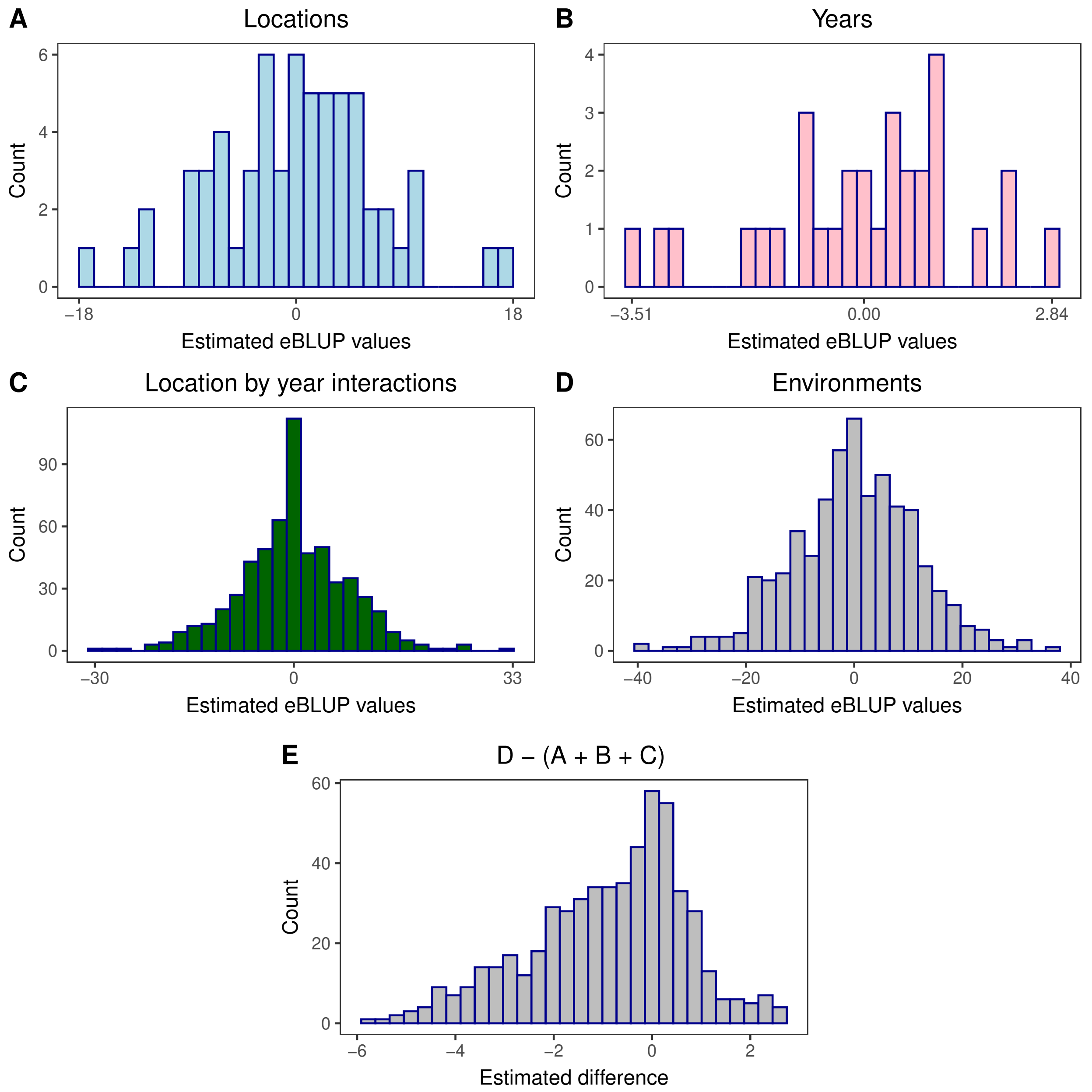

Figure A22: Estimated eBLUP values of locations (A), years (B), location by year interaction effects (C), and of environmental (location-year combination) effects (D) from check cultivars for the empirical data. These predicted values in D were used in Model E7 to calculate RGG for the empirical data.

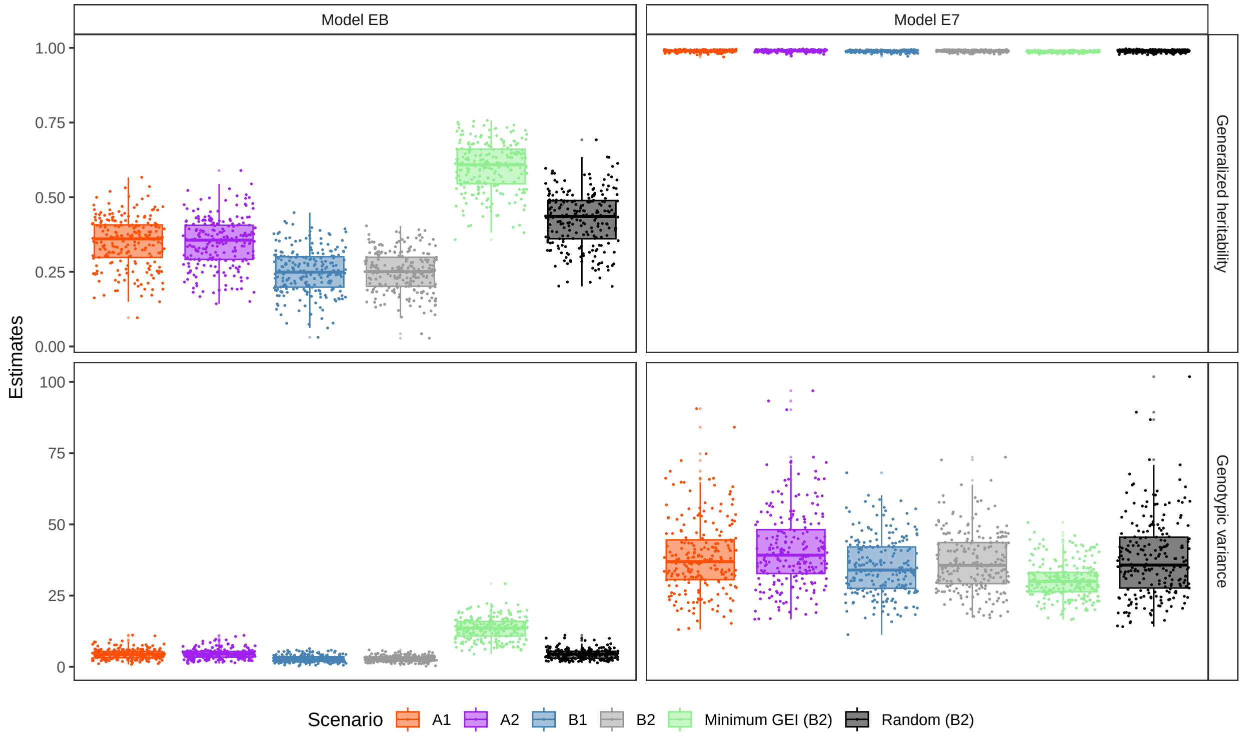

Figure A23: REML estimates of genotypic variance and generalized heritability (Cullis et al. 2006) for benchmark Model EB and E7 (Table 6).

B - Trait simulation in AlphaSimR

Additive (or total) QTL effects for the simulated traits in AlphaSimR (Gaynor et al. 2021) are simulated in two steps. The first one consists of sampling the effects either from a Standard Normal or Gamma distribution (the user must define its shape parameter). The second step applies a scaling factor $\left(\sqrt{\frac{\text{user-defined variance}}{\text{initially sampled variance}}}\right)$ to match the user-defined additive (or total) variance. Then, an intercept (user-defined) is added to the simulated (and now scaled) QTL effects. This step mimics the introduction of a trait mean reference to which trait the user is simulating. In the case of a purely additive trait, the variance of the simulated breeding values in the founder population will match the user-defined additive variance. For further details, check the vignette "Traits in AlphaSimR" (`https://cran.r-project.org/web/packages/AlphaSimR/vignettes/traits.pdf`, accessed on 09/07/2022).

C - Phenotypic models used in the simulator for parental selection, yearly selections, and for single-trial analyses

Parental genotypes were selected in BT3 after three years of trials. Data from BT1, BT2, and BT3 was combined, and the following linear mixed model was considered:

$$\hat{y}_{ijk} = G_i + L_j + \underline{Y_k} + GL_{ij} + GY_{ik} + LY_{jk} + GLY_{ijk} + \epsilon_{ijk} \tag{C1}$$

where $\hat{y}_{ijk}$ is the vector of eBLUE values of genotypic means for the ijk^{th} genotype × location × year combination estimated from single-trial analyses, and G_i, L_j , and Y_k, are the main effects of the ith genotype, j^{th} location, and k^{th} years, respectively, followed by all two and three-way interaction effects. All model terms but Y_k (underlined) were considered random effects. The error variance $\sigma^2_{\epsilon_{ijk}}$ of ϵ_{ijk} is assumed to be known from the analyses of the individuals' trials (Model C3), where the residual variance matrix is a diagonal matrix with elements equal to $\frac{1}{\text{SE}^2}$, where SE is the estimated standard error (Smith et al. 2001; Frensham et al. 1997). Yearly selections and the analyses of single trials were performed with the following models, respectively:

$$\hat{y}_{ij..} = G_i + \underline{L_j} + GL_{ij} + \epsilon_{ij..} \tag{C2}$$

$$y_{ilb} = \underline{G_i} + \epsilon_{ilb} \tag{C3}$$

where y_{ilb} is the observed yield in a plot level at any given trial. The eBLUE values of genotypic ($\hat{y}_{ij..}$, $\hat{y}_{ijk}$) means were estimated with weighted least squares, so that Var(ϵ_{ilb}) = $\frac{\epsilon^2_{ilb}}{n_i}$, where n_i is the number of plots of the ith genotype in the bth trial. Thus, genotypes have heterogeneous SE within trials due to the insertion of missing data, i.e., a random proportion from zero to 12% of the phenotypic data was considered missing data within trials. A basic explanation of stage-wise modeling is given in Appendix F.

D - Simulation of correlated additive QTL effects for GL and GY, and GLY

In terms of variance components, the genotype by environment interactions (GEI) is composed of genotype × location (GL, σ^2_{GL}), genotype × year (GY, σ^2_{GY}), and genotype × location × year (σ^2_{GLY}) interaction variances. Both σ^2_{GY} and σ^2_{GLY} are non-static (unrepeatable) sources of variation, while the static portion of the σ^2_{GL} is repeatable across years (Yan 2016). Krause et al. (2023) investigated the GEI patterns in a historical soybean dataset composed of 39,006 phenotypic records from 1989-2019. This data comprises trials conducted by public plant breeders in the Northern Region of the U.S., and it embraces 4,257 experimental genotypes, 63 locations, and 31 years. The modeling scenarios B1 and B2 (Section "Variations of simulated MET models") were designed to capture the structure of this historical data as best as possible.

The following algorithm is a heuristic approach built to simulate correlated additive QTL effects in multi-environment trials (MET) according to GL and GY effects. For example, if 1,000 QTLs were simulated and the goal is phenotyping in $L = 10$ locations across $Y = 2$ years, the algorithm will initially sample 10 unique variance components for GL and two unique variance components for GY. The variances for individual locations and years are sampled from a mixture of three and six Log-Logistic distributions, respectively (Tables 1 and E1). For more details on how these empirical probability distributions of variance components were obtained, please refer to Appendix E. The initial diagonal variance-covariance (VCOV) matrices for GL (Σ_{GL}) and GY (Σ_{GY}) are as follows:

$$\boldsymbol{\Sigma_{GL}} = \begin{bmatrix} \sigma^2_{GL_1} & 0 & \dots & 0 \\ 0 & \sigma^2_{GL_2} & \dots & 0 \\ \vdots & \vdots & \ddots & \vdots \\ 0 & 0 & \dots & \sigma^2_{GL_{10}} \end{bmatrix} \qquad \boldsymbol{\Sigma_{GY}} = \begin{bmatrix} \sigma^2_{GY_1} & 0 \\ 0 & \sigma^2_{GY_2} \end{bmatrix}$$

These variance components (i.e., diagonal matrices) are then set relative to the predefined additive genetic variance (σ^2_G) of the simulated trait. For example, if the predefined $\sigma^2_G = 5$ and $\sigma^2_{GL_1} = 10$, then the variance of GL effects represents two times the magnitude of the additive genetic variance, or $\sigma^2_{GL_1}/\sigma^2_G = 10/5 = 2$. As a reminder, scenarios A define a unique σ^2_G from a point estimate of variance, and scenario B sample from empirical distributions a unique σ^2_G in each simulation run r ($\sigma^2_{G_r}$). Next, the relative variances are multiplied by the variance of the simulated additive effects (γ), as follows:

$$\Sigma_{GL} = \begin{bmatrix} \left(\frac{\sigma^2_{GL_1}}{\sigma^2_G}\right)\times\gamma & 0 & \dots & 0 \\ 0 & \left(\frac{\sigma^2_{GL_2}}{\sigma^2_G}\right)\times\gamma & \dots & 0 \\ \vdots & \vdots & \ddots & \vdots \\ 0 & 0 & \dots & \left(\frac{\sigma^2_{GL_{10}}}{\sigma^2_G}\right)\times\gamma \end{bmatrix} \quad \Sigma_{GY} = \begin{bmatrix} \left(\frac{\sigma^2_{GY_1}}{\sigma^2_G}\right)\times\gamma & 0 \\ 0 & \left(\frac{\sigma^2_{GY_2}}{\sigma^2_G}\right)\times\gamma \end{bmatrix}$$

Which are equivalent to:

$$\Sigma^{\text{relG}}_{GL} = \begin{bmatrix} \sigma^2_{GL_1^{\text{relG}}} & 0 & \dots & 0 \\ 0 & \sigma^2_{GL_2^{\text{relG}}} & \dots & 0 \\ \vdots & \vdots & \ddots & \vdots \\ 0 & 0 & \dots & \sigma^2_{GL_{10}^{\text{relG}}} \end{bmatrix} \quad \Sigma^{\text{relG}}_{GY} = \begin{bmatrix} \sigma^2_{GY_1^{\text{relG}}} & 0 \\ 0 & \sigma^2_{GY_2^{\text{relG}}} \end{bmatrix}$$

Where the "relG"stands for relative to the genetic variance. The VCOV matrices $\Sigma^{\text{relG}}_{GL}$ and $\Sigma^{\text{relG}}_{GY}$ are now modified to include covariances between locations and years, receptively. Genotypic correlation between 63 locations and 31 years was obtained by Krause et al. (2023). Briefly, the authors fitted 20 linear mixed models to the soybean historical dataset (see Table 1 in their publication) and selected the best-fit one according to some evaluation criteria. Correlations were then calculated from the best-fit model which included factor-analytic (FA) covariance structures for both GL (ρ^{FA}_{GL}, Figure D2) and GY (ρ^{FA}_{GY}, Figure D1) model terms. With the pre-sampled variances (i.e., the diagonal values from $\Sigma^{\text{relG}}_{GL}$ and $\Sigma^{\text{relG}}_{GY}$) and given the correlations ($\rho_{l,l'}$, $\rho_{y,y'}$) are known, the covariances among any pair of years and/or locations were computed as $COV(X_i, X_{i'}) = \sigma_{i,i'} = \rho_{i,i'} \times \sqrt{\sigma^2_i \times \sigma^2_{i'}}$. Thus,

$$\Sigma^{\text{relG}}_{GL} = \begin{bmatrix} \sigma^2_{GL_1^{\text{relG}}} & \sigma_{1,2} & \dots & \sigma_{1,10} \\ \sigma_{2,1} & \sigma^2_{GL_2^{\text{relG}}} & \dots & \sigma_{2,10} \\ \vdots & \vdots & \ddots & \vdots \\ \sigma_{10,1} & \sigma_{10,2} & \dots & \sigma^2_{GL_{10}^{\text{relG}}} \end{bmatrix} \quad \Sigma^{\text{relG}}_{GY} = \begin{bmatrix} \sigma^2_{GY_1^{\text{relG}}} & \sigma_{1,2} \\ \sigma_{2,1} & \sigma^2_{GY_2^{\text{relG}}} \end{bmatrix}$$

Correlated additive QTL effects can now be drawn from a Multivariate Normal Distribution according to the computed covariance matrices above. So, the distributions of the QTL effects for GL and GY are as follows:

$$QTL_{GL} \sim N(\mathbf{0}, \Sigma^{\text{relG}}_{GL}) \quad \text{and} \quad QTL_{GY} \sim N(\mathbf{0}, \Sigma^{\text{relG}}_{GY})$$

And the final matrices of QTL effects are of the following form:

$$
\mathbf{QTL_{GL}} = \begin{array}{c} \\ \left[\begin{array}{ccccc} \text{QTL 1} & \text{QTL 2} & \text{QTL 3} & \dots & \text{QTL 1000} \\ 0.0646 & -0.0422 & 0.0575 & \dots & 0.0872 \\ -0.0250 & 0.0554 & 0.2469 & \dots & 0.0583 \\ \vdots & \vdots & \vdots & \vdots & \vdots \\ 0.0227 & 0.1042 & -0.0647 & \dots & 0.0131 \end{array}\right] \end{array} \begin{array}{l} \\ \text{Location 1 (Ames - IA)} \\ \text{Location 2 (Arlington - WI)} \\ \vdots \\ \text{Location 10 (Butlerville - IN)} \end{array}
$$

$$
\mathbf{QTL_{GY}} = \begin{array}{c} \begin{array}{ccccc} \text{QTL 1} & \text{QTL 2} & \text{QTL 3} & \dots & \text{QTL 1000} \end{array} \\ \left[\begin{array}{ccccc} 0.0474 & -0.1209 & -0.0136 & \dots & 0.1045 \\ 0.0139 & -0.0432 & -0.0450 & \dots & -0.0560 \end{array}\right] \end{array} \begin{array}{l} \text{Year 1} \\ \text{Year 2} \end{array}
$$

The original correlation matrices for GL and GY obtained by Krause et al. (2023) from FA models contained 63 locations and 31 years, respectively. For this work, a unique simulation run never explored more than 63 locations across the 46 years of the simulated breeding program. Hence, there was enough empirical information in $\boldsymbol{\rho}_{\boldsymbol{GL}}^{\boldsymbol{FA}}$ (Figure D2) to simulate correlated GL effects. On the other hand, for the GY effects, the correlation matrix is of dimension 31×31 (Figure D1). To overcome this issue, our algorithm symmetrically augments $\boldsymbol{\rho}_{\boldsymbol{GY}}^{\boldsymbol{FA}}$ according to the desired number of years. Briefly, the algorithm explores the empirical correlations to simulate the first 31 years of MET. New correlations are then simulated by adding noise, in a highly controlled manner (Hardin et al. 2013, function `noisecor`), to the empirical values. If the resulting covariance matrix is not positive-definite (PD), it computes the nearest PD matrix while keeping the pre-sampled variances (diagonal values) constant (Bates and Maechler 2021, function `nearPD`). Examples of the sampled QTL effects are shown below in Figures D3 and D4.

Correlated additive QTL effects for scenarios B1 and B2 were computed as described up to this point. For scenarios A1 and B1, the simple effects/variance scenarios, independent QTL effects were drawn according to the following structure:

$$
\boldsymbol{\Sigma}_{\boldsymbol{GL}}^{\mathbf{relG}} = \begin{bmatrix} \sigma^2_{GL^{\text{relG}}} & 0 & \dots & 0 \\ 0 & \sigma^2_{GL^{\text{relG}}} & \dots & 0 \\ \vdots & \vdots & \ddots & \vdots \\ 0 & 0 & \dots & \sigma^2_{GL^{\text{relG}}} \end{bmatrix} \qquad \boldsymbol{\Sigma}_{\boldsymbol{GY}}^{\mathbf{relG}} = \begin{bmatrix} \sigma^2_{GY^{\text{relG}}} & 0 \\ 0 & \sigma^2_{GY^{\text{relG}}} \end{bmatrix}
$$

Where there is a unique variance for GL and GY, set relative to the genetic/additive variance. Now it is straightforward to demonstrate how GLY was simulated for the simple effects scenarios (A's) based on the following covariance matrices:

$$
\boldsymbol{\Sigma}_{\boldsymbol{GLY}}^{\mathbf{relG}} = \begin{bmatrix} \sigma^2_{GLY^{\text{relG}}} & 0 & \dots & 0 \\ 0 & \sigma^2_{GLY^{\text{relG}}} & \dots & 0 \\ \vdots & \vdots & \ddots & \vdots \\ 0 & 0 & \dots & \sigma^2_{GLY^{\text{relG}}} \end{bmatrix}
$$

And finally for the complex effects scenarios (B's):

$$\Sigma_{GLY}^{\mathbf{relG}} = \begin{bmatrix} \sigma^2_{GLY_1{}^{\text{relG}}} & 0 & \cdots & 0 \\ 0 & \sigma^2_{GLY_2{}^{\text{relG}}} & \cdots & 0 \\ \vdots & \vdots & \ddots & \vdots \\ 0 & 0 & \cdots & \sigma^2_{GLY_{20}{}^{\text{relG}}} \end{bmatrix}$$

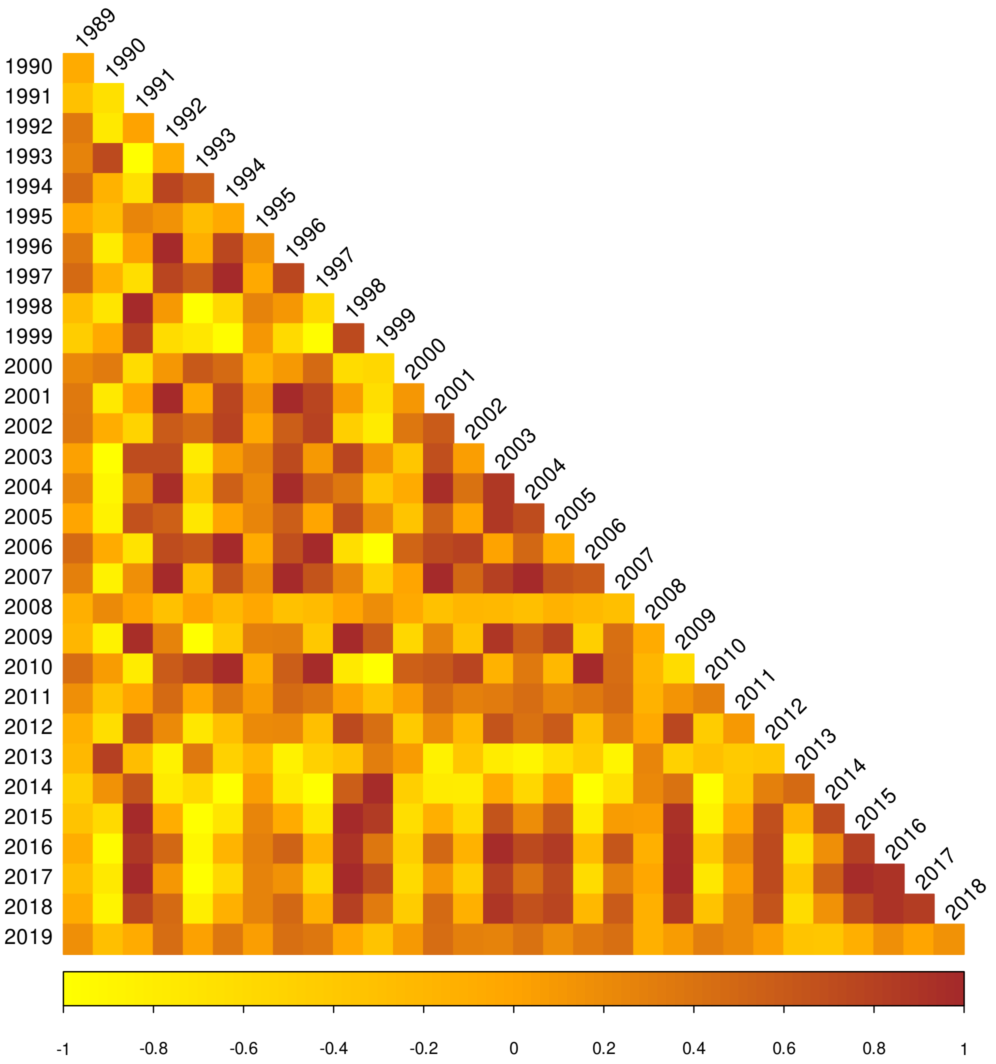

Figure D1: Phenotypic correlations between years (ρ_{GY}^{FA}) estimated from FA multiplicative model. For details, check Model M3-18 from Krause et al. (2023).

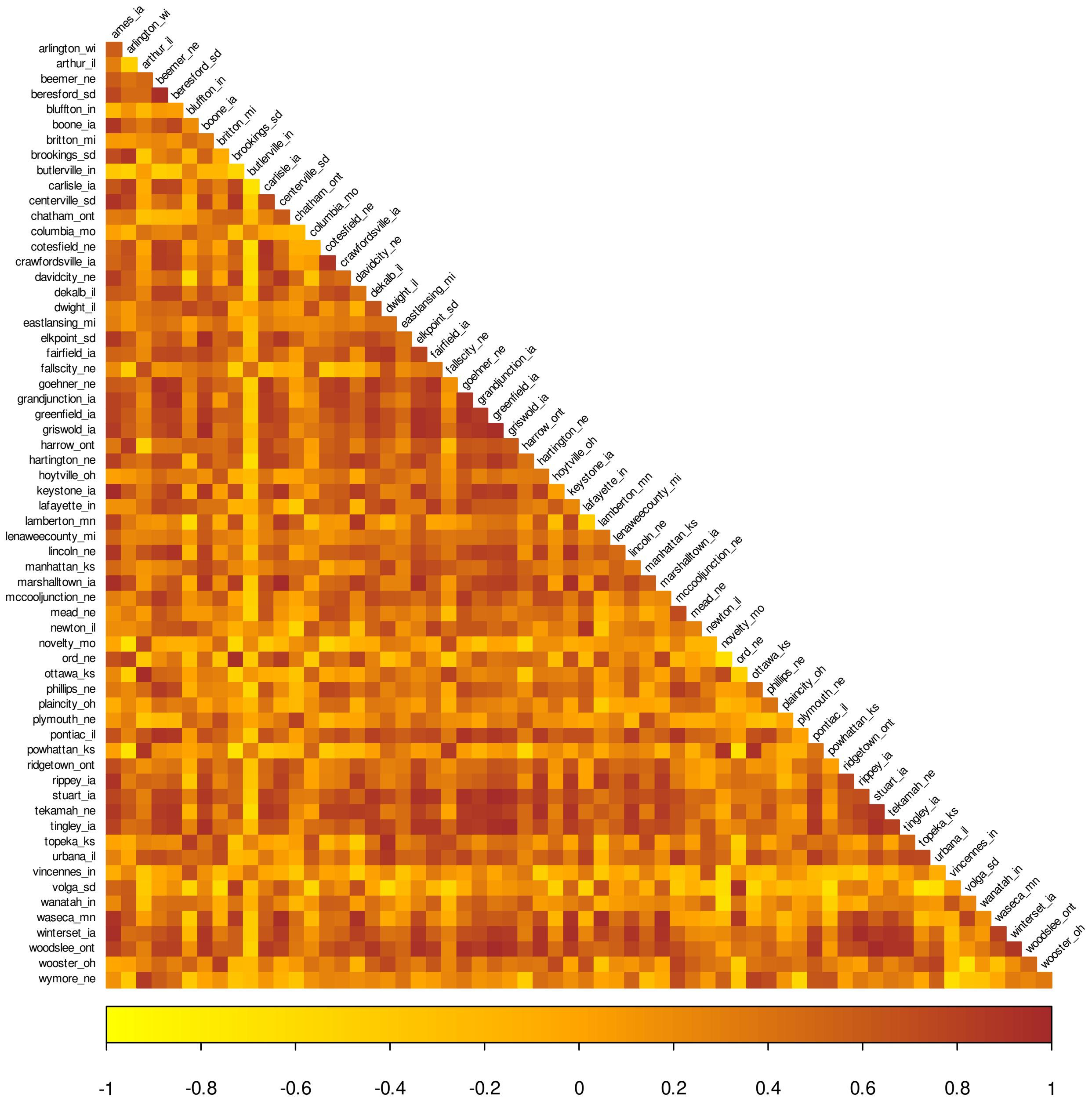

Figure D2: Phenotypic correlations between locations (ρ_{GL}^{FA}) estimated from FA multiplicative model. For details, check Model M3-18 from Krause et al. (2023).

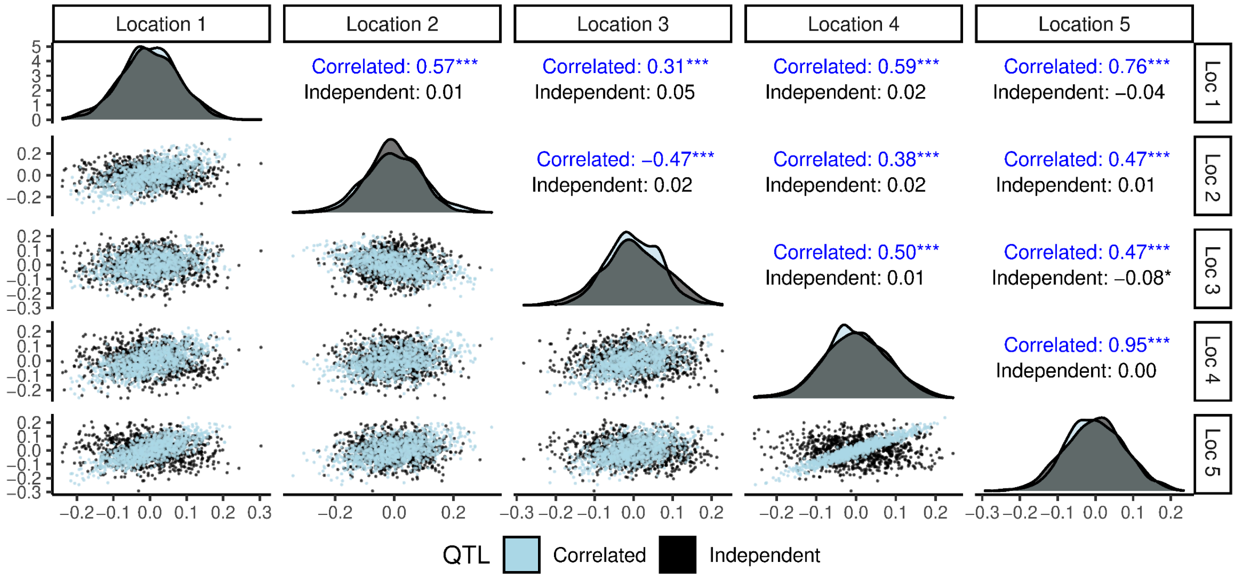

Figure D3: An example of sampling of QTL effects according to correlated or independent genotype × location interaction effects. Pearson correlation coefficients followed by their significant tests are plotted in the upper diagonal.

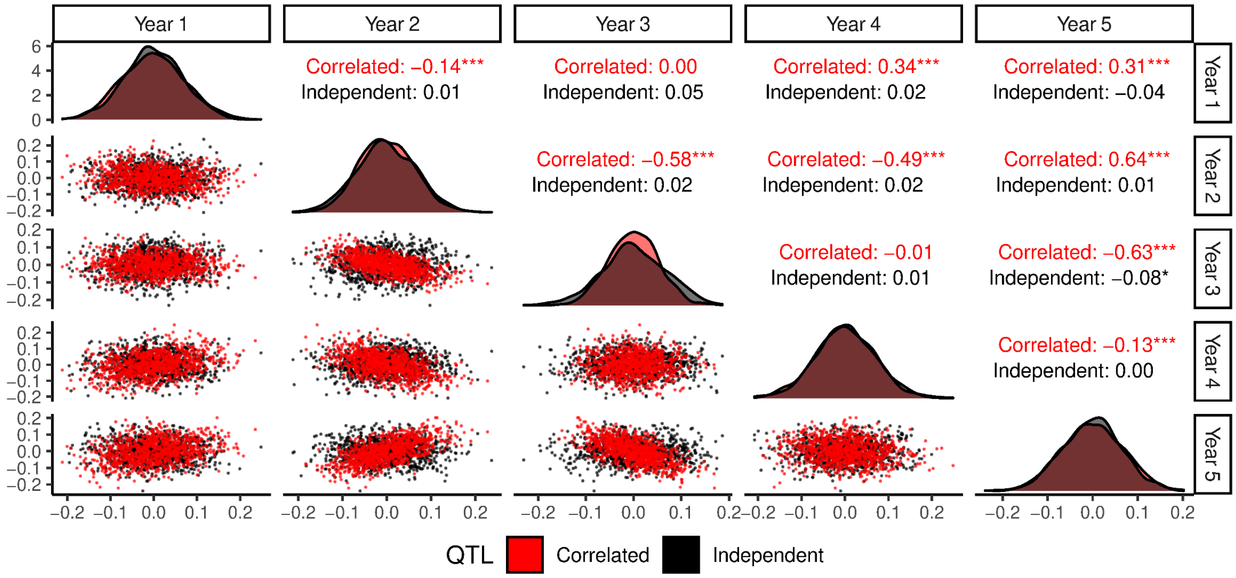

Figure D4: An example of sampling of QTL effects according to correlated or independent genotype × year interaction effects. Pearson correlation coefficients followed by their significant tests are plotted in the upper diagonal.

E - Empirical density functions of variance components

This section describes the rationale and objective of estimating probability density functions for the underlying trend of genotypic (σ^2_G), genotype by location (σ^2_{GL}), genotype by year (σ^2_{GY}), genotype by location by year (σ^2_{GLY}), and residual (σ^2_ϵ) variance components derived from linear mixed models. We envision fitting parametric probability distributions to variance components to capture the GEI trend to be used in future simulation studies, needed for predicting plant breeding outcomes in changing climates. Currently, simulation studies rely on point estimates of variance components (Kleinknecht et al. 2016), or set heritability values such as low or high (Rutkoski 2019). By capturing the GEI trend using historical data, we can conduct more realistic simulation studies.

E.1 - Methods

A modified jackknife resampling approach was used to obtain empirical probability distributions for the variance components σ^2_G, σ^2_{GL}, σ^2_{GY}, and σ^2_{GLY}. Utilizing results from Aguate et al. (2019) and Hartung and Piepho (2021), the empirical data set from Krause et al. (2023) was divided into four groups representing consecutive eras of soybean cultivar development: From 1989 to 1995, from 1996 to 2003, from 2004 to 2011, and from 2012 to 2019 (see Discussion below). For the first group (1989-1995), there were 181 environments (location-year combination); for 1996-2003, 194 environments; for 2004-2011, 100 environments; and for 2012-2019, 116 environments. The modified jackknife approach consisted of leaving-one-environment out (instead of one observation), and then obtaining REML estimates of variance components with a modified version of Model C1 that considered all terms as random effects. Estimates of variance components were combined and evaluated for a best-fit probability distribution with the package "ForestFit" (Teimouri 2021). Given the lack of data from individual plots, trial-based estimates of σ^2_ϵ from the first-stage of analyses were used (i.e., no resampling). Check Krause et al. (2023) for further details on the statistical modeling of the phenotypic data. Distributional parameters were estimated via the expectation maximization (EM) algorithm (Dempster et al. 1977) using the log-likelihood functions of the Gamma, Log-Logistic, Log-Normal, Burr, and F univariate and multivariate distributions. In addition to a visual comparison of the modeled distributions relative to the empirical distributions, Akaike (AIC) and Bayesian (BIC) information criteria (Akaike 1974; Schwarz 1978) as well as the Kolmogorov-Smirnov (KS), Cramer-von Mises (CM), and Anderson-Darling (AD) goodness-of-fit statistics (Stephens 1986) were considered to select the best-fit distribution for each variance component. A classical penalized criteria based on the log-likelihood (AIC, BIC) provided protection from overfitting.

E.2 - Results

Estimated variance components revealed distinct multi-modal distributions over time (Figure E1). For example, the estimated σ^2_G more than doubled from ~ 3.54 (bu/ac)^2 for the period 1989-1995 to ~ 7.56 (bu/ac)^2 for the period 1996-2003. While the smallest

magnitudes of estimated GEI variance components were usually associated with the period from 1989 to 1995, subsequent changes across years were unique to each estimated variance component. The best-fit models for the trend of variance components consisted of a mixture of five Log-Logistic distributions for the empirical distribution of σ_G^2, three Log-Logistic distributions for the empirical distribution of σ_{GL}^2, six Log-Logistic distributions for the empirical distribution of σ_{GY}^2, and five Gamma distributions for the empirical distribution of σ_{GLY}^2 (Table E1, Figure E2). For the empirical distribution of estimated residual variances (σ_ϵ^2), obtained directly rather than through jackknife resampling, the best-fit model was a univariate Log-Logistic distribution (Figure E3). Maximum Likelihood estimates for the distributional model parameters are reported for the selected models (Table E1), as well as plots with empirical and fitted cumulative distribution functions (Figures E4 and E5). All evaluated models are given in Table E2.

E.3 - Discussion

Parametric probability distributions were fitted to variance components by dividing the data set into groups of seven to eight years. Aguate et al. (2019) simulated different MET with variable numbers of genotypes, locations, and years to mimic wheat trials. They found that adding years is more beneficial than adding genotypes or locations for obtaining unbiased estimates of genotypic-related variance components. Furthermore, even in highly imbalanced datasets, estimates from at least eight years of trials produced less than 5% bias in the estimates, compared to biases of ~18% for σ_G^2, >40% for σ_{GL}^2, and >15% for σ_{GY}^2, when only two years of MET were considered. Simulation results from Hartung and Piepho (2021) further demonstrated that non-significant bias could be obtained for estimates of σ_G^2, σ_{GL}^2, and σ_{GY}^2, with decreasing dropout rate and increasing the number of years of testing. Both references showed that variance estimates from REML are biased if at least one variance component estimate is bounded at zero, which rarely occurs when using seven or eight years of data. The reader should keep in mind that the goal of minimizing bias in estimators is distinct from the magnitude and proportion of estimates of variance components. The former is a concern for algorithmic estimators while the latter is of concern for breeding decisions about whether to use more locations or more years in their crop species and for traits under selection.

In terms of the fitted probability distributions, especially due to the well-known properties of the analysis of variance (ANOVA) among the breeding community, there is a misconception regarding the distribution of estimates of variance components. If the underlying population is normally distributed, the mean squares are distributed as a chi-square (χ^2), whereas normality and independence are requirements to compute valid F tests from ANOVA (Rencher and Schaalje 2007, Chapter 5). The χ^2 distribution is further used for inferences about variance uncertainty, but as mentioned, it does assume that the random variable is normally distributed. The computed empirical distributions in this work can be of any form (distribution), likewise, other approaches such as hierarchical Bayesian models can be employed to obtain posterior distributions of variance estimates. It should be emphasized, however, that the obtained empirical density functions might not necessarily be the data-generating functions. When testing the fit of distributions, the

systematic dropping of the data for curation and cleaning (6.8% of the data points were removed, see Krause et al. (2023) for details) was not considered. In other words, variance components are simulated from the conditional distribution that holds after trimming.

E.4 - Tables and Figures

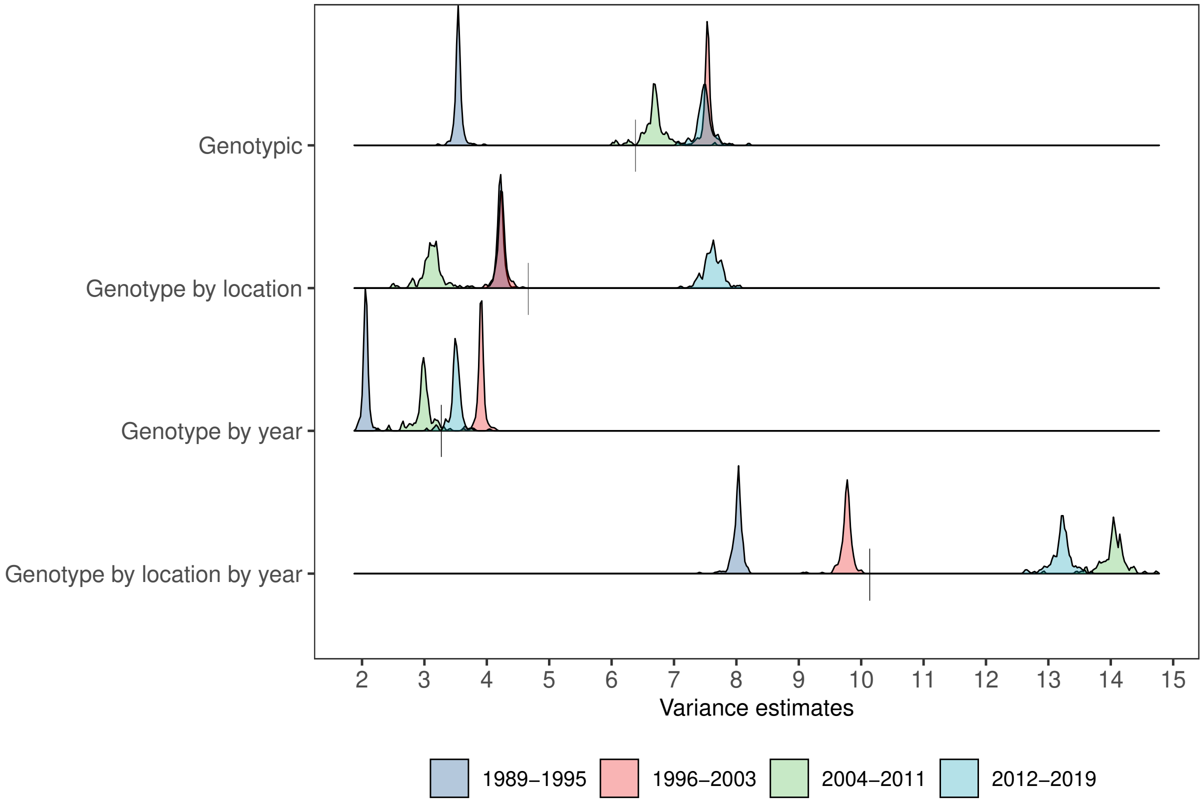

Figure E1: Empirical distributions of estimated variance components consisting of genotypic, genotype by location, genotype by year, and genotype by location by year variances for groups of years 1989-1995, 1996-2003, 2004-2011, and 2012-2019. Empirical estimates were obtained using a jackknife leave-one-location-out method. Vertical bars on the x-axis represent point estimates across all years.

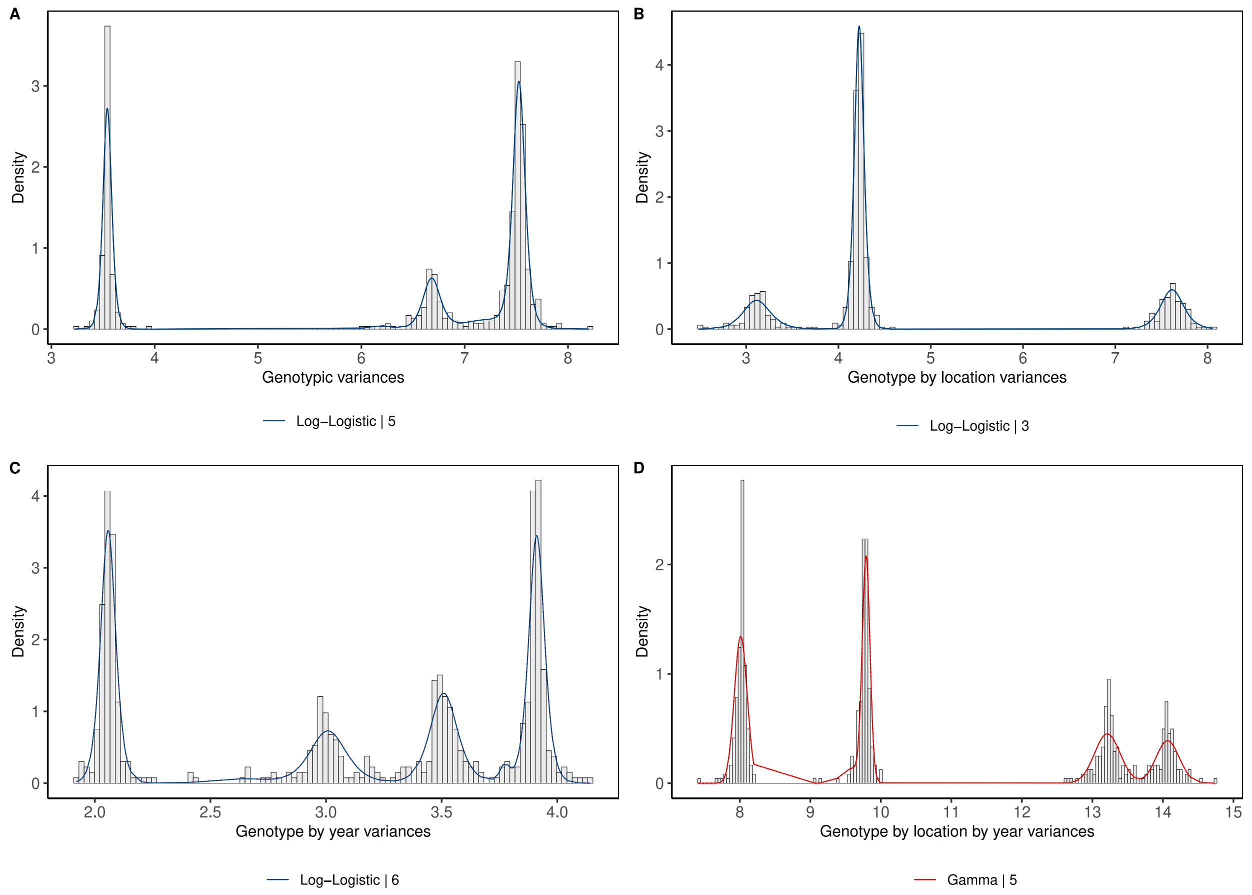

Figure E2: Empirical distributions and probability density function (PDF) of variance components for genotypic (A), genotype by location (B), genotype by year (C), and genotype by location by year (D). Empirical estimates were obtained using a jackknife leave-one-environment-out method. The best-fit models are presented with different colors and include the distribution's name and its mixture number.

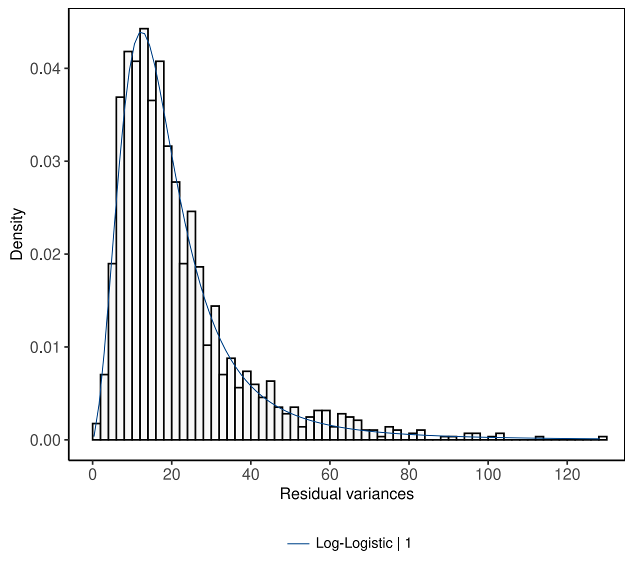

Figure E3: Empirical distribution and probability density function (PDF) for the unimodal Log-Logistic model of residual estimates from individual trials.

Table E1: Maximum Likelihood estimates of parameters for the best-fit univariate and multivariate probability distributions for empirical distributions obtained using jackknife resampling. Estimates of residual variance (σ_ϵ^2) were obtained from trials conducted from 1989 to 2019.

Variance[a]	Distribution	Number of distributions	Parameters[b]		
			$\hat{w}_i$	$\hat{\alpha}_i$	$\hat{\beta}_i$
			0.30	125.98	3.54
			0.14	119.87	6.68
σ_G^2	Log-Logistic	5	0.08	44.03	7.23
			0.01	88.03	6.18
			0.47	192.69	7.53
			0.63	122.27	4.23
σ_{GL}^2	Log-Logistic	3	0.17	32.00	3.12
			0.20	92.56	7.62
			0.02	258.41	3.77
			0.31	172.61	3.91
σ_{GY}^2	Log-Logistic	6	0.20	89.05	3.51
			0.30	94.97	2.06
			0.02	32.81	2.64
			0.15	57.79	3.01
			0.05	4078.04	0.0024
			0.31	7755.00	0.0011
σ_{GLY}^2	Gamma	5	0.20	5306.62	0.0025
			0.28	31922.50	0.0003
			0.16	7301.02	0.0020
σ_ϵ^2	Log-Logistic	1	1	2.56	17.03

[a] Genotypic (σ_G^2), genotype × location (σ_{GL}^2), genotype × year (σ_{GY}^2), and genotype × location × year (σ_{GLY}^2) variance components.
[b] Estimates of weight parameters (w_i) sums to one, and both Gamma and Log-Logistic distributions include a shape (α_i) and scale (β_i) parameter.

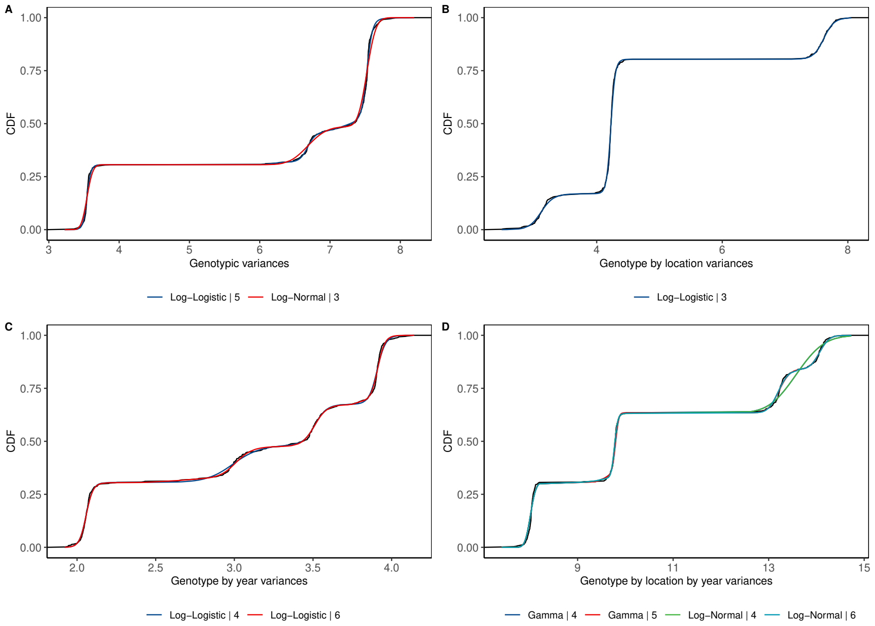

Figure E4: Cumulative distribution function (CDF) for the best-fit models according to the genotypic (A), genotype by location (B), genotype by year (C), and genotype by location by year (D) variances from jackknife. In each plot, the legends states for name of the distribution followed by its mixture number.

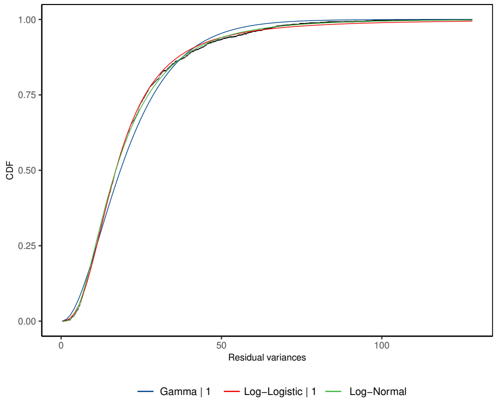

Figure E5: Cumulative distribution function (CDF) for the best-fit models according to the residual variances from individual trial level. The legends state the name of the distribution followed by its mixture number.

Table E2: Goodness-of-fit (GOF) statistics and selection criteria for the fit of univariate and multivariate probability distributions for genotypic, genotype by location, genotype by year, and genotype by location by year variance components estimated from the jackknife analysis, and residuals variances from trial-level. The best-fit model is highlighted in bold. KS, CM, AD, AIC, and BIC stand for Kolmogorov-Smirnov, Cramer-von Mises, Anderson-Darling, Akaike's and Bayesian Information Criterion, respectively.

Variance	Distribution	Mixture of	GOF statistics			GOF criteria		
			KS	CM	AD	AIC	BIC	Log-likelihood
	Gamma	2	0.19	4.39	25.23	672.09	694.00	-331.05
	Log-Logistic	2	0.15	3.32	19.97	598.57	620.48	-294.29
	Log-Normal	2	0.19	4.48	25.66	680.36	702.27	-335.18
	Burr	2	0.50	40.79	193.24	3978.69	4000.60	-1984.35
	F	2	0.67	77.08	356.80	4338.62	4360.52	-2164.31
	Gamma	3	0.07	0.81	5.56	182.11	217.16	-83.06
	Log-Logistic	3	0.15	3.26	19.45	554.84	589.90	-269.42
	Log-Normal	3	0.08	0.66	4.90	170.74	205.79	-77.37
	Burr	3	0.52	44.39	207.91	3978.03	4013.09	-1981.02
	F	3	0.67	77.08	356.80	4338.62	4360.52	-2164.31
	Gamma	4	0.09	1.03	6.20	177.38	225.58	-77.69
	Log-Logistic	4	0.15	3.26	19.45	565.62	613.82	-271.81
Genotypic	Log-Normal	4	0.08	0.52	3.61	74.67	122.87	-26.33
	Burr	4	0.52	44.39	207.91	3984.04	4032.24	-1981.02
	F	4	0.67	75.30	347.50	4351.57	4399.77	-2164.79
	Gamma	5	0.10	0.74	5.85	155.12	216.47	-63.56
	**Log-Logistic**	**5**	0.05	0.21	1.67	-9.63	51.71	18.82
	Log-Normal	5	0.06	0.36	2.53	89.40	150.75	-30.70
	Burr	5	0.53	45.19	211.25	3989.54	4050.89	-1980.77
	F	5	0.67	75.93	350.80	4357.11	4418.45	-2164.55
	Gamma	6	0.07	0.46	3.15	94.30	168.79	-30.15
	Log-Logistic	6	0.05	0.20	1.89	32.97	107.47	0.51
	Log-Normal	6	0.06	0.37	2.68	103.17	177.66	-34.59
	Burr	6	0.52	43.68	205.00	3996.91	4071.40	-1981.45
	F	6	0.66	74.44	343.11	4364.38	4438.87	-2165.19
	Gamma	2	0.30	10.51	48.78	1164.59	1186.50	-577.29
Genotype by Location	Log-Logistic	2	0.24	8.35	49.77	1139.99	1161.90	-565.00
	Log-Normal	2	0.30	10.80	50.82	1194.97	1216.87	-592.48
	Burr	2	0.50	42.63	197.90	3466.48	3488.39	-1728.24
	F	2	0.63	67.82	305.85	3830.34	3852.24	-1910.17
	Gamma	3	0.30	10.52	48.87	1185.88	1220.94	-584.94
	**Log-Logistic**	**3**	0.04	0.14	0.72	-80.42	-45.36	48.21
	Log-Normal	3	0.06	0.49	2.38	-5.10	29.96	10.55
	Burr	3	0.50	42.78	198.50	3477.58	3512.63	-1730.79
	F	3	0.63	67.82	305.85	3830.34	3852.24	-1910.17
	Gamma	4	0.30	10.51	48.83	1185.69	1233.89	-581.85
	Log-Logistic	4	0.04	0.13	0.62	-80.72	-32.52	51.36
	Log-Normal	4	0.06	0.48	2.20	-10.68	37.52	16.34
	Burr	4	0.50	41.96	195.32	3482.35	3530.55	-1730.18
	F	4	0.65	73.19	331.38	3854.78	3902.98	-1916.39
	Gamma	5	0.02	0.07	0.56	-34.66	26.69	31.33
	Log-Logistic	5	0.03	0.10	0.59	-73.99	-12.64	50.99

Table E2 continued from previous page

Variance	Distribution	Mixture of	GOF statistics			GOF criteria		
			KS	CM	AD	AIC	BIC	Log-likelihood
	Log-Normal	5	0.02	0.06	0.49	-66.28	-4.93	47.14
	Burr	5	0.49	41.22	192.48	3488.02	3549.36	-1730.01
	F	5	0.65	72.56	328.33	3860.42	3921.76	-1916.21
	Gamma	6	0.02	0.06	0.40	-30.91	43.58	32.46
	Log-Logistic	6	0.03	0.09	0.46	-74.96	-0.47	54.48
	Log-Normal	6	0.03	0.04	0.25	-71.57	2.92	52.78
	Burr	6	0.49	41.61	194.00	3494.23	3568.72	-1730.11
	F	6	0.65	72.49	328.02	3866.55	3941.05	-1916.28
Genotype by Year	Gamma	2	0.13	2.33	17.77	805.22	827.13	-397.61
	Log-Logistic	2	0.12	1.39	10.73	522.98	544.89	-256.49
	Log-Normal	2	0.13	1.80	12.89	546.28	568.19	-268.14
	Burr	2	0.46	34.26	165.40	2641.18	2663.09	-1315.59
	F	2	0.67	80.39	375.82	3042.72	3064.63	-1516.36
	Gamma	3	0.12	1.48	10.61	438.20	473.26	-211.10
	Log-Logistic	3	0.08	0.29	1.48	69.69	104.75	-26.85
	Log-Normal	3	0.13	1.36	10.01	402.06	437.11	-193.03
	Burr	3	0.49	38.99	184.26	2636.35	2671.40	-1310.17
	F	3	0.67	80.39	375.82	3042.72	3064.63	-1516.36
	Gamma	4	0.12	1.34	9.44	391.85	440.05	-184.92
	Log-Logistic	4	0.04	0.11	1.14	-21.98	26.22	21.99
	Log-Normal	4	0.05	0.26	2.48	69.53	117.73	-23.77
	Burr	4	0.48	36.59	174.58	2642.16	2690.36	-1310.08
	F	4	0.65	73.44	338.37	3062.06	3110.26	-1520.03
	Gamma	5	0.07	0.45	4.18	142.71	204.05	-57.35
	Log-Logistic	5	0.03	0.09	1.07	-25.40	35.94	26.70
	Log-Normal	5	0.05	0.27	2.52	73.76	135.10	-22.88
	Burr	5	0.46	33.87	163.84	2652.24	2713.58	-1312.12
	F	5	0.65	73.45	338.42	3068.16	3129.51	-1520.08
	Gamma	6	0.05	0.29	2.90	78.16	152.65	-22.08
	**Log-Logistic**	**6**	0.03	0.08	1.04	-35.86	38.63	34.93
	Log-Normal	6	0.04	0.13	1.11	2.00	76.49	16.00
	Burr	6	0.45	32.15	157.07	2645.95	2720.44	-1305.98
	F	6	0.65	73.41	338.24	3073.76	3148.25	-1519.88
Genotype by Location by Year	Gamma	2	0.18	3.72	23.32	2027.94	2049.84	-1008.97
	Log-Logistic	2	0.17	3.40	21.40	2104.98	2126.88	-1047.49
	Log-Normal	2	0.18	3.73	23.33	2028.46	2050.36	-1009.23
	Burr	2	0.57	46.63	216.45	4962.90	4984.81	-2476.45
	F	2	0.68	68.15	308.55	5298.60	5320.51	-2644.30
	Gamma	3	0.06	0.52	3.94	905.88	940.93	-444.94
	Log-Logistic	3	0.17	3.22	19.52	1938.73	1973.78	-961.36
	Log-Normal	3	0.06	0.52	3.96	909.36	944.42	-446.68
	Burr	3	0.60	51.37	235.46	4969.12	5004.18	-2476.56
	F	3	0.68	68.15	308.57	5304.63	5339.68	-2644.31
	Gamma	4	0.06	0.41	3.50	830.66	878.86	-404.33
	Log-Normal	4	0.06	0.36	3.30	810.14	858.34	-394.07
	Burr	4	0.60	51.37	235.46	4975.11	5023.31	-2476.55
	F	4	0.69	71.27	323.14	5313.82	5362.02	-2645.91
	**Gamma**	**5**	0.05	0.21	1.74	709.98	771.33	-340.99
	Log-Normal	5	0.06	0.22	1.15	715.12	776.47	-343.56

Table E2 continued from previous page

Variance	Distribution	Mixture of	GOF statistics			GOF criteria		
			KS	CM	AD	AIC	BIC	Log-likelihood
	Burr	5	0.60	51.37	235.46	4981.10	5042.45	-2476.55
	F	5	0.70	75.75	344.66	5317.16	5378.51	-2644.58
	Gamma	6	0.06	0.32	2.58	781.22	855.71	-373.61
	Log-Normal	6	0.05	0.16	1.53	691.83	766.32	-328.91
	Burr	6	0.58	48.52	223.96	4989.49	5063.98	-2477.75
	F	6	0.68	68.92	312.13	5322.81	5397.30	-2644.40
Residual (Trial-level)	Gamma	1	0.06	1.60	9.57	11139.50	11150.02	-5567.80
	**Log-Logistic**	**1**	0.02	0.07	0.72	11063.82	11074.34	-5529.90
	Log-Normal	1	0.02	0.09	0.67	11066.60	11077.12	-5531.10
	Burr	1	0.41	79.00	377.80	13896.60	13907.11	-6946.30
	F	1	0.54	131.37	594.40	14677.45	14687.97	-7336.73

F - Stage-wise analysis

All models were obtained via stage-wise analysis with weights equal to $1/SE^2$, where SE is the standard error of genotypic means (Frensham et al. 1997; Smith et al. 2001). Stage-wise models break down the full analysis (i.e., single-stage analysis) into multiple steps, by carrying to the next step the estimated means and some measure of precision (Frensham et al. 1997; Smith et al. 2001; Piepho et al. 2012).

G - Model E9

Model E9 (Table 6) is based on the methodology proposed by Vencosvsky et al. (1986) and later modified/applied by Brisson et al. (2010), Oury et al. (2012), and Bornhofen et al. (2018). This model takes advantage of both experimental genotypes and checks replicated between consecutive pairs of years: (i) an initial model is fitted within years to predict eBLUP values of genotypic means (g_{ik}); and (ii) the g_{ik} values are then corrected for the year effect according to the "reference year (R)". For example, in a MET dataset composed of 10 years, if R = year 5, the corrections for the year effects will be performed like $1 \leftarrow 2 \leftarrow 3 \leftarrow 4 \leftarrow 5 \rightarrow 6 \rightarrow 7 \rightarrow 8 \rightarrow 9 \rightarrow 10$, a forward-backward process along years. If $R = 1$, it is only a forward process ($1 \rightarrow \cdots \rightarrow 10$), and if $R = 10$, a backward process ($1 \leftarrow \cdots \leftarrow 10$). The algorithm is described with the following equations:

$$gc_{ik} = g_{ik} + d_{R,R\pm1} \tag{G1}$$

where gc_{ik} is the corrected value for genotype i in year k, and $d_{R,R\pm1}$ is the correction term obtained by Equation G2:

$$d_{R,R\pm1} = \frac{\sum_{i=1}^{n_{R,R\pm1}} (g_{iR} - g_{iR\pm1})}{n_{R,R\pm1}} \tag{G2}$$

where $n_{R,R\pm1}$ is the number of common genotypes in years R and $R\pm1$. The $\pm$ sign states the correction can go forward or backward across years. In the following year ($R\pm2$), the procedure is the same:

$$gc_{ik\pm2} = gc_{ik\pm1} + d_{R\pm1,R\pm2} \tag{G3}$$

where $d_{R\pm1,R\pm2}$ is:

$$d_{R\pm1,R\pm2} = \frac{\sum_{i=1}^{n_{R\pm1,R\pm2}} (gc_{iR\pm1} - g_{iR\pm2})}{n_{R\pm1,R\pm2}} \tag{G4}$$

and so on for the following years. The estimated "year effects" ($\hat{d}_{R,R\pm1}$, $\hat{d}_{R\pm1,R\pm2}$, ..., $\hat{d}_{R\pm k-1,R\pm K}$) are calculated relatively to the reference year, R. In our simulations, the reference year was randomly chosen among the five years with the highest generalized measure of heritability (Cullis et al. 2006, H^2), calculated as:

$$H_k^2 = 1 - \frac{\bar{v}_k^{BLUP}}{2\sigma_{G_k}^2} \tag{G5}$$

where $\bar{v}_k^{BLUP}$ is the average pairwise genotypic prediction error variance and $\sigma_{G_k}^2$ is the genotypic variance estimated from residual maximum likelihood (Patterson and Thompson 1971, REML). In step (i), before correction, the intercept (μ) was added to the eBLUP values. μ was parameterized to represent the average least-square value of the locations observed that year.

References

Aguate, F., J. Crossa, and M. Balzarini. 2019. Effect of missing values on variance component estimates in multienvironment trials. *Crop Science 59*(2): 508–517. https://doi.org/10.2135/cropsci2018.03.0209 .

Akaike, H. 1974. A New Look at the Statistical Model Identification. *IEEE Transactions on Automatic Control 19*(6): 716–723. https://doi.org/10.1109/TAC.1974.1100705 .

Bates, D. and M. Maechler. 2021. Matrix: Sparse and Dense Matrix Classes and Methods.

Bornhofen, E., M.H. Todeschini, M.G. Stoco, A. Madureira, V.S. Marchioro, L. Storck, and G. Benin. 2018. Wheat yield improvements in Brazil: Roles of genetics and environment. *Crop Science 58*(3): 1082–1093. https://doi.org/10.2135/cropsci2017.06.0358 .

Brisson, N., P. Gate, D. Gouache, G. Charmet, F.X. Oury, and F. Huard. 2010. Why are wheat yields stagnating in Europe? A comprehensive data analysis for France. *Field Crops Research 119*(1): 201–212. https://doi.org/10.1016/j.fcr.2010.07.012 .

Cullis, B.R., A.B. Smith, and N.E. Coombes. 2006. On the design of early generation variety trials with correlated data. *Journal of Agricultural, Biological, and Environmental Statistics 11*(4): 381–393. https://doi.org/10.1198/108571106X154443.

Dempster, A.P., N.M. Laird, and D.B. Rubin. 1977. Maximum Likelihood from Incomplete Data Via the EM Algorithm. *Journal of the Royal Statistical Society: Series B (Methodological) 39*(1): 1–38. https://doi.org/10.1111/j.2517-6161.1977.tb01600.x.

Frensham, A., B. Cullis, and A. Verbyla. 1997. Genotype by Environment Variance Heterogeneity in a Two-Stage Analysis. *Biometrics 53*(4): 1373–1383 .

Gaynor, R.C., G. Gorjanc, and J.M. Hickey. 2021. AlphaSimR: An R package for breeding program simulations. *G3: Genes, Genomes, Genetics 11*(2). https://doi.org/10.1093/G3JOURNAL/JKAA017.

Hardin, J., S.R. Garcia, and D. Golan. 2013, 9. A method for generating realistic correlation matrices. *The Annals of Applied Statistics 7*(3). https://doi.org/10.1214/13-AOAS638.

Hartung, J. and H. Piepho. 2021. Effect of missing values in multi-environmental trials on variance component estimates. *Crop Science* (August): 1–11. https://doi.org/10.1002/csc2.20621.

Kleinknecht, K., J. Möhring, F. Laidig, U. Meyer, and H.P. Piepho. 2016. A simulation-based approach for evaluating the efficiency of multienvironment trial designs. *Crop Science 56*(5): 2237–2250. https://doi.org/10.2135/cropsci2015.07.0405.

Krause, M.D., K.O.G. Dias, A.K. Singh, and W.D. Beavis. 2023. Using soybean historical field trial data to study genotype by environment variation and identify mega-environments with the integration of genetic and non-genetic factors. *bioRxiv*. https://doi.org/10.1101/2022.04.11.487885.

Oury, F.X., C. Godin, A. Mailliard, A. Chassin, O. Gardet, A. Giraud, E. Heumez, J.Y. Morlais, B. Rolland, M. Rousset, M. Trottet, and G. Charmet. 2012. A study of genetic progress due to selection reveals a negative effect of climate change on bread wheat yield in France. *European Journal of Agronomy* 40: 28–38. https://doi.org/10.1016/j.eja.2012.02.007.

Patterson, H.D. and R. Thompson. 1971. Recovery of inter-block information when block sizes are unequal. *Biometrika 58*(23): 545–554. https://doi.org/doi:10.1093/biomet/58.3.545.

Piepho, H.P., J. Möhring, T. Schulz-Streeck, and J.O. Ogutu. 2012. A stage-wise approach for the analysis of multi-environment trials. *Biometrical Journal 54*(6): 844–860. https://doi.org/10.1002/bimj.201100219.

Rencher, A.C. and G.B. Schaalje. 2007, 12. *Linear Models in Statistics*. Hoboken, NJ, USA: John Wiley & Sons, Inc.

Rutkoski, J.E. 2019. Estimation of realized rates of genetic gain and indicators for breeding program assessment. *Crop Science 59*(3): 981–993. https://doi.org/10.2135/cropsci2018.09.0537 .

Schwarz, G. 1978. Estimating the dimension of a model. *The Annals of Statistics 6*(2): 461–464. https://doi.org/10.1214/aos/1176344136 .

Smith, A., B. Cullis, and A. Gilmour. 2001. The analysis of crop variety evaluation data in Australia. *Australian and New Zealand Journal of Statistics 43*(2): 129–145. https://doi.org/10.1111/1467-842X.00163 .

Smith, A., B.R. Cullis, and R. Thompson. 2001. Analyzing variety by environment data using multiplicative mixed models and adjustments for spatial field trend. *Biometrics 57*(4): 1138–1147. https://doi.org/10.1111/j.0006-341X.2001.01138.x .

Stephens, M. 1986. Tests based on edf statistics, In *Goodness-of-fit techniques*, eds. D'Agostino, R. and M. Stephens, 97–194. New York: Marcel Dekker.

Teimouri, M. 2021. ForestFit: Statistical Modelling for Plant Size Distributions.

Vencosvsky, R., A. Morales, J.C. Garcia, and N.M. Teixeira 1986. Progresso Genético Em Vinte Anos De Melhoramento Do Milho No Brasil. In *Anais do congresso nacional de milho e sorgo*, Belo Horizonte, Minas Gerais, pp. 300–307. Embrapa-CNPMS, Sete Lagoas, Minas Gerais, Brazil.

Yan, W. 2016. Analysis and handling of G x E in a practical breeding program. *Crop Science* 56: 2106–2118. https://doi.org/10.2135/cropsci2015.06.0336 .
